# Genomics- and Metabolomics-Based Investigation of the Deep-Sea Sediment-Derived Yeast, *Rhodotorula mucilaginosa* 50-3-19/20B

**DOI:** 10.3390/md19010014

**Published:** 2020-12-30

**Authors:** Larissa Buedenbender, Abhishek Kumar, Martina Blümel, Frank Kempken, Deniz Tasdemir

**Affiliations:** 1 GEOMAR Centre for Marine Biotechnology (GEOMAR-Biotech), Research Unit Marine Natural Products Chemistry, GEOMAR Helmholtz Centre for Ocean Research Kiel, Am Kiel-Kanal 44, 24106 Kiel, Germany; larissa.buedenbender@alumni.griffithuni.edu.au (L.B.); mbluemel@geomar.de (M.B.); 2 Institute of Bioinformatics, International Technology Park, Bangalore, 560066 & Manipal Academy of Higher Education (MAHE), Manipal 576104, Karnataka, India; abhishek@ibioinformatics.org; 3 Department of Botany, Kiel University, Olshausenstr. 40, 24098 Kiel, Germany; 4 Faculty of Mathematics and Natural Sciences, Kiel University, Christian-Albrechts-Platz 4, 24118 Kiel, Germany

**Keywords:** *Rhodotorula*, deep-sea sediment, yeast, metabolomics, GNPS molecular networking, dereplication, polyol esters of fatty acids, anticancer, genome sequencing, exo-inulinase enzyme

## Abstract

Red yeasts of the genus *Rhodotorula* are of great interest to the biotechnological industry due to their ability to produce valuable natural products, such as lipids and carotenoids with potential applications as surfactants, food additives, and pharmaceuticals. Herein, we explored the biosynthetic potential of *R. mucilaginosa* 50-3-19/20B collected from the Mid-Atlantic Ridge using modern genomics and untargeted metabolomics tools. *R. mucilaginosa* 50-3-19/20B exhibited anticancer activity when grown on PDA medium, while antimicrobial activity was observed when cultured on WSP-30 medium. Applying the bioactive molecular networking approach, the anticancer activity was linked to glycolipids, namely polyol esters of fatty acid (PEFA) derivatives. We purified four PEFAs (**1**–**4**) and the known methyl-2-hydroxy-3-(1H-indol-2-yl)propanoate (**5**). Their structures were deduced from NMR and HR-MS/MS spectra, but **1**–**5** showed no anticancer activity in their pure form. Illumina-based genome sequencing, *de novo* assembly and standard biosynthetic gene cluster (BGC) analyses were used to illustrate key components of the PEFA biosynthetic pathway. The fatty acid producing BGC3 was identified to be capable of producing precursors of PEFAs. Some *Rhodotorula* strains are able to convert inulin into high-yielding PEFA and cell lipid using a native exo-inulinase enzyme. The genomic locus for an exo-inulinase enzyme (g1629.t1), which plays an instrumental role in the PEFA production via the mannitol biosynthesis pathway, was identified. This is the first untargeted metabolomics study on *R. mucilaginosa* providing new genomic insights into PEFA biosynthesis.

## 1. Introduction

Marine environments provide a great resource for biotechnology. In particular, marine microorganisms offer a sustainable source of supply for the development of new biotechnological agents, as they permit fermentation-based production of natural products at large scales [1]. So far, marine biodiscovery efforts have mainly focused on taxa that are reputed for being prolific producers of bioactive natural products, such as actinomycetes and filamentous fungi (e.g., *Penicillium, Aspergillus* spp.), while yeasts have largely been neglected. Marine yeasts have adapted to survive in extreme environments including the deep-sea by developing higher osmotic and hydrostatic tolerance, as well as metabolic production of specific molecules and enzymes that pose promising potential for biotechnology [2,3].

*Rhodotorula* species are predominant basidiomycete yeasts in the marine environment [2,4], where they have been sourced from sea water, sediments, hydrothermal vents, but also as symbionts of marine invertebrates and seaweeds [4]. *Rhodotorula* is an oleaginous (lipid-accumulating) yeast that has been considered for the production of single cell oils [5]. The genus *Rhodotorula* shows high promise for industrial biotechnology for the production of biofuels, carotenoids, enzymes, and biosurfactants. Furthermore, species of this genus have been proposed as antagonistic yeasts for biocontrol of the phytopathogen *Botrytis cinerea* that causes *Botrytis* blight in fruits and beans [6,7].

In our ongoing efforts to explore the genetic, chemical, and biological diversity of marine microorganisms [8,9,10,11], we isolated six marine *Rhodotorula* strains from deep-sea sediments from several collection sites in the Mid-Atlantic Ridge, and screened their EtOAc extracts for biological activities against infectious human pathogens, plant pathogens, and cancer cell lines. Strain *R. mucilaginosa* 50-3-19/20B revealed very distinct media-dependent biological activity profiles when cultured on PDA and WSP-30 solid media. Herein, we employed computational genomics analysis and untargeted mass spectrometry-based metabolomics approaches to elucidate the differential biosynthetic output of the *R. mucilaginosa* strain 50-3-19/20B and to identify the compounds responsible for the distinct bioactivity profiles of the extracts. We implemented *de novo* genome assembly in combination with the “bioactive molecular networking” (BMN) concept [12], where chromatographic features of a fractionated extract are correlated with the bioactivity levels of the respective fractions to obtain a bioactivity score that is mapped out on a GNPS molecular network [13]. This approach enabled identification of glycolipids as anticancer constituents of the *R. mucilaginosa* 50-3-19/20B extract. These glycolipids were subsequently targeted for isolation and biological evaluation.

## 2. Results

To explore *Rhodotorula* species with bioactive and diverse metabolomes, we focused our isolation efforts on an underexplored deep-sea region of the Mid-Atlantic Ridge. An initial bioactivity screening against infectious human pathogens, plant pathogens and cancer cell lines of a small library of the six deep-sea *Rhodotorula* strains cultured on solid PDA (high carbon medium) and solid WSP-30 (medium with high salt content), highlighted one strain, *R. mucilaginosa* 50-3-19/20B, to exhibit interesting bioactivity and complex chemical UPLC-QToF-MS profiles (Table S1, Figure S1). Crude extracts of this strain exhibited anticancer activity only when cultured on PDA medium, while only in WSP-30 extracts antimicrobial activity was observed (Table S1). No inhibition of the phytopathogens, yeasts, and dermatophytes could be detected (Table S1); therefore, in this study, we focused only on positive bioactivity results, specifically the anticancer activity.

### 2.1. Culture Medium-Dependent Bioactivity Profiles and Metabolomics

We set out to examine the media-dependent metabolomic output of *R. mucilaginosa* 50-3-19/20B when grown on PDA and WSP-30 media to identify metabolites that may contribute to the differential bioactivity. Therefore, we up-scaled our cultivation efforts and grew the strain on 200 PDA and 200 WSP-30 agar plates. The extraction with EtOAc gave notably different yields; 3.5 g of extract was obtained from the PDA culture and only 0.7 g of extract was recovered from the WSP-30 culture. The two extracts were subjected to a modified Kupchan liquid–liquid partition scheme to yield *n*-hexane (K-hexane), dicholoromethane (K-DCM), and aqueous MeOH (K-MeOH) subextracts. The crude and the Kupchan subextracts were tested for their anticancer activity against two very common and deadly cancer types, using lung carcinoma cell line A549 and human breast cancer line MDA-MB-231). The antimicrobial potential was assessed against methicillin-resistant *Staphylococcus aureus* (MRSA) and *Enterococcus faecium*, whereas the general toxicity was assessed against a well-established non-cancerous human keratinocyte line HaCaT. The anticancer activity of *R. mucilaginosa* 50-3-19/20B could be tracked to the PDA-K-DCM subextract (Table 1), which exhibited 73% inhibition of the breast cancer line MDA-MB-231 at a test concentration of 100 µg/mL and 99% inhibitory activity against the lung carcinoma cell line A549. No toxicity against the non-cancerous human keratinocyte cell line HaCaT was detected in any of the extracts (Table 1). After fractionation, both K-hexane subextracts showed antimicrobial activity against both pathogens. The WSP-30-K-hexane extract showed specifically high inhibition values (90% and 98%) against MRSA and *E. faecium*, respectively, while the PDA-K-hexane extract inhibited MRSA at 74% (Table 1) at the test concentration of 100 µg/mL.

**Figure 1:**
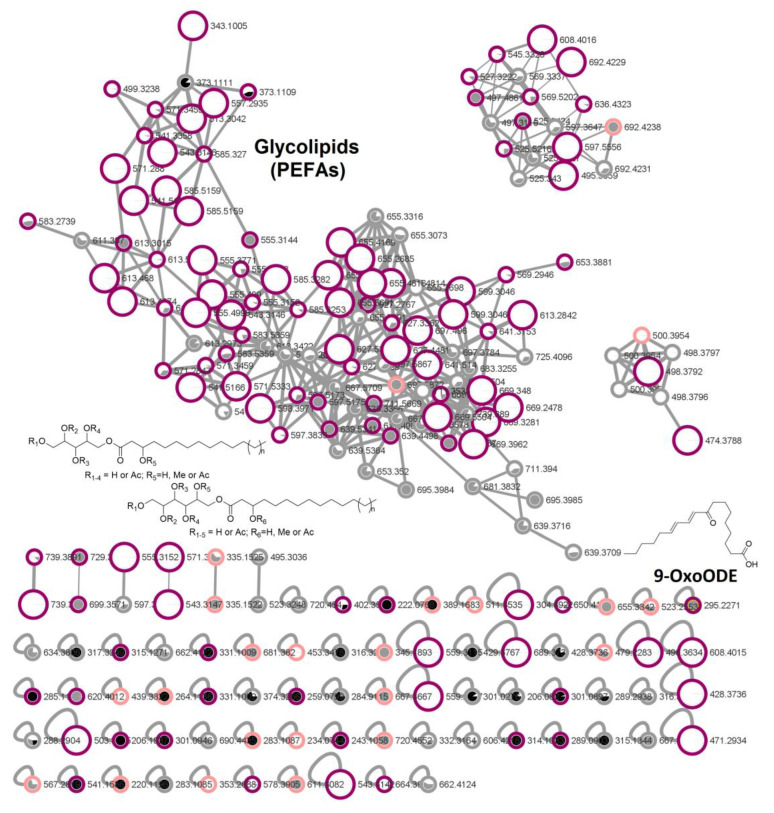
Comparative molecular network of Kupchan subextracts (MeOH: black, DCM: white, *n*-hexane: grey) of *R. mucilaginosa* 50-3-19/20B cultures on PDA (purple circled) and WSP-30 media (pink circled). Grey circled nodes are shared in extracts of both media. Nodes that only occur in the PDA-DCM subextract and thus are putatively associated with anticancer activity are presented as larger nodes.

Next, the Kupchan subextracts were profiled via UPLC-QToF-MS/MS and the data were used to construct a molecular network through the Global Natural Product Social (GNPS) online platform [13]. This open-access tool does not only facilitate rapid and automated dereplication of chemical profiles against a number of MS/MS fragment databases, but also serves as a useful comparative tool to investigate the chemical diversity of different samples. Therefore, we utilized molecular networking to compare the metabolomes of the differentially active subextracts to identify the compound classes that are responsible for their anticancer and/or antimicrobial activities. The molecular network (MN, Figure 1) consisted of three molecular families (clusters of more than two nodes). Dereplication workflows on GNPS and UNPD *in silico* database matching did not provide meaningful hits. Only one singleton with *m*/*z* 295.2271 [M + H]^+^ was putatively identified as an unsaturated fatty acid, 9-oxo-octadeca-10,12-dienoic acid (9-OxoODE, Figure 1 and Figure S3). Thus, we largely relied on manual dereplication against the Dictionary of Natural Products (http://dnp.chemnetbase.com), DEREP_NP [14], and SciFinder (https://scifinder.cas.org/) in order to annotate the MN. The largest molecular family in the MN could be identified as glycolipids via manual dereplication against relevant literature data [15,16,17]. These glycolipids belong to a class of polyol esters of fatty acids (PEFAs) with differential degrees of acetylation and have been proposed as biosurfactants [17]. The cluster consisted of 100 ions (Figure 1), which were predominantly expressed in PDA subextracts (K-DCM and K-hexane). 99 of the PEFA ions were detected in the PDA extracts (K-DCM and K-hexane) of which 68 ions were unique to PDA extracts (K-DCM, K-hexane, and K-MeOH) and 31 were shared between WSP-30 and PDA extracts (predominantly K-hexane subextracts); only one node (*m*/*z* 697.5872 [M + Na]^+^) in the glycolipid cluster originated solely from the WSP-30-K-hexane subextract. Only two low molecular weight ions of this cluster were detected in the K-MeOH subextracts (*m*/*z* 373.1109 [M + Na]^+^ and *m*/*z* 373.1111 [M + Na]^+^). In total, 36 nodes were unique to the PDA-K-DCM subextract and were thus annotated to potentially cause anticancer activity and are presented as larger nodes in Figure 1.

The two other main clusters could not be annotated and might constitute of new metabolites. Furthermore, based on the MN analysis, it did not become clearly apparent which compounds contributed to the antimicrobial activity. None of the main molecular families and only several singletons originated exclusively from the WSP-30-K-hexane subextract. It is likely that the antimicrobial activity is caused by synergy of multiple lipids. Hence, we decided to focus on the K-DCM subextract of the yeast cultured on PDA medium and its anticancer activity as the MN did not reveal any explicit antimicrobial target compounds in the K-hexane subextract.

### 2.2. Full Genome Sequencing and Analysis of Biosynthetic Gene Clusters

The *de novo* genome of marine *R. mucilaginosa* 50-3-19/20B was assembled. The genome assembly has a size of 20.02 Mb and an N50 of 498.8 kb (Figure S2). It possessed 2.14% of repetitive element contents (Figure S2). This genome size is comparable to other known *Rhodotorula* genomes (Table 2) as reported in previous studies [18,19]. Based on AntiSMASH 4.0 analysis, we discovered 19 biosynthetic gene clusters (BGCs) in total. Out of these, 15 BGCs are unknown, while two terpene BGCs and one NRPS, as well as one fatty acid based BGC were identified (Table 2). Numbers and types of BGCs in the different *Rhodotorula* genomes showed high inter-species similarities. All other *Rhodotorula* strains exhibited also one NRPS cluster; two terpene BGCs were specifically associated with *R. mucilaginosa* strains and *R. taiwanensis* MD1149 had three such BGCs. All *Rhodotorula* genomes, except for *Rhodotorula* sp. FNED7-22, contained a fatty acid BGC. We propose that this BGC is most likely involved in the synthesis of PEFAs that we detected through the metabolomics analysis and we thus further investigated this gene cluster.

**Figure 2:**
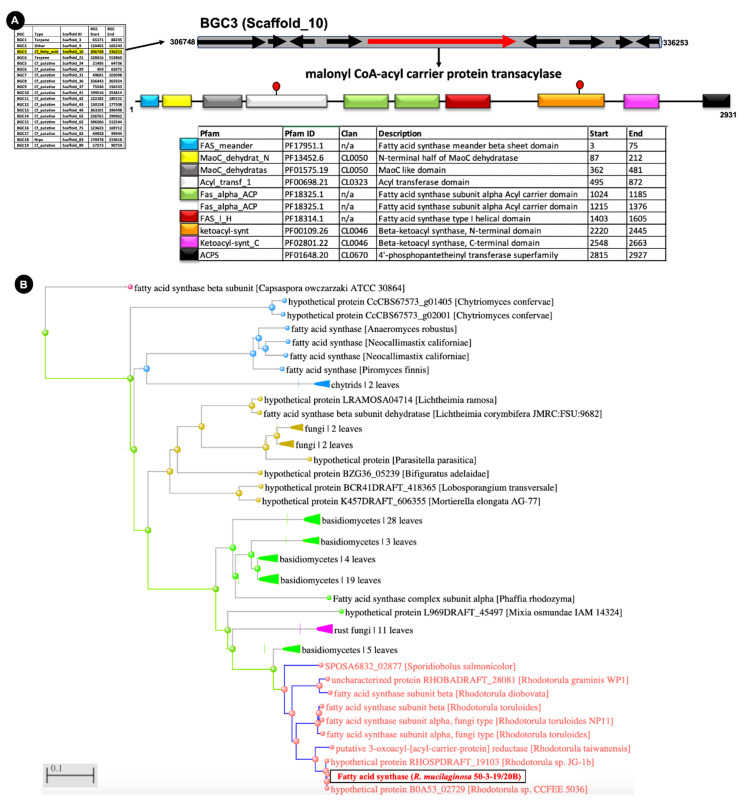
Overview of putative fatty acid producing biosynthetic gene cluster—BGC3. (**A**) Genomic and protein domain organization. (**B**) Phylogenetic distribution of fatty acid synthase/malonyl CoA-acyl carrier protein transacylase among fungi. The phylogenetic tree was constructed using the distance tree method and visualized using BLAST Tree View with the BLAST suite [22]. Leaves comprise fatty acid synthase/malonyl CoA-acyl carrier protein transacylase sequences from other closely related fungi and are left compressed for visualization purpose only.

We identified BGC3 (Figure 2) as the putative fatty acid producing cluster. BGC3 represents the only fatty acid producing gene cluster in the genome. Its core enzyme is a malonyl CoA-acyl carrier protein transacylase, which is a type of fatty acid synthase [20]. This synthase is a potential producer of fatty acids as it is characterized by the presence of a huge multifunctional enzyme complex with several protein domains (Figure 2A), like (i) the fatty acid synthase meander beta sheet domain (Pfam ID—PF17951.1); (ii) N-terminal half of MaoC dehydratase (PF13452.6); (iii) MaoC like domain (PF01575.19); (iv) acyl transferase domain (PF00698.21); (v) two fatty acid synthase subunit alpha acyl carrier domains (PF18325.1); (vi) fatty acid synthase type I helical domain (PF18314.1); (vii) beta-ketoacyl synthase, N-terminal domain (PF00109.26); (viii) beta-ketoacyl synthase, C-terminal domain (PF02801.22); and (ix) 4′-phosphopantetheinyl transferase superfamily domain (PF01648.20). These domains have previously been reported from several fatty acid producing core enzymes [21]. Table 2 depicts the presence of a single BGC (Cf_fatty_acid) in several *Rhodotorula* strains. Phylogenetic analysis further suggests that a single core enzyme is conserved across several *Rhodotorula* spp. (Figure 2B).

This is the only bona fide identified fatty acid producing biosynthetic gene cluster in different *Rhodotorula* strains (Table 2) suggesting that at least the fatty acid moiety of PEFA production is dependent on this BGC. However, the exact role of this BGC in the production of PEFA remains unclear.

### 2.3. Identification and Characterization of an Exo-Inulinase Enzyme

The knowledge on the PEFA biosynthetic pathway and its components is limited. A recent study suggested that an exo-inulinase enzyme holds key roles in the PEFA production via a mannitol biosynthesis pathway using the polysaccharide inulin as the carbon source [23]. Hence, we used the recently identified protein sequence of exo-inulinase enzyme from *R. paludigena* P4R5 [23] to detect enzymes in the marine *R. mucilaginosa* 50-3-19/20B genome aided by BLAST suite [22]. Herein, we report the existence of an exo-inulinase gene (as g1629.t1) and its genomic locus including its flanking genes deduced from the marine *R. mucilaginosa* 50-3-19/20B genome (Table S2).

**Figure 3:**
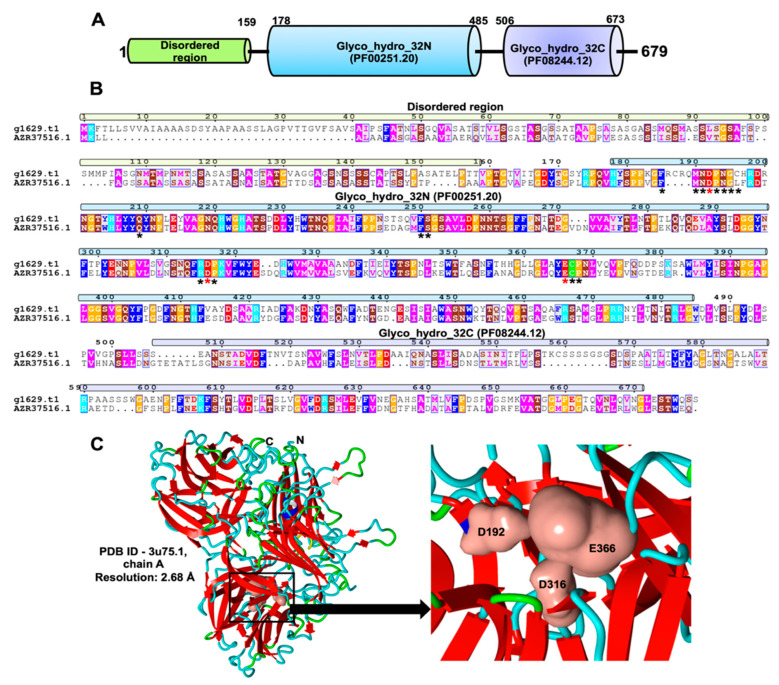
Characterization of exo-inulinase enzyme of marine *R. mucilaginosa*. (**A**) Protein Pfam domain organization of inulinase enzyme illustrated that it is a member of the GH32 family as it harbors two GH domains as Glyco_hydro_32N (PF00251.20) and Glyco_hydro_32C (PF08244.12) in 178-485 and 506-673 residues, respectively. It also possesses a disordered region in the N-terminal region (1-159 residues). (**B**) Protein sequence alignment of exo-inulinase enzymes of marine *R. mucilaginosa* (g1629.t1) and *R. paludigena* strain P4R5 (Genbank: AZR37516.1, [23]) illustrating the presence of conserved motifs of exo-inulinases as ^189^FMNDPNGC^189^, ^209^Q, ^250^FS^251^, ^315^RDP^317^, and ^366^ECP^368^ (marked by stars, numbering according to amino acid numbering of exo-inulinase enzyme of marine *R. mucilaginosa*). Residues forming the catalytic triad are ^192^D-^316^D-^366^E (marked by red stars). (**C**) Homology modeling of exo-inulinase enzyme of marine *R. mucilaginosa* with conserved catalytic triad as ^192^D-^316^D-^366^E. This model is based on the crystal structure of fructofuranosidase from *Schwanniomyces occidentalis* [26] with protein databank (PDB) ID as 3U75.1 (chain A and resolution 2.68 Å).

The corresponding peptide (g1629.t1) is 679 amino acids long and it has a molecular weight of 71.24 kDa. It harbors a disordered region of 159 amino acid in the N-terminus, plus two glycoside hydrolase family 32 (GH32) domains as Glyco_hydro_32N (Pfam ID—PF00251.20) and Glyco_hydro_32C (PF08244.12) mapped from 178–485 and 506–673 amino acids, respectively (Figure 3 and Table S2). Hence, this protein is a member of the glycoside hydrolase family 32 (GH32). We performed homology detection in NCBI and found several fungi to possess homologs of this putative exo-inulinase enzyme (Table S3). This result demonstrates that the exo-inulinase enzyme (g1629.t1) has orthologs in several fungi with annotation as either GH32 protein or *β*-fructofuranosidase (Table S3). This putative exo-inulinase harbors known conserved motifs of exo-inulinases [23,24,25] as ^189^FMNDPNGC^189^, ^209^Q, ^250^FS^251^, ^315^RDP^317^, and ^366^ECP^368^ (Figure 3, marked by stars, numbering according to amino acid numbering of exo-inulinase enzyme of marine *R. mucilaginosa*). Out of these residues, three amino acids ^192^D-^316^D-^366^E form the conserved catalytic triad (marked by red stars in Figure 3) with ^192^D serving as the nucleophile, ^316^D as a stabiliser of the transient state and ^366^E as the acid-base catalyst.

We further constructed a three-dimensional structural model of the enzyme using the crystal structure of fructofuranosidase from *Schwanniomyces occidentalis* [26] as template (PDB ID-3u75.1, chain A). This template shows 37.89% sequence identity with the identified exo-inulinase protein. It features the typical *β*-propeller with the conserved catalytic triad as ^192^D-^316^D-^366^E, which is known in several members of the GH32 protein family [23,26,27].

### 2.4. In-Depth Metabolome Analysis and Anticancer Activity

**Figure 4:**
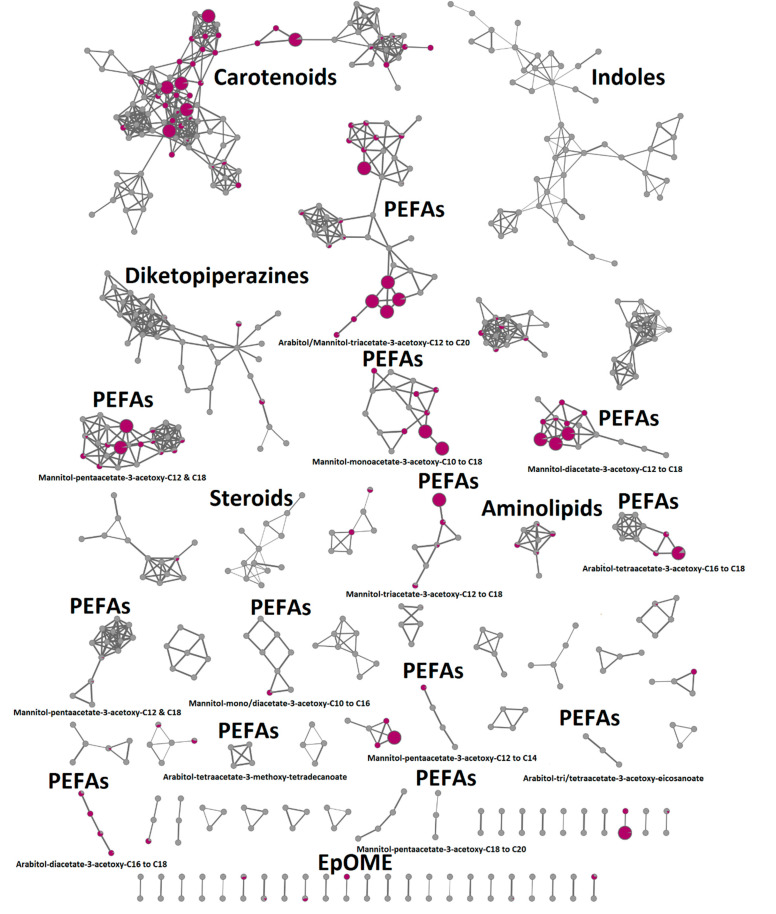
Bioactive molecular networking of the MPLC fractions obtained from the DCM extract of the deep-sea *R. mucilaginosa* 50-3-19/20B and their putative annotations. Nodes that originated from bioactive fractions are shown in purple and nodes showing strong correlation with anticancer activity (*r* > 0.5) are indicated by larger node size. Annotations are listed in Table S5.

In order to analyze the chemical constituents of the PDA-derived DCM extract of *R. mucilaginosa* 50-3-19/20B, we further fractionated the DCM subextract by C18-MPLC to obtain 31 fractions. Fractionation allows detection of minor ions that are often masked in crude and subextracts by UPLC-QToF-MS/MS. Therefore, we also performed anticancer screening (Table S4) and bioactive molecular networking (BMN) of the fractions (Figure 4). In BMN, features (in this case peak area) in the UPLC-QToF-MS/MS chromatograms are correlated with bioassay results to determine a bioactivity score. This score can be visualized through different node sizes in the MN (the larger the node, the higher its bioactivity score) and thus allows prediction of molecules or compound classes that contribute to the bioactivity.

The BMN revealed 39 different molecular clusters (Figure 4). The GNPS dereplication workflow revealed a diketopiperazine cluster, which included cyclo-(Leu-Phe) (*m*/*z* 261.1304 [M + H]^+^) that could be matched with high confidence against the GNPS library (Figure S4). This compound class, which is likely linked to the identified NRPS gene cluster (Section 2.2.), was not detected in our initial comparative MN analysis (Figure 1) and thus highlights the effectiveness of fractionation to enhance the detection of minor ions in extracts. A second cluster was found to comprise indole-containing compounds, such as DL-indole-3-lactic acid (*m*/*z* 206.0829 [M + H]^+^) (Figure 4 and Figure S5) and methyl-2-hydroxy-3-(1H-indol-2-yl)propanoate (*m*/*z* 220.0983 [M + H]^+^). These compounds clustered with other larger ions with molecular weights above 400 Da, these ions are potentially representing additional small peptidic compounds synthesized via the NRPS pathway.

A small cluster for steroids, which are truncated terpenes could also be putatively annotated (Figure 4 and Figure S6). Also associated with terpene biosynthesis are carotenoids that represented the largest cluster in the BMN. Molecular weights of several nodes were in agreement with those reported for carotenoids derived from microorganisms including *Rhodotorula* sp. [28]. These were tetrahydroxydihydrolycopene (*m*/*z* 603.5312 [M + H]^+^), dihydroxylycopene (*m*/*z* 571.6362 [M + H]^+^), and cryptoxanthin (*m*/*z* 553.5593 [M + H]^+^), however, the observed fragmentation patterns did not provide definite confirmation of their presence in the extract. Further GNPS annotations included C17-sphinganine, (*m*/*z* 288.2906 [M + H]^+^) belonging to the aminolipid cluster, (Figure 3 and Figure S7) and the fatty acid derivative 9,10-epoxy-12-octadecenoic acid (EpOMe, *m*/*z* 279.2330 [M-H_2_O + H]^+^) (Figure 4 and Figure S8).

As already observed in the comparative metabolomics studies of the Kupchan subextracts of the PDA and WSP-30 cultures, the metabolome of *R. mucilaginosa* 50-3-19/20B was dominated by acetylated polyol (sugar alcohol) esters of fatty acids (PEFAs), hence we established a rapid identification approach for this compound class. Because several PEFAs have the same molecular weight, MS/MS fragmentation data is highly valuable in differentiating the PEFAs. We first used a list of theoretical PEFA molecular weights that allowed immediate determination whether the PEFA contained a mannitol or arabitol polyol head group (Table S5). Second, the number of acetyloxy groups in the compound was established based on the number of MS/MS fragments that indicated the loss of 60 Da (one acetyloxy group). In PEFAs, acetylation can occur on the polyol unit as well as C-3 position of the fatty acid, thus the fragment produced by the natural loss of the fatty acid becomes highly useful in the identification of the PEFA. A table with the observed fragment ions for the residual polyols with different degrees of acetylation can be found in the supplementary information (Table S6). It should be considered that there are several isomers of moderately acetylated PEFAs and the position of the acetylation could not be established through this approach.

In the molecular network of the fractionated K-DCM subextract (Figure 4), the GNPS algorithm clustered the PEFAs depending on their degree of acetylation. We identified 49 different PEFA derivatives in *R. mucilaginosa* 50-3-19/20B containing either mannitol or arabitol as sugar alcohols (Table S7). Nine clusters had a mannitol head group while five contained arabitol (Figure 4).

Interestingly, the MN and manual MS/MS fragment analyses revealed that the PEFAs in *R. mucilaginosa* 50-3-19/20 had fatty acid chain lengths of C10, C12, C14, C16, C18, and C20 with either 3-hydroxy or 3-acetoxy substitutions, while prior reports of PEFAs derived from *Rhodotorula* spp. only reported chain lengths of C12, C14, C16, C18, and C20 [16,17,23,29]. Therefore, this is the first report of a PEFA containing decanoic acid (C10). Arabitol-tetraacetate-3-methoxy-tetradecanoate, a PEFA derivative bearing a 3-methoxy fatty acid was also detected.

The anticancer activity of the K-DCM subextract was tracked to the non-polar MPLC fractions (F21–F24, Table S4) containing complex mixtures of PEFAs and 22 nodes showed strong correlations (*r* > 0.5) with bioactivity (Figure 4). Therefore, we decided to purify PEFAs from the active *R. mucilaginosa* 50-3-19/20B MPLC fractions in order to determine their chemical structures and anticancer activities.

### 2.5. Compound Isolation and Bioactivity Testing

**Figure 5:**
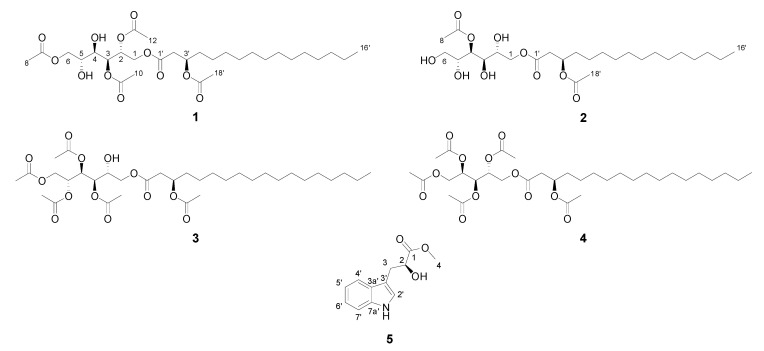
Isolated compounds (**1**–**5**) from *R. mucilaginosa* 50-3-19/20B.

The purification of PEFA glycolipids from *Rhodotorula*-derived extracts is highly challenging, as the yeast generally synthesizes a diverse range PEFAs (including different isomers) with high chemical resemblance and similar retention times. In the DCM subextract of *R. mucilaginosa* 50-3-19/20B, 99 nodes were attributed with the PEFA glycolipid cluster and all PEFAs eluted within 2.5 min in the UPLC-QToF-MS/MS chromatogram. Even after further MPLC fractionation, the most bioactive fractions, F22 and F24, were both highly complex in PEFAs. Based on visual inspection of the chromatographic data, F22 appeared to be better resolved, thus judged more promising for isolation of pure compounds. PEFAs lack a strong UV chromophore; therefore, fractionation was performed by time to afford four pure PEFA compounds, **1**–**4** (Figure 5) as clear oils in minor amounts. Their structure elucidations were based on extensive HR-MS/MS and NMR analyses.

**Figure 6:**
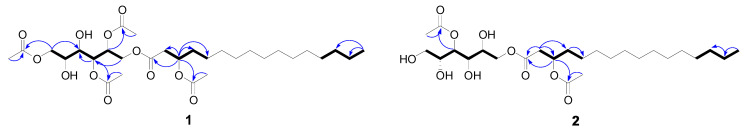
Key COSY (**bold**) and HMBC (**blue**) correlations within **1** and **2**.

HR-MS/MS data of compound **1** revealed a sodium adduct ion at *m*/*z* 627.3346 [M + Na]^+^ that corresponded to the molecular formula C_30_H_52_O_12_. Analysis of the HR-MS/MS spectrum revealed fragment ions at *m*/*z* 567.3137, *m*/*z* 507.2916 and *m*/*z* 447.2723 (Figure S9) arising from the natural loss of three acetyloxy groups (−60 Da each). The fragment *m*/*z* 313.0904 [M + Na]^+^ originated from mannitol-triacetate; thus compound **1** was tentatively identified as D-mannitol-triacetyloxy-3-acetyloxyhexadecanoate. 2D NMR data were used to confirm the planar structure of **1** (Figures S10–S15). The ^1^H NMR data (Table 3, Figure S10) together with the HSQC spectrum of **1** (Figure S11) revealed the presence of a terminal methyl group (H_3_-16′, *δ*_H_ 0.90, t, *J* = 6.9 Hz/*δ*_C_ 14.1) and four acetyl methyl groups resonating between *δ*_H_ 2.02–2.08 and *δ*_C_ 20.3–20.8, five oxygenated methine resonances H-2 (m, *δ*_H_ 3.79/*δ*_C_ 70.3), H-3 (m, *δ*_H_ 3.69/*δ*_C_ 72.7), H-4 (m, *δ*_H_ 3.48/*δ*_C_ 70.4), H-5 (m, *δ*_H_ 3.87/*δ*_C_ 70.0), H-3′ (m, *δ*_H_ 5.22/*δ*_C_ 71.8), and four methylene groups H_2_-1 (m, *δ*_H_ 3.63 and 3.80/*δ*_C_ 64.8), H_2_-6 (m, *δ*_H_ 4.16 and 4.37/*δ*_C_ 67.7), H_2_-2′ (m, *δ*_H_ 2.61 and 2.65/*δ*_C_ 39.8), H_2_-4′ (m, *δ*_H_ 1.61/*δ*_C_ 34.7) and highly overlapped methylene signals around *δ*_H_ 1.30 (m)/*δ*_C_ 23.5–32.9 characteristic of an aliphatic fatty acyl chain. Four additional acetyl carbonyl signals (*δ*_C_ 172.1, *δ*_C_ 172.3, *δ*_C_ 172.9, and *δ*_C_ 173.1) and another carbonyl resonating at *δ*_C_ 172.3 (C-1′) were extracted from the HMBC spectrum of **1** (Figure S13). Detailed analysis of the 2D NMR data (Table S8) revealed an isolated spin system belonging to the mannitol-triacetate. Key COSY correlations were observed between H_2_-6 (*δ*_H_ 4.16, and 4.37)/H-5 (*δ*_H_ 3.87), H-5/H-4 (*δ*_H_ 3.48), H-4/H-3 (*δ*_H_ 3.69) plus H-3 and H-2 (*δ*_H_ 3.79), which in turn correlated with the H_2_-1 methylene protons (*δ*_H_ 3.63 and 3.80) (Figure 6). Based on key HMBC cross-peaks (Figure 6, Table S8), the site of acetylations on the mannitol unit was assigned to C-2, C-3, and C-6. The remaining signals were attributed to the C16-fatty acyl chain. Position C-3′ was identified as the site of acetylation based on HMBC correlations of H-3′ (*δ*_H_ 5.22) with C-17′ (*δ*_C_ 172.3), C-1′ (*δ*_C_ 172.3), C-2′ (*δ*_C_ 39.8), C-4′ (*δ*_C_ 34.7), and C-5′ (*δ*_C_ 25.9).

The NOESY spectrum did not allow deduction of the stereochemical configurations within **1**. NMR chemical shifts in (*S*)- and (*R*)-3-hydroxy- and acetoxyhexadecanoic acids are identical and the two respective isomers only differ by their optical rotations [30]. This is the first time that a PEFA is chemically characterized by NMR and optical rotations, therefore, there is no data for comparison in order to unequivocally assign the stereochemistry of **1**. However, a previous study on the extracellular glycolipids of *R. babjevae* [15] used extensive derivatization experiments and chiral separation via GC-MS. This assigned an (*R*)-configuration at C-3′ (which is the site of *O*-acetyl substitution) of the fatty acid unit in PEFAs, while the polyol components were determined to be d-arabitol and d-mannitol. Additional studies by Wang et al. [23] also described that *Rhodotorula* exclusively synthesized d-mannitol. Our genomics analysis found the fatty acid synthase core enzyme is conserved across several *Rhodotorula* spp., thus it is biosynthetically reasonable to assume that the PEFAs in *R. mucilaginosa* 50-3-19/20B have the same configuration at all stereocenters. On this basis, we propose the structure of the new compound **1** as D-mannitol-2,3,6-triacetyloxy-(*R*)-3′-acetyloxyhexadecanoate.

Compound **2** was also purified as a colorless oil. The molecular formula C_26_H_48_O_10_ was assigned based on the sodium adduct ion observed in the HR-ESIMS spectrum of **2** at *m*/*z* 543.3141 [M + Na]^+^ (Figure S16). On the basis of the molecular networking analysis, this compound also belonged to the PEFA glycolipids. The fragment ions at *m*/*z* 483.2929 [M + Na]^+^ and *m*/*z* 423.27 [M + Na]^+^ indicated the presence of two acetyloxy groups and the ion at *m*/*z* 229.0757 [M + Na]^+^ was indicative of a monoacetylated mannitol. The NMR data of **2** was almost identical to that of compound **1** (Table 3, Table S9, Figures S17–S21). COSY and HMBC correlations (Table S9, Figure 6, Figures S19 and S21) revealed that **2** also contained an acetyloxy group at position 3′ of the C16-fatty acyl chain. Further, the HMBC correlation from H-4 (*δ*_H_ 3.47) to a carbonyl resonance at *δ*_C_ 172.9 (C-7) (Figure 6, Figure S21) indicated the final acetyl substitution to be at the C-4 position of the mannitol unit. Hence, the new compound **2** was determined as D-mannitol-4-monoacetyloxy-(*R*)-3′-acetyloxyhexadecanoate.

The HR-ESIMS spectrum of **3** (Figure S22) revealed a sodium adduct ion at *m*/*z* 697.3789 [M + Na]^+^ that corresponded to the molecular formula C_34_H_58_O_13_. HR-MS/MS fragment ions at *m*/*z* 637.3586, *m*/*z* 577.3372, *m*/*z* 517.3150, and *m*/*z* 457.2921 indicated the presence of four acetyloxy groups while the ions at *m*/*z* 335.1011 and *m*/*z* 295.0796 (difference of 60 Da) pointed to an additional acetyloxy group in **3**. The presence of tetraacetylated mannitol was identified based on the residual ion at *m*/*z* 355.1011 [M + Na]^+^ originating from the loss of acetyloxyoctadecanoic acid. Due to poorly resolved NMR data for **3** the exact acetylation sites of the polyol could not be assigned. As optical rotations of **3** indicated the same configuration as in compounds **1** and **2**, we proposed compound **3** as a D-mannitol-tetraacetyloxy-(*R*)-3′-acetyloxyoctadecanoate derivative. The structure of **3** shown in Figure 5 is only a tentative structure and needs to be confirmed in the future.

The last PEFA glycolipid **4** with the molecular formula C_33_H_56_O_12_ (*m*/*z* 667.3669 [M + Na]^+^) was also isolated from the bioactive MPLC fraction F22. Similar to PEFA **3**, the ^1^H NMR spectrum of **4** was poorly resolved; thus, we solely used HR-MS/MS data for structural analysis. Analysis of the HR-MS/MS spectrum showed fragment ions at *m*/*z* 607.34763, *m*/*z* 547.3257, and *m*/*z* 487.2971 (Figure S23) arising from the natural loss of three acetyloxy groups. Fragment ions at *m*/*z* 525.3370 and 465.3260 as well as *m*/*z* 505.3015 and 445.5472 indicated the presence of two more acetyl functions. The ion at *m*/*z* 325.0901 [M + Na]^+^ originated from tetraacetylated arabitol. Thus, the chemical structure of **4** was determined as D-arabitol-2,3,4,5-tetraacetyloxy-(*R*)-3′-acetyloxyoctadecanoate. This compound was previously identified in the crude extract of *R. babjevae* by LC-MS as well as GC-MS with various derivatization methods [15].

The remaining bioactive MPLC fractions contained complex mixtures of the PEFA type glycolipids that could not be separated by different chromatography columns and solvent gradients.

The molecular network indicated that *R. mucilaginosa* 50-3-19/20B produced indole alkaloids (Figure 4), which can be attributed to the detected NRPS cluster by AntiSMASH analysis. These molecules occurred in earlier eluting brown-colored fractions (F1-F8), which were inactive against the cancer cell line MDA-MB-231. To confirm the presence of indole type compounds, we performed rapid isolation of the major compound (**5**) in F2. HR-MS/MS data of **5** (Figure S24) revealed a pseudomolecular ion at *m*/*z* 220.0983 [M + H]^+^ C_12_H_14_NO_3_ (calculated for 220.0974), this molecular formula corresponded to methyl-2-hydroxy-3-(1H-indol-2-yl)propanoate (Figure 5). 1D and 2D NMR data were used to confirm the structure of **5** (Table S10, Figures S25–S30), which was previously isolated by Cimmino et al. from *Diaporthella cryptica*, a fungus derived from a hazelnut branch [31]. ^1^H NMR and specific rotation data of **5** ($[\alpha {]}_{D}^{20}$ −1.7, *c* 0.15, CHCl_3_) were in agreement with those reported in the literature ($[\alpha {]}_{D}^{20}$ −2.6, *c* 0.58, CHCl_3_) therefore we conclude that **5** is in (*S*)-(–)-form.

Compounds **1**–**5** were tested in vitro for their cytotoxic activity against the breast cancer cell line MDA-MB-231 and the non-cancerous human keratinocyte cell line HaCaT, however, in their pure form compounds **1**–**5** were devoid of any inhibitory activity at the test concentration of 100 µg/mL.

## 3. Discussion

### 3.1. Biosynthetic Potential of the Deep-Sea R. Mucilaginosa 50-3-19/20B

Even though *Rhodotorula* spp. are ubiquitous in the marine environment, their role in the marine realm is still poorly understood [32]. These yeasts are believed to contribute to decomposition of organic matter, nutrient-recycling, biodegradation of oils, and parasitism of marine animals [32]. Specifically, high numbers of *Rhodotorula* spp. have been reported from deep-sea sediments [2]. In this study, we combined full genome sequencing with metabolomics to investigate the metabolic potential of a *R. mucilaginosa* 50-3-19/20B strain isolated from deep-sea sediment samples collected from the Mid Atlantic Ridge (−3602.7 m). Genomics provides insight about the metabolic potential of a microorganism, while metabolomics identifies the end products of the gene expression. *Rhodotorula* spp., like most yeasts, have considerably low biosynthetic potential in terms of complex bioactive secondary metabolites compared to other fungal genera, such as *Penicillium* or *Aspergillus*. The Dictionary of Natural Products includes only 239 compounds for yeasts in general and of those only 22 are reported for the genus *Rhodotorula.* As shown in Figure S31, the genus *Rhodotorula* has been reported to contain sugars, lipids, carotenoids [33], two polyketides [34] and small peptides [35]. Here, through full genome sequencing of *R. mucilaginosa* 50-3-19/20B, we detected BGCs belonging to terpenes (two clusters), NRPS (one BGC) and fatty acids (one BGC). The metabolomics analysis of the strain allowed us to link metabolites with the identified BGCs. Accordingly, one terpene cluster could be linked to carotenoids and the second BGC is likely to belong to sterols. Indole-derivatives are putatively synthesized via NPRS, while polyol ester fatty acids (PEFAs) are the modified end products of the fatty acid biosynthetic gene cluster.

PEFA glycolipid production is the hallmark of *Rhodotorula* biology [36]. Several strains are known to produce PEFAs and this knowledge dates back to initial studies in the 1960s by Stodola et al. [37]. *Rhodotorula* spp. have been extensively explored for biofuel production [36]. Still details of the PEFA producing biosynthetic pathway are largely unknown. In the current study, we have deduced BGCs from various *Rhodotorula* strains using comparative BGCs genomics (Table 2)*. Rhodotorula* strains lack polyketide synthase (*pks*) genes in their genomes (Table 2). This finding is consistent with the current state of knowledge on other basidiomycete yeasts [38] including *R. babjevae* UCDFST 04-877 and *R. aff. paludigena* UCDFST 81-84 [36]. Detection of fatty acid producing BGC alone is not sufficient to explain biosynthetic pathways of all types of fatty acid production potentials of a given yeast. This complicates a simple direct inference of a biosynthetic pathway from the BGCs analysis.

**Figure 7:**
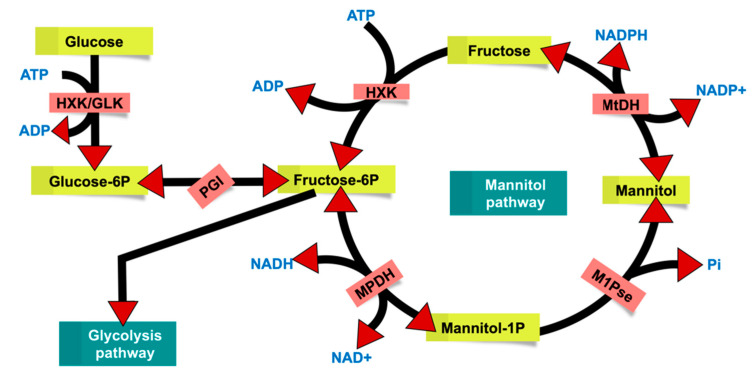
Overview of fungal mannitol pathway as proposed by Hult and Gatenbeck [39]. HXK—hexokinase (EC 2.7.1.1), GLK—glucose kinase (EC 2.7.1.2), MtDH—mannitol dehydrogenase (EC 1.1.1.138), MPDH—mannitol-1-phosphate dehydrogenase (EC 1.1.1.17), M1Pse—Mannitol-1-phosphate phosphatase (EC 3.1.3.22), and PGI—glucose-6-phosphate isomerase (EC 5.3.1.9).

This study has identified a potential BGC for fatty acid synthesis in the marine *R. mucilaginosa* strain (BGC3), which is likely to be also present in several other *Rhodotorula* strains as evident from the comparative analyses, which predicted a single conserved BGC. Our phylogenetic analysis confirmed that this cluster is indeed conserved across several *Rhodotorula* species (Figure 2B). This gene cluster is potentially responsible for PEFA production as it is a large multifunctional enzyme complex with several protein domains (Figure 2A) mapped to different locations in the full-length protein. This core enzyme can be utilized for both PEFA and PUFA production [21]. However, this result alone does not provide a detailed picture of the entire biosynthetic pathway and its contributing components. Several *Rhotodorula* strains are capable PEFA producers indicating a potentially conserved mode of PEFA production [36]. Recent studies have identified the exo-inulinase enzyme as key for PEFA production via the fungal mannitol biosynthesis pathway [23], which was originally proposed by Hult and Gatenbeck [39] (Figure 7).

We have identified an exo-inulinase enzyme genomic locus (g1629.t1) in the *R. mucilaginosa* 50-3-19/20B genome. This enzyme uses inulin as carbon source [23] for PEFA production. It is a member of glycoside hydrolase family 32 (GH32) with a size of 71.24 kDa and harbors conserved protein sequence motifs with the catalytic triad (^192^D-^316^D-^366^E). Previous studies reported this enzyme also in other fungi identifying the well-known sequence signatures and catalytic triad of exo-inulinases [23,24,25]. In conclusion, our results suggest that the fungal mannitol pathway and exo-inulinase enzyme hold key roles for PEFA production in *Rhodotorula* strains.

### 3.2. Glycolipid Production in Rhodotorula and Potential Biotechnological Application

PEFAs are extracellular lipids that are secreted into the media and specifically enhanced on potato dextrose agar (PDA) [40] that is rich in both complex and simple carbohydrates. This study could confirm this observation. *R. mucilaginosa* 50-3-19/20B gave five-times higher extract yields and a much greater diversity of PEFAs when cultured on PDA medium versus the low carbon medium WSP-30. PEFA production appears to be almost exclusive to the genus *Rhodotorula* [36] being reported from *R. paludigena* P4R5, *R. taiwanensis* MD1149 [2], *R. glutinis*, *R. toruloides*, *R. graminis*, *R. babjevae* [3,4], *R. diobovata*, *R. kratochvilovae*, *R. paludigena*, and *R.* aff. *paludigena* [41]. Production of extracellular lipids in *R. mucilaginosa* has been described in 1963, yet the chemical structures of these lipids had not be determined; thus, this study provides the first confirmed report of *R. mucilaginosa* as a PEFA producer.

The ecological role of glycolipids such as PEFAs in yeasts can only be speculated. It has for instance been proposed that glycolipids function as an external carbon storage [42]. As the majority of *Rhodotorula* species have been isolated from terrestrial sources, a prominent theory states that glycolipids aid in the modification of leaf surfaces to make them more permeable for the uptake of hydrophobic long carbon chain waxes present in leaf cuticles [41]. Others have suggested that yeast glycolipids are an antimicrobial defense [43]; though, in our study, the fractions containing the PEFA glycolipids did not show potent activities against the tested human pathogens, MRSA and *E. faecium* strains. Yeasts thrive in environments with high osmotic pressure such as the deep-sea, glycolipids may therefore also function in osmoprotection.

Liquid chromatography-tandem mass spectrometry is a successful tool for the analysis and identification of lipids [15]. On the basis of UPLC-QToF-MS/MS data, we were able to annotate 49 different PEFAs in *R. mucilaginosa* 50-3-19/20B. The majority of PEFA glycolipids in *R. mucilaginosa* 50-3-19/20B had mannitol as the backbone of the polar head group and hydrophobic C18 and C16 fatty acyl chains. Fewer PEFAs had chain lengths of C12, C14, and C20 and we present the first account of PEFAs with a C10 fatty acid chain. Seven PEFAs have been reported as the most abundant (C_29_H_50_O_11_, C_32_H_54_O_13_, C_34_H_56_O_14_, C_31_H_52_O_12_, C_34_H_58_O_13_, C_31_H_54_O_11_, and C_33_H_56_O_12_) [36], all of which could also be detected in *R. mucilaginosa* 50-3-19/20B. These seven PEFAs have either fully acetylated polyol head groups or maximum one free hydroxyl group. In *R. mucilaginosa* 50-3-19/20B, we further detected many more uncommon PEFAs with fewer degrees of acetylation, which are likely to have more effective and higher surfactant activity compared to hyper-acetylated PEFAs [17]. In this work, we isolated the two new PEFAs **1** and **2** with three and one degrees of acetylation on the mannitol unit, respectively. Using 2D NMR we were able to assign the sites of acetylation on these compounds and this is the first report of NMR data for PEFAs from *Rhodotorula*.

The BMN approach indicated that the anticancer activity, which was detected in several MPLC fractions, was linked to PEFAs. Anticancer activities of a mixture of PEFAs against four different cancer cell lines were previously described by Guerfali et al. with IC_50_ values of about 30 µg/mL, where after longer exposure of 48 h to the extract the IC_50_ values were further reduced [29]. In this study, we also detected strong anticancer activity of the PEFA-containing subextract and fractions against the lung carcinoma (A549) and the breast cancer (MDA-MB-231) cell lines. Here, purified PEFAs were tested for their cytotoxic activity for the first time; however, they exhibited no inhibitory activity against the tested cell lines (test concentration 100 µg/mL). In natural product research, despite the historical success of bioassay-guided fractionation, loss of activity during fractionation is very common since the initially observed activities are resulting from mixtures of compounds with synergistic, additive, or antagonistic activity [44]. Diseases are often of multi-factorial nature, thus mixtures of natural products offer an important resource for drug development [44]. Originally, the inhibition rate of the *R. mucilaginosa* 50-3-19/20B DCM subextract against the breast cancer cell line MDA-MB-231 was 73% at a test concentration of 100 µg/mL. After MPLC fractionation, even stronger anticancer activity (MDA-MB-231 cell line) was tracked to fractions F22 and F24 (94% and 97%, respectively), while pure compounds were inactive. This indicates that the observed activity in the subextract and MPLC fractions was of synergistic nature. Studies on bioactive biosurfactant-glycolipids proposed the primary mode of action of such compounds to be membrane lipid perturbation [45,46] that can lead to cell lysis or increased cell permeability, which could also facilitate the entry of other bioactive metabolites [47]. Since the PEFA-containing extracts did not show any cytotoxicity against the HaCaT cell line, it could be of great interest to investigate combinatorial treatments with other anticancer agents and whether addition of PEFAs could increase the bioactivity.

Previous studies have suggested different applications for PEFAs in biotechnology. It has been proposed that PEFAs, due to their physiochemical properties, could be used as biosurfactants in cosmetics, food, pharmaceuticals, detergents, and paints [36,48]. PEFA mixtures showed antifoam activity and reduced surface tension [36]. PEFAs are biodegradable, thus less toxic to the environment and hence preferred over synthetic surfactants [17]. Additionally, red oleaginous yeasts are economically suitable for scale-up fermentation, which is not very labor-intensive and does not require vast land resources compared to plant-based biooil production and is independent of seasons or the climate [29]. *Rhodotorula* spp. are known to co-produce extracellular PEFAs with other (intracelluar) lipids, such as triacylglycerols [23]. Several *Rhodotorula* species are the source of various carotenoids [49], making them highly interesting for biotechnology, because fermentation of a single culture will yield more than one natural product class. Secretion of higher value lipids is more desirable for economic purposes, making the isolated deep-sea *R. mucilaginosa* 50-3-19/20B a suitable candidate for further culture-optimization studies and potential commercialization, since the PEFA production was very high in this strain. Guerfali et al. suggested the use of PEFAs as therapeutic agents due to their anticancer activity [29]. Here, we confirmed anticancer activity of PEFAs in mixed fractions, however, the pure compounds were devoid of activity.

In conclusion, the present untargeted metabolomics investigation indicated that *R. mucilaginosa* 50-3-19/20B contained a breadth of metabolites, including synergistically active PEFAs, specifically when cultured on a PDA medium. Full genome sequencing provided new insights into PEFA biosynthesis through the identification of the fatty acid producing BGC3, which is conserved in many *Rhodotorula* species. Also, the genomic locus for an exo-inulinase enzyme (g1629.t1), which plays an instrumental role in the PEFA production via the mannitol biosynthesis pathway was identified. This study demonstrated that *Rhodotorula* is a promising yeast genus for biotechnological applications in the production of biooils and biosurfactants. This is the first study using (bioactive) molecular networking based metabolomics strategy to analyze the in-depth metabolome of a *Rhodotrula* isolate and the first study isolating and chemically characterizing PEFAs by 2D NMR and HRMS/MS methods.

## 4. Materials and Methods

### 4.1. General Procedures

LC-MS/MS data were acquired on an Acquity UPLC I-Class System coupled to a Xevo G2-XS QToF Mass Spectrometer (Waters, Milford, MA, USA) with an Acquity UPLC HSS T3 column (High Strength Silica C18, 1.8 µm, 2.1 × 100 mm, Waters, Milford, MA, USA). NMR spectra were recorded on a Bruker AV 600 spectrometer (600 and 150 MHz for ^1^H and ^13^C NMR, respectively, Bruker^®^, Billerica, MA, USA) The residual solvent signal was used as internal references: *δ*_H_ 3.31/*δ*_C_ 49.0 (MeOD). For MPLC a Büchi Glass column (26 × 230 mm, borosilicate 3.3, Büchi Labortechnik AG, Flawil, Switzerland) filled with C18 material (Sepra C18-E; 50 μm, 65 Å, Phenomenex, Torrance, CA, USA) was used. Preparative HPLC separation was performed on a Gemini-NX C18 column (50 × 100 mm, Phenomenex, Torrance, CA, USA) attached to a LaPrep system consisting of a P110 pump (VWR International, Allison Park, PA, USA) with a Dynamic Mixing Chamber (Knauer, Berlin, Germany), P311 UV/VIS and Labocol Vario-2000 Fraction Collector (Labomatic, Allschwil, Switzerland). HPLC separations were achieved on a VWR Hitachi Chromaster HPLC system (VWR International, Allison Park, PA, USA) consisting of a 5430 diode array detector (VWR International, Allison Park, PA, USA), a 5310 column oven, a 5260 autosampler, and a 5110 pump combined in parallel with a VWR Evaporative Light Scattering Detector (ELSD 90). UPLC solvents were purchased from Biosolve (Valkenswaard, The Netherlands). The water used was MilliQ-water filtered in-house on Arium^®^ Water Purification Systems (Sartorius, Göttingen, Germany). EtOAc, *n*-hexane, DCM, MeOH, and ACN were purchased from AppliChem GmbH (Hannover, Germany). NMR solvents were from Roth GmbH (Karlsruhe, Germany). Specific rotations were measured on a Jasco P-2000 polarimeter (Jasco, Pfungstadt, Germany).

### 4.2. Isolation of Deep-Sea Rhodotorula Species and Cultivation

The six *Rhodotorula* spp. were isolated from deep-sea sediments of the Mid Atlantic Ridge during a 2016 research cruise of the vessel Maria S. Merian (MSM58) (Table 4). These samples were collected using a multi corer or large box corer and were provided by colleagues from the Geoscience Department of University Kiel. Sediment samples consisted of sandy material and 1–3 mL of NaCl solution (30 g NaCl/L) was added to allow to pipet the sediment material. WSP-30 agar (50.5 mM d-(+)-glucose monohydrate, peptone from soy bean 5 g/L, malt extract 3 g/L, yeast extract 3 g/L, 513 mM NaCl, agar-agar 20 g/L), with 20 mg/mL streptomycin and 20 mg/mL ampicillin to reduce bacterial growth was prepared and poured into plates of about 8 cm diameter. On each plate, five 50 µL drops of sediment were added. Plates were incubated for up to two weeks at 20 °C. Fungal yeast-like colonies appeared after 7–10 days and were isolated. Pure cultures were established on WSP-30 without antibiotics.

Initial small scale profiling (10 agar plates) of the six *Rhodotorula* isolates, as well as scaled-up cultivation of *R. mucilaginosa* 50-3-19/20B (200 agar plates) were fermented on PDA (potato infusion extract 4 g/L, glucose monohydrate 20 g/L and noble agar 15 g/L) and WSP-30 (glucose monohydrate 10 g/L, peptone from soymeal 5 g/L, malt extract 3 g/L, yeast extract 3 g/L, NaCl 30 g/L, and noble agar 15 g/L). Culture plates were inoculated at 22 °C in the dark for 2 weeks.

### 4.3. Full Genome Sequencing and Assembly

Whole genome DNA samples of strain *R. mucilaginosa* 50-3-19/20B were prepared by following a modification of previously published methods [50,51,52]. Mycelium was frozen in liquid nitrogen, pulverized, and incubated in equal volumes of lysis buffer (10 mM Tris-HCl, 1 mM EDTA, 100 mM NaCl, 2% SDS, pH 8.0). After centrifugation, the supernatant was treated with RNase, and afterwards with an equal volume phenol/chloroform (1:1). Short-read DNA sequencing was performed by Illumina HiSeq™ 2000 at BGI Genomics (Shenzen, China) using 20 µg of genomic DNA. We constructed a *de novo* genome assembly of Illumina HiSeq™ 2000 reads using the CLCBio Genomic workbench 12 [53]. We detected repetitive elements from this genome assembly using the library of all fungal repetitive elements from the Repbase database through RepeatMasker version 4.0.5 (http://www.repeatmasker.org). We carried out gene prediction using Augustus 3.0 [54] Genome annotation was performed using BLAST homology suite [22] coupled by OMICSBOX [55] and PANNZER 2.0 [56]. 

### 4.4. Deducing Biosynthetic Gene Clusters (BGCs)

We analyzed the assembled marine *R. mucilaginosa* 50-3-19/20B genome for putative BGC clusters by antiSMASH4 [57], using the standard parameters. We also analyzed BGCs of published *Rhodotorula* genomes as summarized in Table 2. We identified the functional domains in the core biosynthetic genes as previously described [58], using a combination of tools namely antiSMASH4 [59], NCBI Conserved Domain Database [60], InterPro [61] and the PKS/NRPS Analysis Web-site [62]. We performed several rounds of specialized BLAST [22] for scanning gene homologs and/or corresponding proteins at an *e*-value < 1 × 10^−10^. We deduced Pfam domains using HMMER [63].

### 4.5. Phylogenetic Analyses

Phylogenetic analyses of the core protein of fatty-acid producing BGC using annotated proteins were performed using distance tree method within BLAST suite [22]. The resulting phylogenetic tree was visualized using BLAST Tree View encompassed in the BLAST suite [22].

### 4.6. Detection and Characterization of Inulinase Enzyme

We detected the gene for the inulinase enzyme using BLAST suite [22] using inulinase protein (Genbank: AZR37516.1) of *R. paludigena* strain P4R5 as the BLAST query sequence. We characterized protein domains of the detected inulinase enzyme using HMMER [58]. Inulinase protein sequences were aligned using MUSCLE alignment suite [64] and the resulting protein alignment was visualized by ESPrint3.0 tool [65]. The homology model of inulinase enzyme was constructed using SWISS-MODEL [66]. For homology model building, we used the crystal structure of fructofuranosidase from *Schwanniomyces occidentalis* [26] as the structural template with protein databank (PDB) ID as 3U75 with A chain.

### 4.7. Extraction, Fractionation, and Isolation

The culture plates were extracted with EtOAc (approximately 40 mL/petri dish), the agar pieces were homogenized with an Ultra-Turrax (IKA^®^-Werke GmbH & Co. KG, Staufen, Germany) at 13,000 rpm for 30 s and left on a rotary shaker at 120 rpm and room temperature overnight. The following day, the EtOAc phase was decanted into a 2 L separation funnel, the extract was partitioned with an equal volume of milli-Q H_2_O in order to remove salts and polar media components from the extract. The EtOAc phase was evaporated until dryness under reduced pressure. The extraction of the culture homogenate was repeated with a second round of EtOAc at 15 min sonication and again contra-extracted with milli-Q H_2_O. The two extraction rounds were combined and dried to yield 3.5 g and 0.7 g of extract of PDA and WSP-30 respectively for *R. mucilaginosa* 50-3-19/20B. The extracts were redissolved in MeOH, 0.2 µm PTFE-filtered (Carl Roth, Germany), dried under nitrogen and kept in the dark at 4 °C.

The EtOAc extract was partitioned using a modified Kupchan protocol [67] to yield *n*-hexane, DCM and aqueous MeOH subextracts. All subextracts were tested for their bioactivities against MRSA and *E. faecium*, as well as the cancer cell lines M231, A549 and the non-cancerous cell line HaCaT. The anticancer activity was concentrated in the K-DCM subextract (2.62 g), which was further fractionated via C18-MPLC on a Büchi Glass column (26 × 230 mm) connected to a LaPrep P110 LPG pump. A gradient solvent system from 10% aqueous MeOH to 100% MeOH over 1 h collecting fractions every 2 min was used. This yielded a total of 31 fractions (F1–F31, which were tested for anticancer activity and analyzed by UPLC-QToF-MS/MS. The MPLC fraction F22 (299 mg) that retained the anticancer activity was further fractionated via preparative HPLC (column: Luna 5 µm C18 (25 × 250 mm, Phenomenex; solvent gradient: 13:87 H_2_O/ACN(+0.1%FA) to 3:97 H_2_O/ACN(+0.1%FA) over 30 min, isocratic to 23 min, 25 min 100% ACN(+0.1%FA), isocratic to 30 min; flow 20 mL/min) to yield another 30 subfractions. Subfraction 14 (14 mg) was subjected to semi-preparative RP–HPLC equipped with an Onyx (100 × 10 mm, Phenomenex) C18 monolithic column (gradient of H_2_O/ACN +0.1%FA from 9:1 to 1:1 in 30 min, flow 2.5 mL/min, column oven 40 °C) to yield 28 fractions of which fraction 3 contained compound **1** (0.3 mg) in a pure state. Subfractions 15–17 were combined (66 mg) and fractionated via preparative HPLC using a Luna 5 µm C18 column (25 × 250 mm, Phenomenex) (isocratic gradient of 14:86 H_2_O/ACN +0.1%FA; flow 15 mL/min in 60 min), starting fraction collection from 15 min in 30 s intervals to afford compound **2** (0.2 mg, in fraction 13), compound **3** (0.6 mg, in fraction 66) and compound **4** (0.4 mg, in fraction 72). MPLC fraction F2 (31 mg) was fractionated via semi-preparative RP–HPLC using the same Onyx (100 × 10 mm, Phenomenex) C18 monolithic column (gradient of H_2_O/ACN +0.1%FA from 9:1 to 1:1 in 30 min, flow 2.5 mL/min, column oven 40 °C) to yield the pure compound **5** (t_R_ 16.9 min, 0.8 mg).

d-Mannitol-2,3,6-triacetyloxy-(*R*)-3′-acetyloxyhexadecanoate (**1**): colorless oil; $[\alpha {]}_{D}^{20}$ −14.3 (*c* 0.05, MeOH); ^1^H NMR (MeOD, 600 MHz) and ^13^C NMR (MeOD, 150 MHz), Table 3; (+)-HR-ESIMS found *m*/*z* 627.3346 [M + Na]^+^ C_30_H_52_O_12_Na, calculated for 627.3351; deposited in the GNPS spectral library, https://gnps.ucsd.edu/ProteoSAFe/gnpslibraryspectrum.jsp?SpectrumID=CCMSLIB00005725521#%7B%7D

D-Mannitol-4-monoacetyloxy-(*R*)-3′-acetyloxyhexadecanoate (**2**): colorless oil; $[\alpha {]}_{D}^{20}$ −15.5 (*c* 0.05, MeOH); ^1^H NMR (MeOD, 600 MHz) and ^13^C NMR (MeOD, 150 MHz), Table 3; (+)-HR-ESIMS found *m*/*z* 543.3141 [M + Na]^+^ C_26_H_48_O_10_Na, calculated for 543.3140; deposited in the GNPS spectral library, https://gnps.ucsd.edu/ProteoSAFe/gnpslibraryspectrum.jsp?SpectrumID=CCMSLIB00005725522#%7B%7D

d-Mannitol-tetraacetyloxy-(*R*)-3′-acetyloxyoctadecanoate derivative (**3**): colorless oil; $[\alpha {]}_{D}^{20}$ −3.7 (*c* 0.15, MeOH); (+)-HR-ESIMS found *m*/*z* 697.3789 [M + Na]^+^ C_34_H_58_O_13_Na, calculated for 697.3770; deposited in the GNPS spectral library, https://gnps.ucsd.edu/ProteoSAFe/gnpslibraryspectrum.jsp?SpectrumID=CCMSLIB00005725523#%7B%7D

d-Arabitol-2,3,4,5-tetraacetyloxy-(*R*)-3′-acetyloxyoctadecanoate (**4**): colorless oil; $[\alpha {]}_{D}^{20}$ −5.2 (*c* 0.45, MeOH); (+)-HR-ESIMS found *m*/*z* 667.3669 [M + Na]^+^ C_33_H_56_O_12_Na, calculated for 667.3664; deposited in the GNPS spectral library, https://gnps.ucsd.edu/ProteoSAFe/gnpslibraryspectrum.jsp?SpectrumID=CCMSLIB00005725524#%7B%7D

Methyl-2-hydroxy-3-(1H-indol-2-yl)propanoate (**5**): yellow solid; $[\alpha {]}_{D}^{20}$ −1.7 (*c* 0.15, CHCl_3_); ^1^H NMR (MeOD, 600 MHz) and ^13^C NMR (MeOD, 150 MHz), Table S10, (+)-HR-ESIMS found *m*/*z* 220.0983 [M + H]^+^ C_12_H_14_NO_3_, calculated for 220.0974; deposited in the GNPS spectral library, https://gnps.ucsd.edu/ProteoSAFe/gnpslibraryspectrum.jsp?SpectrumID=CCMSLIB00005725526#%7B%7D

### 4.8. GNPS Molecular Networking Based Metabolomics and Dereplication

For chemical profiling, the subextracts and fractions were prepared at 0.1 mg/mL and analyzed with an Acquity UPLC I-Class System coupled to an Acquity UPLC-PDA detector and the Xevo G2-XS QTof Mass Spectrometer (Waters^®^, Milford, MA, USA). 1 µL of sample was injected onto an Acquity UPLC HSS T3 column (High Strength Silica C_18_, 1.8 µm, 2.1 × 100 mm, Waters, Milford, MA, USA) operating at 40 °C. The mobile phases were H_2_O (A) and acetonitrile (B), each containing 0.1% of formic acid, with the following gradient: initial 99% A; 0–11.50 min (99% to 0% A); followed by washing and reconditioning of the column over 3.5 min. The total run time was 15 min. The MS1 and MS2 spectra were recorded in positive mode under the following conditions: capillary voltage: 3.0 kV, cone voltage: 40 V, source temperature: 150 °C, cone gas flow: 50 L/h, desolvation gas flow: 1200 L/h and a collision energy ramp with low collision energy: 6–60 eV and a high collision energy: 9-80 eV. Scan times were 0.1s and the acquisition range was *m*/*z* 50–1200. MassLynx^®^ Software (version 4.1) was used for data acquisition and analysis. Each sample was analyzed in triplicate to identify injection errors and one consensus chromatogram was selected for bioinformatic analysis.

Molecular networks were created with the Feature-Based Molecular Networking (FBMN) workflow [68] on GNPS [13] (https://gnps.ucsd.edu/ProteoSAFe/status.jsp?task=cd9f41245fc447fdbbf095d99ba80eab and https://gnps.ucsd.edu/ProteoSAFe/status.jsp?task=e75503bc8c094ce89d6d78752efef767). Therefore, the raw data files were converted to mzXML format using ProteoWizard and then imported to the MZmine 2 [68] software v2.39 for the preprocessing of the UPLC-QToF-MS/MS data. Mass detection was set to 1E^4^ for the MS1 level and 50 for MS2 levels. The chromatogram was built with ions showing a minimum time span of 0.01, minimum height of 2.5E^4^ and *m*/*z* tolerance 0.01 (or 5 ppm). The chromatogram was deconvoluted with the baseline algorithm (minimum peak height 2.5E^4^, peak duration 0.01–1.0 min, and baseline level 1E^4^). The isotope peak grouper algorithm with *m*/*z* tolerance of 0.01 (or 5 ppm) and RT tolerance 0.1 min was used for deisotoping. All samples were combined in a peak list using the join aligner algorithm; ions detected in the solvent and media blanks were removed from the mass list. The data was exported as .csv and .mgf files and uploaded to the GNPS platform for FBMN analysis. The data was filtered by removing all MS/MS fragment ions within +/− 17 Da of the precursor *m*/*z*. MS/MS spectra were window filtered by choosing only the top six fragment ions in the +/− 50 Da window throughout the spectrum. The precursor ion mass tolerance was set to 0.02 Da and the MS/MS fragment ion tolerance to 0.02 Da. A molecular network was then created where edges were filtered to have a cosine score above 0.7 and more than six matched peaks. Furthermore, edges between two nodes were kept in the network if and only if each of the nodes appeared in each other’s respective top 10 most similar nodes. Finally, the maximum size of a molecular family was set to 100, and the lowest scoring edges were removed from molecular families until the molecular family size was below this threshold. The spectra in the network were then searched against GNPS spectral libraries [13,69]. The library spectra were filtered in the same manner as the input data. All matches kept between network spectra and library spectra were required to have a score above 0.7 and at least six matched peaks. The molecular networks were visualized using Cytoscape [70] software v3.8.0.

For bioactive molecular networking, a publicly accessible R script available at (https://github.com/DorresteinLaboratory/Bioactive_Molecular_Networks/blob/master/Bioactive_Molecular_Networks_v1.1_MZmine2.r) was used to determine a bioactivity score for each ion in the samples. Samples were scaled by normalizing the intensity of the TIC followed by calculation of the Pearson correlation score (*r*) between the peak area of the ion and the bioactivity level. The outputted node attribute table was incorporated into Cytoscape to visualize the molecular network and to map out the bioactivity scores. 

The dereplication and molecular networking workflows available at the online GNPS platform (https://gnps.ucsd.edu/) combined with ISDB-UNPD dereplication [71] as well as manual dereplication (against the Dictionary of Natural Products (http://dnp.chemnetbase.com) and DEREP_NP [14]) databases, and reported literature data) were used for identification of putative compounds hits. Thereby, putative molecular formulae were generated and searched against the databases. Potential hits were then confirmed using CFM-ID (https://cfmid.wishartlab.com/), where *in silico* predicted MS/MS fragmentation patterns of the hit compound were compared against the experimental data.

### 4.9. Antimicrobial Activity Testing

Crude extracts were tested a final test concentration of 100 μg/mL for their antimicrobial activity. This included the ESKAPE panel of multidrug-resistant bacterial human pathogens, consisting of the Gram-positive bacteria *Enterococcus faecium* (Efm, DSM 20477) and methillicin-resistant *Staphylococcus aureus* (MRSA, DSM 18827), and the Gram-negative bacteria *Acinetobacter baumannii* (DSM 30007), *Klebsiella pneumoniae* (DSM 30104), *Escherichia coli* (DSM 1576), and *Pseudomonas aeruginosa* (DSM 1128). Furthermore, assays against six phytopathogens, three bacteria (*Pseudomonas syringae* DSM 50252, *Erwinia amylovora* DSM 50901 and *Ralstonia solanacearum* DSM 9544) and three fungi (*Phytophthora infestans* CBS 120920, *Pyricularia oryzae* DSM 62938, *Botrytis cinerea* DSM 5145) and two human pathogen yeasts, *Cryptococcus neoformans* (DSM 6973) and *Candida albicans* (DSM 1386), as well as two dermatophytic fungi, *Trichophyton rubrum* and *T. mentagrophytes*, were performed. All test strains were bought from the Leibniz Institute DSMZ-German Collection of Microorganisms and Cell Cultures, Germany and the CBS-KNAW culture collection, Utecht, Netherlands. Both dermatophytes were provided by Prof. Brasch (Universitäts-Hautklinik Kiel, Germany).

The cultivation of bacteria took place in a TSB medium (0.5% NaCl, 1.2% tryptic soy broth), but *E. faecium* was grown in a M92 medium (3% trypticase soy broth, pH 7.0–7.2, 0.3% yeast extract) and *R. solanacearum* in M186 medium (1% glucose, 0.5% peptone from soybeans, 0.3% yeast extract, 0.3% malt extract). The yeast *C. albicans* was cultivated in M186/3 (0.1% malt extract, 0.17% peptone from soymeal, 0.3% glucose, 0.1% yeast extract) and *C. neoformans* was grown in M186 medium. Overnight cultures of the test organisms were adjusted and diluted to an optical density (600 nm) of 0.01–0.03. To prepare the assay, stock solutions of the crude and Kupchan subextracts were prepared at 20 mg/mL in DMSO and transferred into a 96-well microliter plate. A total of 200 µL of cell suspension cultures were added to each well. The incubation of inoculated microplates was performed for 5 h at 37 °C and 200 rpm (*E. faecium* without shaking); 28 °C and 200 rpm for 7 h for the *C. neoformans* and the phytopathogenic bacteria. The detection of inhibitory effects was performed by adding 10 µL of a resazurin solution (0.3 mg/mL phosphate-buffered saline) to each well and incubating again for 5–60 min before the fluorescence signal (560 nm/590 nm) was read by the microplate reader (Tecan Infinite M200) as per Schneemann et al. [72]. For *E. faecium* the pH indicator bromocresol purple was used to determine the acidification caused by growing. For *R. solanacearum* and *C. neoformans*, the optical density at 600 nm after the incubation time was recorded using the microplate reader. Chloramphenicol was the positive control used for the bacteria except for *P. aeruginosa* (reference compound tolymyxin B), tetracycline for *R. solanacearum*, nystatin for *C. albicans* and amphotericin B for *C. neoformans*. A DMSO control was tested on the same plate. A threshold of 20% was used to identify extracts causing inhibition.

The cultivation of the filamentous fungi (phytopathogen and dermatophytes) took place in M186 medium except for *P. infestans* for which a pea medium (150 g peas, 5 g glucose, 1000 mL water, pH 6.5) was used. Each fungus was cultivated for 2 weeks on agar plates. A suspension of 5 × 10^4^ spores/mL medium (for *P. infestans* 1 × 10^4^ spores/mL medium) was prepared and added to each well of a microplate containing the test samples, which were transferred as described before (bacterial assay). After incubation for 72 h at 28 °C and 120 rpm, respectively 22 °C for phytopathogen fungi, the optical density at 600 nm was measured using the microplate reader Tecan Infinite M200. Clotrimazol was used as a positive control for the dermatophytes, cycloheximide for *P. infestans* and nystatin for *B. cinerea* and *P. oryzae*.

### 4.10. Anticancer Activity Testing

The crude extracts and fractions were tested in vitro against 2 human cancer cell lines; lung carcinoma cell line A549 (CLS, Eppelheim, Germany) and human breast cancer line MDA-MB-231 (CLS, Eppelheim, Germany), and the non-cancerous human keratinocyte line HaCaT (CLS, Eppelheim, Germany) at a concentration of 100 µg/mL. The antitumoral activity was evaluated by monitoring the metabolic activity using the CellTiterBlue Cell Viability Assay (Promega, Mannheim, Germany). HaCaT cells were cultivated in RPMI medium and A549 and MDA-MB-231 cells in DMEM:Ham’s F12 medium (1:1) supplemented with 15 mM HEPES. All media were supplemented with l-Glutamine, 10% fetal bovine serum, 100 U/mL penicillin, and 100 mg/mL streptomycin. The cultures were maintained at 37 °C under a humidified atmosphere and 5% CO_2_. The cell lines were transferred every 3 or 4 days. For the experimental procedure, the cells were seeded in 96-well plates at a concentration of 10,000 cells per well. A stock solution of 20 mg/mL in DMSO was prepared for each sample (crudes, subextracts, and pure compounds). After 24 h incubation, the medium was removed from the cells and 100 µL fresh medium containing the test samples was added. Each sample was prepared in duplicate once. Doxorubicin was used as positive control, 0.5% DMSO and growth media served as negative controls. Following compound addition, plates were cultured at 37 °C for 24 h. Afterwards, the assay was performed according to the manufacturer’s instructions and measured using the microplate reader Tecan Infinite M200 at excitation 560 nm and emission of 590 nm.

## Figures and Tables

**Table 1:** Anticancer and antimicrobial activities (% inhibition at 100 µg/mL) of the crude and Kupchan (K) subextracts of *R. mucilaginosa* 50-3-19/20B. Positive controls: anticancer activity: doxorubicin (10 µM); antimicrobial activity: chloramphenicol (10 µM).

Extract/Subextract		Cell Line			Pathogen	
		HaCaT	A549	MDA-MB-231	MRSA	*E. faecium*
PDA	Crude	-	81	35	-	30
	K-MeOH	-	-	-	-	-
	K-DCM	-	99	73	-	25
	K-Hexanes	-	-	-	74	51
WSP-30	Crude	-	-	-	82	81
	K-MeOH	-	-	-	-	-
	K-DCM	-	-	-	-	-
	K-Hexanes	-	-	-	90	98
Positive control		72	74	83	95	95

**Table 2:** Overview of different BGCs in marine *R. mucilaginosa* 50-3-19/20B and 10 other genomes of *Rhodotorula* deduced by AntiSMASH.

*Rhodotorula* Genomes	Genome Assembly Version	Genome Size (Mb)	Total BGCs	NRPS	Terpene	Others	Cf Putative	Cf Fatty Acid
Marine *R. mucilaginosa* 50-3-19/20B	marRhodv1 (denovo)	20.02	19	1	2	1	14	1
*R. mucilaginosa* IIPL32	ASM280678v1	20.15	21	1	2	1	16	1
*R. mucilaginosa* RIT389	RIT389_v1	19.7	16	1	2	0	12	1
*R. mucilaginosa* C2.5t1	ASM93196v1	19.98	20	1	2	1	15	1
*R. mucilaginosa* JGTA-S1	ASM305520v1	20.2	20	1	2	1	15	1
*R. graminis* WP1	Rhoba1_1	20.01	19	1	1	1	15	1
*R. toruloides* NP11	RHOziaDV1.0	20.2	19	1	1	1	15	1
*R. taiwanensis* MD1149	ASM292249.1	19.6	25	1	3	1	19	1
*R. kratochvilovae* LS11	ASM291796v1	22.1	21	1	2	1	16	1
*Rhodotorula* sp. FNED7-22	FNED7-bin-22	16.3	5	1	1	0	3	0
*Rhodotorula* sp. JG-1b	Rhosp1	19.4	28	1	4	1	21	1

Note 1: BGCs are predicted by AntiSMASH4, NRPS—Nonribosomal peptide synthetase cluster; Terpene—Terpene cluster; Others—Cluster containing a secondary metabolite-related protein that does not fit into any other category; Cf Fatty Acid—Putative fatty acid cluster identified with the ClusterFinder algorithm and Cf Putative—Putative cluster of unknown type identified with the ClusterFinder algorithm.

**Table 3:** NMR data for compounds **1** and **2** (MeOD, 600 MHz/150 MHz).

Position		1		2	
		*δ*_H_, Multiplicity (*J* in Hz)	*δ*_C_	*δ*_H_, Multiplicity (*J* in Hz)	*δ*_C_
1	a	3.63, m	64.8 (CH_2_)	3.63, m	64.7 (CH_2_)
	b	3.80, m		3.80, m	
2		3.79, m	70.3 (CH)	3.79, m	70.3 (CH)
3		3.69 m	72.7 (CH)	3.69, m	72.5 (CH)
4		3.48 m	70.4 (CH)	3.47, m	70.7 (CH)
5		3.87 m	70.0 (CH)	3.87, m	70.0 (CH)
6	a	4.16, m	67.7 (CH_2_)	4.18, m	67.8 (CH_2_)
	b	4.37, m		4.39, m	
7		-	172.1 (C)	-	172.9 (C)
8		2.05, s	20.3–20.8 (CH_3_)	2.08, s	20.4 (CH_3_)
9		-	173.1 (C)	-	-
10		2.03, s	20.3–20.8 (CH_3_)	-	-
11		-	172.9 (C)	-	-
12		2.08, s	20.6 (CH_3_)	-	-
1′		-	172.3 (C)	-	172.3 (C)
2′	a	2.61, m	39.8 (CH_2_)	2.65, m	39.8 (CH_2_)
	b	2.65, m		2.65, m	
3′		5.22, m	71.8 (CH)	5.23, m	71.7 (CH)
4′		1.61, m	34.7 (CH_2_)	1.62, m	34.8 (CH_2_)
5′		1.33, m	25.9 (CH_2_)	1.32, m	25.9 (CH_2_)
6′–13′		1.29–1.33, m	30.2–31.0 (CH_2_)	1.29–1.33, m	30.2–31.0 (CH_2_)
14′		1.29, m	32.9 (CH_2_)	1.29, m	32.8 (CH_2_)
15′		1.31, m	23.5 (CH_2_)	1.31, m	23.5 (CH_2_)
16′		0.90, t (6.9)	14.1 (CH_3_)	0.90, t (6.9)	14.2 (CH_3_)
17′		-	172.3 (C)		172.3 (C)
18′		2.02, s	20.8 (CH_3_)	2.02, s	20.8 (CH_3_)

**Table 4:** Sampling information of the six deep-sea *Rhodotorula* strains.

Isolate ID	Collection Date	Latitude	Longitude	Depth (m)	Sampling Gear
50-3-19/20B	6 October 2016	31° 39.62 N	38° 34.75 W	3602.7	Large Box Corer
52-1-0/1B	2 October 2016	32° 38.45 N	36° 15.36 W	3976.2	Large Box Corer
54-4-0-/1B	4 October 2016	34° 8.04 N	31° 8.92 W	3201.9	Large Box Corer
LR 28-14-1-1-1-1	26 September 2016	35° 39.02 N	35° 30.15 W	2877.7	Multi corer
LR 28-17-4-1	29 September 2017	33° 44.73 N	38° 52.41 W	3462.1	Large Box Corer
LR 5-2-4/4-1	29 September 2016	33° 54.89 N	38° 39.36 W	3138.6	Large Box Corer

## Data Availability

The genomic resource of this study is located at the github—https://github.com/drabhishekkumar/Genomics_Deep_Sea_Rhodotorula_mucilaginosa. The data used for the molecular networking analysis were deposited in the MassIVE Public GNPS database (http://massive.ucsd.edu) under access number MSV000085894.

## Supplementary Materials

The following are available online at https://www.mdpi.com/1660-3397/19/1/14/s1, bioactivity data; genomics tables; dereplication table; theoretical PEFA identification tables; GNPS MS/MS mirror plots of annotated compounds; spectral data of compounds **1**–**5**.
