# Supplementary material for: Genomics- and Metabolomics-Based Investigation of the Deep-Sea Sediment-Derived Yeast, Rhodotorula mucilaginosa 50-3-19/20B
Source: Mar Drugs. 2020 Dec 30;19(1):14. doi: 10.3390/md19010014 (PMC7823890; doi:10.3390/md19010014)
Supplement: Supplementary file 1 [file marinedrugs-19-00014-s001.pdf]

Supplementary Information

Genomics- and Metabolomics-Based Investigation of the Deep-Sea Sediment-Derived Yeast, *Rhodotorula mucilaginosa* 50-3-19/20B

Larissa Buedenbender [1,†], Abhishek Kumar [2,3,†], Martina Blümel [1], Frank Kempken [3,*] and Deniz Tasdemir [1,4,*]

[1] GEOMAR Centre for Marine Biotechnology (GEOMAR-Biotech), Research Unit Marine Natural Products Chemistry, GEOMAR Helmholtz Centre for Ocean Research Kiel, Am Kiel-Kanal 44, 24106 Kiel, Germany;
[2] Present Address: Institute of Bioinformatics, International Technology Park, Bangalore, 560066 & Manipal Academy of Higher Education (MAHE), Manipal 576104, Karnataka, India
[3] Department of Botany, Kiel University, Olshausenstr. 40, Kiel 24098, Germany
[4] Faculty of Mathematics and Natural Sciences, Kiel University, Christian-Albrechts-Platz 4, 24118 Kiel, Germany
[†] Equal contribution
[*] Correspondence: dtasdemir@geomar.de; Tel.: +49-431-600-4430 and fkempken@bot.uni-kiel.de, Tel.: +49 431 880-4274

<u>List of Tables</u>

	Page
**Table S1.** Bioactivity screening of extracts derived from six deep-sea *Rhodotorula* spp.	3
**Table S2.** Overview of the genomic locus for exo-inulinase enzyme in marine *R. mucilaginosa* 50-3-19/20B, as deduced via Omicsbox and Pfam annotations	4
**Table S3.** Annotation summary of putative exo-inulinase enzyme from *R. mucilaginosa* 50-3-19/20B and its homologs in different fungi	6
**Table S4.** Cytotoxic activity (%-inhibition at 100µg/mL) of the fractionated PDA-K-DCM subextract of *R. mucilaginosa* 50-3-19/20B against the breast cancer cell line MDA-MB-231	17
**Table S5.** Putative annotations of compounds detected in deep-sea *R. mucilaginosa* 50-3-19/20B extracts	18
**Table S6.** Theoretical masses of acetylated PEFAs	24
**Table S7.** MS/MS fragment ions indicative of the type of polyol groupp in PEFAs	25
**Table S8.** NMR data of compound **1** (MeOD, 600/150 MHz)	26
**Table S9.** NMR data for compound **2** (MeOD, 600/150 MHz)	27
**Table S10.** NMR data of compound **5** (MeOD, 600/150 MHz)	28

List of Figures

	Page
**Figure S1.** Differential metabolomes of *R. mucilaginosa* 50-3-19/20B	29
**Figure S2.** Overview of genomic statistics based on *de novo* genome assembly	29
**Figure S3.** GNPS MS/MS mirror plot of experimental and library data of 9-oxo-10*E*,12*Z*-octadecadienoic acid	30
**Figure S4.** GNPS MS/MS mirror plot of experimental and library data of cyclo-(Leu-Phe)	30
**Figure S5.** GNPS MS/MS mirror plot of experimental and library data of DL-Indole-3-lactic acid	31
**Figure S6.** GNPS MS/MS mirror plot of experimental and library data of 5α,8α-epidioxyergosta-6,22-dien-3β-ol	31
**Figure S7.** GNPS MS/MS mirror plot of experimental and library data of C17-sphinganine	32
**Figure S8.** GNPS MS/MS mirror plot of experimental and library data of 9,10-Epoxy-12-octadecenoic acid	32
**Figure S9.** (a) HR-ESIMS and (b) MS/MS spectra of **1**	33
**Figure S10.** ^{1}H NMR spectrum of compound **1** (MeOD, 600 MHz)	34
**Figure S11.** HSQC spectrum of compound **1** (MeOD, 600/150 MHz)	34
**Figure S12.** COSY spectrum of compound **1** (MeOD, 600 MHz)	35
**Figure S13.** HMBC spectrum of compound **1** (MeOD, 600/150 MHz)	35
**Figure S14.** NOESY spectrum of compound **1** (MeOD, 600 MHz)	36
**Figure S15.** TOCSY spectrum of compound **1** (MeOD, 600 MHz)	36
**Figure S16.** (a) HR-ESIMS and (b) MS/MS spectra of **2**	37
**Figure S17.** ^{1}H NMR spectrum of compound **2** (MeOD, 600 MHz)	38
**Figure S18.** HSQC spectrum of compound **2** (MeOD, 600/150 MHz)	38
**Figure S19.** COSY spectrum of compound **2** (MeOD, 600 MHz)	39
**Figure S20.** NOESY spectrum of compound **2** (MeOD, 600 MHz)	40
**Figure S21.** HMBC spectrum of compound **2** (MeOD, 600/150 MHz)	41
**Figure S22.** (a) HR-ESIMS and (b) MS/MS spectra of **3**	42
**Figure S23.** (a) HR-ESIMS and (b) MS/MS spectra of **4**	43
**Figure S24.** (a) HR-ESIMS and (b) MS/MS spectra of **5**	43
**Figure S25.** ^{1}H NMR spectrum of compound **5** (MeOD, 600 MHz)	44
**Figure S26.** ^{13}C NMR spectrum of compound **5** (MeOD, 150 MHz)	44
**Figure S27.** HSQC spectrum of compound **5** (MeOD, 600/150 MHz)	45
**Figure S28.** COSY spectrum of compound **5** (MeOD, 600 MHz)	45
**Figure S29.** HMBC spectrum of compound **5** (MeOD, 600/150 MHz)	46
**Figure S30.** NOESY spectrum of compound **5** (MeOD, 600 MHz)	46
**Figure S31.** Metabolites reported in the Dictionary of Natural Products for the genus *Rhodotorula*	47
**References**	48

Table S1. Bioactivity screening of extracts derived from six deep-sea *Rhodotorula* spp.

	Cell culture			ESKAPE panel								Phytopatogenic bacteria			Yeasts		Phytopathogenic fungi			Dermatophytes	
Sample	MB231	A375	HaCaT	Efm		MRSA		Kp	Ab	Psa	Ec	Pss	Ea	Rs	Ca	Cn	Pi	Po	Bc	Tr	Tm
				exp. 1	exp. 2	exp. 1	exp. 2														
50-3-19/20B_W	-	-	-	32	59	**99**	**96**	-	-	-	-	-	-	-	-	-	-	-	-	-	-
50-3-19/20B_P	74	73	20	-	37	-	-	-	-	-	-	-	-	-	-	-	-	-	-	-	-
52-1-0/1B_W	-	-	-	24	**100**	**99**	**99**	-	-	-	-	-	-	-	-	-	-	-	-	-	-
52-1-0/1B_P	-	-	-	78	**100**	**99**	**100**	-	-	-	-	-	-	-	-	-	-	-	-	-	-
54-4-0-/1B_W	-	-	-	**91**	**100**	**99**	**99**	-	-	-	-	-	-	-	-	-	-	-	-	-	-
54-4-0-/1B_P	-	-	-	-	**100**	-	**100**	-	22	-	-	-	-	-	-	-	-	-	-	-	-
LR 28-14-1-1-1-1_W	-	-	-	62	**86**	**99**	**96**	-	-	-	-	-	-	-	-	-	-	-	-	-	-
LR 28-14-1-1-1-1_P	-	-	-	**100**	71	**94**	**93**	-	-	-	-	-	-	-	-	-	-	-	-	-	-
LR 28-17-4-1_W	-	-	-	61	**90**	**100**	**99**	-	-	-	-	-	-	-	-	-	-	-	-	-	-
LR 28-17-4-1_P	-	-	-	**85**	**100**	**94**	**97**	-	-	-	-	-	-	29	-	-	-	-	-	-	-
LR 5-2-4/4-1_W	-	-	-	79	79	**98**	**96**	-	-	-	-	-	-	23	-	-	-	-	-	-	-
LR 5-2-4/4-1_P	-	-	-	**83**	53	**88**	**92**	-	-	-	-	-	-	-	-	-	-	-	-	-	-
WSP-30 blank	-	-	-	-	-	-	-	-	-	-	-	-	-	-	-	-	-	-	-	-	-
PDA blank	40	28	44	24	47	40	-	-	-	-	-	-	-	24	-	-	-	-	-	-	28
positive control	83	97	66	97	91	97	96	99	42	99	96	64	92	94	98	99	98	98	99	96	100
solvent control	-	-	-	-	-	-	-	-	-	-	-	-	-	-	-	-	-	-	-	-	-

MB231: breast cancer; A375: lung carcinoma; HaCaT: non-cancerous keratinocyte; Efm: *Enterococcus faecium*; MRSA: methillicin-resistant *Staphylococcus aureus*; Kp: *Klebsiella pneumoniae*; Ab: *Acinetobacter baumannii*; Psa: *Pseudomonas aeruginosa*; Ec: *Escherichia coli*; Pss: *Pseudomonas syringae*; Ea: *Erwinia amylovora*; Rs: *Ralstonia solanacearum*; Ca: *Candida albicans*; Cn: *Cryptococcus neoformans*; Pi: *Phytophthora infestans*; Po: *Pyricularia oryzae*; Bc: *Botrytis cinerea*; Tr: *Trichophyton rubrum*; Tm: *T. mentagrophytes*

Table S2. Overview of the genomic locus for exo-inulinase enzyme in marine *R. mucilaginosa* 50-3-19/20B, as deduced via Omicsbox and Pfam annotations.

Name	Description	Protein Length	Pfam annotation	Pfam Domain	Pfam ID	E-value	Start	End
g1614.t1	Endonuclease/exonuclease/phosphatase domain-containing protein	1604	Endonuclease/Exonuclease/phosphatase family	Exo_endo_phos	PF03372.22	9.0E-6	99	472
g1615.t1	hypothetical protein RHOSPDRAFT_19222	463	Translation initiation factor SUI1	SUI1	PF01253.21	3.6E-27	353	429
g1616.t1	---NA---	663	Permease for cytosine/purines, uracil, thiamine, allantoin	Transp_cyt_pur	PF02133.14	3.7E-42	326	579
			Permease for cytosine/purines, uracil, thiamine, allantoin	Transp_cyt_pur	PF02133.14	2.3E-25	100	275
g1617.t1	hypothetical protein BMF94_3082	66						
g1618.t1	FAD/NAD(P)-binding domain-containing protein	592	GDP dissociation inhibitor	GDI	PF00996.17	2.2E-32	5	284
g1619.t1	---NA---	101	Vacuolar protein sorting 55	Vps55	PF04133.13	1.2E-24	6	97
g1620.t1	hypothetical protein RHOSPDRAFT_34835	812						
g1621.t1	actin-like ATPase domain-containing protein	1956						
g1622.t1	NAD-P-binding protein	1910	short chain dehydrogenase	adh_short	PF00106.24	5.1E-24	1641	1861
g1623.t1	---NA---	1041						
g1624.t1	ARM repeat-containing protein	1943						
g1625.t1	hypothetical protein RHOSPDRAFT_35524	1198						
g1626.t1	L-iditol 2-dehydrogenase	617	Alcohol dehydrogenase GroES-like domain	ADH_N	PF08240.11	3.5E-24	59	171
			Zinc-binding dehydrogenase	ADH_zinc_N	PF00107.25	1.5E-13	211	343
g1627.t1	ferric reductase transmembrane component	778	Ferric reductase like transmembrane component	Ferric_reduct	PF01794.18	6.5E-18	186	302
			Ferric reductase NAD binding domain	NAD_binding_6	PF08030.11	1.5E-09	504	659
g1628.t1	protein of ctr copper transporter family	216	Ctr copper transporter family	Ctr	PF04145.14	6.2E-33	22	193
g1629.t1	beta-fructofuranosidase	679	Glycosyl hydrolases family 32 N-terminal domain	Glyco_hydro_32N	PF00251.19	6.5E-76	178	485
			Glycosyl hydrolases family 32 C terminal	Glyco_hydro_32C	PF08244.11	1.3E-5	506	673
g1630.t1	Dynactin, subunit p25	696	RING-variant domain	RINGv	PF12906.6	9.5E-13	341	391

g1631.t1	gpi anchored protein	275						
g1632.t1	lipoyl(octanoyl) transferase	356						
g1633.t1	hypothetical protein RHOSPDRAFT_32393	744	Glycine-rich domain-containing protein-like	GRDP-like	PF07173.11	6.8E-09	403	516
g1634.t1	voltage-gated chloride channel	1809	Voltage gated chloride channel	Voltage_CLC	PF00654.19	1.2E-85	1176	1538
			Rab-GTPase-TBC domain	RabGAP-TBC	PF00566.17	4.9E-52	481	683
			CBS domain	CBS	PF00571.27	8.1E-4	1680	1735
g1635.t1	hypothetical protein RHOSPDRAFT_24861	600						
g1636.t1	E3 ubiquitin-protein ligase UBR1	2049	Putative zinc finger in N-recognin (UBR box)	zf-UBR	PF02207.19	1.2E-15	115	189
			ATP-dependent Clp protease adaptor protein ClpS	ClpS	PF02617.16	1.5E-13	429	500
g1637.t1	carbon-nitrogen hydrolase	332	Carbon-nitrogen hydrolase	CN_hydrolase	PF00795.21	1.4E-26	34	319
g1638.t1	hypothetical protein RHOSPDRAFT_24864	805						
g1639.t1	hypothetical protein RHOSPDRAFT_24865	920						
g1640.t1	hypothetical protein RHOSPDRAFT_24865	564						
g1641.t1	NAD(P)-binding protein	303	Enoyl-(Acyl carrier protein) reductase	adh_short_C2	PF13561.5	8.6E-47	65	300
g1642.t1	Other/IRE protein kinase	1382	Ribonuclease 2-5A	Ribonuc_2-5A	PF06479.11	1.8E-45	1249	1375
			Protein kinase domain	Pkinase	PF00069.24	1.6E-31	941	1130
			Protein kinase domain	Pkinase	PF00069.24	1.3E-7	1156	1243
g1643.t1	C6 transcription factor	1042						
g1644.t1	taurine catabolism dioxygenase	377	Taurine catabolism dioxygenase TauD, TfdA family	TauD	PF02668.15	6E-40	54	352

Table S3. Annotation summary of putative exo-inulinase enzyme from *Rhodotorula mucilaginosa* 50-3-19/20B and its homologs in different fungi

Sequence ID	Species	Description	Protein Length	e-Value	sim mean	#GO	GO IDs	GO Names	Enzyme Codes	Enzyme Names	InterPro IDs	InterPro GO IDs	InterPro GO Names
g1629.t1	R. mucilaginosa 50-3¬-19/20B	glycoside hydrolase family 32 protein	679	0	74.14	3	P:GO:0005987; F:GO:0004575; C:GO:0000324	P:sucrose catabolic process; F:sucrose alpha-glucosidase activity; C:fungal-type vacuole	EC:3.2.1.20; EC:3.2.1.26; EC:3.2.1.48	Alpha-glucosidase; Beta-fructofuranosidase; Sucrose alpha-glucosidase	IPR013189 (PFAM); G3DSA:2.60.120.560 (GENE3D); IPR023296 (G3DSA:2.115.10.GENE3D); IPR013148 (PFAM); mobidb-lite (MOBIDB_LITE); PTHR42800:SF2 (PANTHER); PTHR42800 (PANTHER); cd18622 (CDD); IPR013320 (SUPERFAMILY); IPR023296 (SUPERFAMILY)	no GO terms	no GO terms
AZR37516.1	Rhodotorula paludigena	beta-fructofurano sidase	617	0	68.7	3	P:GO:0005987; F:GO:0004575; C:GO:0000324	P:sucrose catabolic process; F:sucrose alpha-glucosidase activity; C:fungal-type vacuole	EC:3.2.1.20; EC:3.2.1.26; EC:3.2.1.48	Alpha-glucosidase; Beta-fructofuranosidase; Sucrose alpha-glucosidase	IPR023296 (G3DSA:2.115.10.GENE3D); IPR013148 (PFAM); G3DSA:2.60.120.560 (GENE3D); mobidb-lite (MOBIDB_LITE); mobidb-lite (MOBIDB_LITE); PTHR42800:SF2 (PANTHER); PTHR42800 (PANTHER); IPR018053 (PROSITE_PATTERNS);	P:GO:0005975; F:GO:0004553	P:carbohydrate metabolic process; F:hydrolase activity, hydrolyzing O-glycosyl compounds

											cd18622 (CDD); IPR023296 (SUPERFAMILY); IPR013320 (SUPERFAMILY)		
TKA53735.1	Rhodotorula sp. CCFEE 5036	glycoside hydrolase family 32 protein	676	0	74.85	2	P:GO:0005975; F:GO:0004553	P:carbohydrate metabolic process; F:hydrolase activity, hydrolyzing O-glycosyl compounds			IPR023296 (G3DSA:2.115.10.GENE3D); IPR013189 (PFAM); G3DSA:2.60.120.560 (GENE3D); IPR013148 (PFAM); mobidb-lite (MOBIDB_LITE); mobidb-lite (MOBIDB_LITE); PTHR42800:SF2 (PANTHER); PTHR42800 (PANTHER); IPR018053 (PROSITE_PATTERNS); cd18622 (CDD); IPR013320 (SUPERFAMILY); IPR023296 (SUPERFAMILY)	P:GO:0005975; F:GO:0004553	P:carbohydrate metabolic process; F:hydrolase activity, hydrolyzing O-glycosyl compounds
KWU45911.1	Rhodotorula sp. JG-1b	glycoside hydrolase family 32 protein	559	0	74.92	2	P:GO:0005975; F:GO:0004553	P:carbohydrate metabolic process; F:hydrolase activity, hydrolyzing O-glycosyl compounds			IPR013189 (PFAM); IPR013148 (PFAM); IPR023296 (G3DSA:2.115.10.GENE3D); G3DSA:2.60.120.560 (GENE3D); mobidb-lite (MOBIDB_LITE); mobidb-lite (MOBIDB_LITE); PTHR42800:SF2 (PANTHER); PTHR42800 (PANTHER); IPR018053	P:GO:0005975; F:GO:0004553	P:carbohydrate metabolic process; F:hydrolase activity, hydrolyzing O-glycosyl compounds

											(PROSITE_PATTERNS); cd18622 (CDD); IPR013320 (SUPERFAMILY); IPR023296 (SUPERFAMILY)		
POY72393.1	Rhodotorula taiwanensis	beta-fructofuranosidase	614	0	73.96	2	P:GO:0005975; F:GO:0004553	P:carbohydrate metabolic process; F:hydrolase activity, hydrolyzing O-glycosyl compounds			G3DSA:2.60.120.560 (GENE3D); IPR023296 (G3DSA:2.115.10.GENE3D); IPR013189 (PFAM); IPR013148 (PFAM); mobidb-lite (MOBIDB_LITE); PTHR42800 (PANTHER); PTHR42800:SF2 (PANTHER); IPR018053 (PROSITE_PATTERNS); cd18622 (CDD); IPR013320 (SUPERFAMILY); IPR023296 (SUPERFAMILY)	P:GO:0005975; F:GO:0004553	P:carbohydrate metabolic process; F:hydrolase activity, hydrolyzing O-glycosyl compounds
GFZ51952.1	Saitozyma sp. JCM 24511	beta-fructofuranosidase	523	0	77.08	4	P:GO:0005987; F:GO:0004575; C:GO:0000324; C:GO:0016021	P:sucrose catabolic process; F:sucrose alpha-glucosidase activity; C:fungal-type vacuole; C:integral component of membrane	EC:3.2.1.20; EC:3.2.1.26; EC:3.2.1.48	Alpha-glucosidase; Beta-fructofuranosidase; Sucrose alpha-glucosidase	IPR013189 (PFAM); IPR023296 (G3DSA:2.115.10.GENE3D); IPR013148 (PFAM); G3DSA:2.60.120.560 (GENE3D); mobidb-lite (MOBIDB_LITE); PTHR42800:SF2 (PANTHER); PTHR42800 (PANTHER); IPR018053 (PROSITE_PATTERNS); cd18622 (CDD); IPR023296	P:GO:0005975; F:GO:0004553	P:carbohydrate metabolic process; F:hydrolase activity, hydrolyzing O-glycosyl compounds

											(SUPERFAMILY); IPR013320 (SUPERFAMILY)		
KKY24889.1	Diplodia seriata	glycoside hydrolase family 32 protein	563	0	85.94	2	P:GO:0005975; F:GO:0004553	P:carbohydrate metabolic process; F:hydrolase activity, hydrolyzing O-glycosyl compounds			IPR023296 (G3DSA:2.115.10.GENE3D); IPR013148 (PFAM); IPR013189 (PFAM); G3DSA:2.60.120.560 (GENE3D); PTHR42800:SF2 (PANTHER); PTHR42800 (PANTHER); IPR018053 (PROSITE_PATTERNS); cd18622 (CDD); IPR013320 (SUPERFAMILY); IPR023296 (SUPERFAMILY)	P:GO:0005975; F:GO:0004553	P:carbohydrate metabolic process; F:hydrolase activity, hydrolyzing O-glycosyl compounds
OMP85659.1	Diplodia seriata	glycoside hydrolase family 32 protein	563	0	86.07	2	P:GO:0005975; F:GO:0004553	P:carbohydrate metabolic process; F:hydrolase activity, hydrolyzing O-glycosyl compounds			IPR013148 (PFAM); IPR013189 (PFAM); IPR023296 (G3DSA:2.115.10.GENE3D); G3DSA:2.60.120.560 (GENE3D); PTHR42800:SF2 (PANTHER); PTHR42800 (PANTHER); IPR018053 (PROSITE_PATTERNS); cd18622 (CDD); IPR013320 (SUPERFAMILY); IPR023296 (SUPERFAMILY)	P:GO:0005975; F:GO:0004553	P:carbohydrate metabolic process; F:hydrolase activity, hydrolyzing O-glycosyl compounds

XP_035362863.1	Lasiodiplodia theobromae	glycoside hydrolase family 32 protein	562	0	86.11	2	P:GO:0005975; F:GO:0004553	P:carbohydrate metabolic process; F:hydrolase activity, hydrolyzing O-glycosyl compounds			IPR013148 (PFAM); IPR013189 (PFAM); IPR023296 (G3DSA:2.115.10.GENE3D); G3DSA:2.60.120.560 (GENE3D); PTHR42800:SF2 (PANTHER); PTHR42800 (PANTHER); IPR018053 (PROSITE_PATTERNS); cd18622 (CDD); IPR023296 (SUPERFAMILY); IPR013320 (SUPERFAMILY)	P:GO:0005975; F:GO:0004553	P:carbohydrate metabolic process; F:hydrolase activity, hydrolyzing O-glycosyl compounds
XP_020129257.1	Diplodia corticola	glycoside hydrolase family 32 protein	563	0	85.81	2	P:GO:0005975; F:GO:0004553	P:carbohydrate metabolic process; F:hydrolase activity, hydrolyzing O-glycosyl compounds			G3DSA:2.60.120.560 (GENE3D); IPR013189 (PFAM); IPR023296 (G3DSA:2.115.10.GENE3D); IPR013148 (PFAM); PTHR42800 (PANTHER); PTHR42800:SF2 (PANTHER); IPR018053 (PROSITE_PATTERNS); cd18622 (CDD); IPR023296 (SUPERFAMILY); IPR013320 (SUPERFAMILY)	P:GO:0005975; F:GO:0004553	P:carbohydrate metabolic process; F:hydrolase activity, hydrolyzing O-glycosyl compounds
KAF4312946.1	Botryosphaeria dothidea	glycoside hydrolase family 32 protein	562	0	85.72	3	P:GO:0005975; F:GO:0004553; C:GO:0016021	P:carbohydrate metabolic process; F:hydrolase activity,			IPR013148 (PFAM); IPR023296 (G3DSA:2.115.10.GENE3D); IPR013189 (PFAM); G3DSA:2.60.120.560 (GENE3D);	P:GO:0005975; F:GO:0004553	P:carbohydrate metabolic process; F:hydrolase

								hydrolyzing O-glycosyl compounds; C:integral component of membrane			PTHR42800 (PANTHER); PTHR42800:SF2 (PANTHER); IPR018053 (PROSITE_PATTERNS); cd18622 (CDD); IPR013320 (SUPERFAMILY); IPR023296 (SUPERFAMILY)		activity, hydrolyzing O-glycosyl compounds
KIJ52043.1	Sphaerobolus stellatus SS14	glycoside hydrolase family 32 protein	562	0	84.23	2	P:GO:0005975; F:GO:0004553	P:carbohydrate metabolic process; F:hydrolase activity, hydrolyzing O-glycosyl compounds			IPR013148 (PFAM); IPR013189 (PFAM); IPR023296 (G3DSA:2.115.10.GENE3D); G3DSA:2.60.120.560 (GENE3D); PTHR42800 (PANTHER); PTHR42800:SF2 (PANTHER); IPR018053 (PROSITE_PATTERNS); cd18622 (CDD); IPR013320 (SUPERFAMILY); IPR023296 (SUPERFAMILY)	P:GO:0005975; F:GO:0004553	P:carbohydrate metabolic process; F:hydrolase activity, hydrolyzing O-glycosyl compounds
THU96730.1	Dendrothele bispora CBS 962.96	glycoside hydrolase family 32 protein	523	0	84.95	3	P:GO:0005975; F:GO:0004553; C:GO:0016021	P:carbohydrate metabolic process; F:hydrolase activity, hydrolyzing O-glycosyl compounds; C:integral			G3DSA:2.60.120.560 (GENE3D); IPR023296 (G3DSA:2.115.10.GENE3D); IPR013148 (PFAM); IPR013189 (PFAM); PTHR42800 (PANTHER); PTHR42800:SF2 (PANTHER); IPR018053 (PROSITE_PATTERNS);	P:GO:0005975; F:GO:0004553	P:carbohydrate metabolic process; F:hydrolase activity, hydrolyzing O-glycosyl compounds

								component of membrane			cd18622 (CDD); IPR013320 (SUPERFAMILY); IPR023296 (SUPERFAMILY)		
XP_008034329.1	Trametes versicolor FP-101664 SS1	glycoside hydrolase family 32 protein	540	0	83.59	3	P:GO:0005975; F:GO:0004553; C:GO:0016021	P:carbohydrate metabolic process; F:hydrolase activity, hydrolyzing O-glycosyl compounds; C:integral component of membrane			IPR013189 (PFAM); G3DSA:2.60.120.560 (GENE3D); IPR023296 (G3DSA:2.115.10.GENE3D); IPR013148 (PFAM); PTHR42800 (PANTHER); PTHR42800:SF2 (PANTHER); IPR018053 (PROSITE_PATTERNS); cd18622 (CDD); IPR023296 (SUPERFAMILY); IPR013320 (SUPERFAMILY)	P:GO:0005975; F:GO:0004553	P:carbohydrate metabolic process; F:hydrolase activity, hydrolyzing O-glycosyl compounds
KAF5355425.1	Tetrapyrgos nigripes	glycoside hydrolase family 32 protein	521	0	83.08	3	P:GO:0005975; F:GO:0004553; C:GO:0016021	P:carbohydrate metabolic process; F:hydrolase activity, hydrolyzing O-glycosyl compounds; C:integral component of membrane			IPR013148 (PFAM); G3DSA:2.60.120.560 (GENE3D); IPR023296 (G3DSA:2.115.10.GENE3D); IPR013189 (PFAM); PTHR42800:SF2 (PANTHER); PTHR42800 (PANTHER); IPR018053 (PROSITE_PATTERNS); cd18622 (CDD); IPR013320	P:GO:0005975; F:GO:0004553	P:carbohydrate metabolic process; F:hydrolase activity, hydrolyzing O-glycosyl compounds

											(SUPERFAMILY); IPR023296 (SUPERFAMILY)		
KAE8543285.1	Cryptococcus cf. gattii	beta-fructofuranosidase	519	0	97.55	3	P:GO:0005987; F:GO:0004575; C:GO:0000324	P:sucrose catabolic process; F:sucrose alpha-glucosidase activity; C:fungal-type vacuole	EC:3.2.1.20; EC:3.2.1.26; EC:3.2.1.48	Alpha-glucosidase; Beta-fructofuranosidase; Sucrose alpha-glucosidase	G3DSA:2.60.120.560 (GENE3D); IPR013189 (PFAM); IPR023296 (G3DSA:2.115.10.GENE3D); IPR013148 (PFAM); mobidb-lite (MOBIDB_LITE); PTHR42800 (PANTHER); PTHR42800:SF2 (PANTHER); IPR018053 (PROSITE_PATTERNS); cd18622 (CDD); IPR023296 (SUPERFAMILY); IPR013320 (SUPERFAMILY)	P:GO:0005975; F:GO:0004553	P:carbohydrate metabolic process; F:hydrolase activity, hydrolyzing O-glycosyl compounds
OSD00128.1	Trametes coccinea BRFM310	glycoside hydrolase family 32 protein	524	0	83.56	3	P:GO:0005975; F:GO:0004553; C:GO:0016021	P:carbohydrate metabolic process; F:hydrolase activity, hydrolyzing O-glycosyl compounds; C:integral component of membrane			IPR013148 (PFAM); G3DSA:2.60.120.560 (GENE3D); IPR013189 (PFAM); IPR023296 (G3DSA:2.115.10.GENE3D); PTHR42800 (PANTHER); PTHR42800:SF2 (PANTHER); IPR018053 (PROSITE_PATTERNS); cd18622 (CDD); IPR023296 (SUPERFAMILY); IPR013320 (SUPERFAMILY)	P:GO:0005975; F:GO:0004553	P:carbohydrate metabolic process; F:hydrolase activity, hydrolyzing O-glycosyl compounds

EOD51241.1	Neofusicoccum parvum UCRNP2	glycoside hydrolase family 32 protein	563	0	84.03	2	P:GO:0005975; F:GO:0004553	P:carbohydrate metabolic process; F:hydrolase activity, hydrolyzing O-glycosyl compounds			IPR013148 (PFAM); IPR023296 (G3DSA:2.115.10.GENE3D); G3DSA:2.60.120.560 (GENE3D); mobidb-lite (MOBIDB_LITE); PTHR42800:SF2 (PANTHER); PTHR42800 (PANTHER); IPR018053 (PROSITE_PATTERNS); cd18622 (CDD); IPR023296 (SUPERFAMILY); IPR013320 (SUPERFAMILY)	P:GO:0005975; F:GO:0004553	P:carbohydrate metabolic process; F:hydrolase activity, hydrolyzing O-glycosyl compounds
KAF2091127.1	Saccharata proteae CBS 121410	SCF E3 ubiquitin ligase complex F-box protein	537	0	79.88	4	P:GO:0005975; P:GO:0006629; F:GO:0004553; F:GO:0008081	P:carbohydrate metabolic process; P:lipid metabolic process; F:hydrolase activity, hydrolyzing O-glycosyl compounds; F:phosphoric diester hydrolase activity			G3DSA:2.60.120.560 (GENE3D); IPR023296 (G3DSA:2.115.10.GENE3D); IPR013148 (PFAM); PTHR42800:SF2 (PANTHER); PTHR42800 (PANTHER); IPR018053 (PROSITE_PATTERNS); cd18622 (CDD); IPR023296 (SUPERFAMILY); IPR013320 (SUPERFAMILY)	P:GO:0005975; F:GO:0004553	P:carbohydrate metabolic process; F:hydrolase activity, hydrolyzing O-glycosyl compounds
KIR51126.1	Cryptococcus gattii Ru294	beta-fructofuranosidase	519	0	97.81	3	P:GO:0005987; F:GO:0004575; C:GO:0000324	P:sucrose catabolic process; F:sucrose alpha-glucosidase	EC:3.2.1.20; EC:3.2.1.26; EC:3.2.1.48	Alpha-glucosidase; Beta-fructofuranosidase;	IPR023296 (G3DSA:2.115.10.GENE3D); G3DSA:2.60.120.560 (GENE3D);	P:GO:0005975; F:GO:0004553	P:carbohydrate metabolic process;

								activity; C:fungal-type vacuole		Sucrose alpha-glucosidase	IPR013189 (PFAM); IPR013148 (PFAM); PTHR42800 (PANTHER); PTHR42800:SF2 (PANTHER); IPR018053 (PROSITE_PATTERNS); cd18622 (CDD); IPR023296 (SUPERFAMILY); IPR013320 (SUPERFAMILY)		F:hydrolase activity, hydrolyzing O-glycosyl compounds
XP_567775.1	Cryptococcus neoformans var. neoformans JEC21	beta-fructofuranosidase	519	0	96.37	3	P:GO:0005987; F:GO:0004575; C:GO:0000324	P:sucrose catabolic process; F:sucrose alpha-glucosidase activity; C:fungal-type vacuole	EC:3.2.1.20; EC:3.2.1.26; EC:3.2.1.48	Alpha-glucosidase; Beta-fructofuranosidase; Sucrose alpha-glucosidase	G3DSA:2.60.120.560 (GENE3D); IPR023296 (G3DSA:2.115.10.GENE3D); IPR013189 (PFAM); IPR013148 (PFAM); PTHR42800:SF2 (PANTHER); PTHR42800 (PANTHER); IPR018053 (PROSITE_PATTERNS); cd18622 (CDD); IPR023296 (SUPERFAMILY); IPR013320 (SUPERFAMILY)	P:GO:0005975; F:GO:0004553	P:carbohydrate metabolic process; F:hydrolase activity, hydrolyzing O-glycosyl compounds
KIR83202.1	Cryptococcus gattii VGIV IND107	beta-fructofuranosidase	519	0	97.96	3	P:GO:0005987; F:GO:0004575; C:GO:0000324	P:sucrose catabolic process; F:sucrose alpha-glucosidase activity; C:fungal-type vacuole	EC:3.2.1.20; EC:3.2.1.26; EC:3.2.1.48	Alpha-glucosidase; Beta-fructofuranosidase; Sucrose alpha-glucosidase	IPR013189 (PFAM); IPR023296 (G3DSA:2.115.10.GENE3D); IPR013148 (PFAM); G3DSA:2.60.120.560 (GENE3D); PTHR42800:SF2 (PANTHER); PTHR42800 (PANTHER); IPR018053	P:GO:0005975; F:GO:0004553	P:carbohydrate metabolic process; F:hydrolase activity, hydrolyzing O-

											(PROSITE_PATTERNS); cd18622 (CDD); IPR023296 (SUPERFAMILY); IPR013320 (SUPERFAMILY)		glycosyl compounds

Table S4. Cytotoxic activity (%-inhibition at 100 µg/mL) of the fractionated PDA-K-DCM subextract of *R. mucilaginosa* 50-3-19/20B against the breast cancer cell line MDA-MB-231

Fraction	MDA-MB-231
F1	-
F2	-
F3	-
F4	27
F5-8	22
F9-12	-
F13-16	-
F17	-
F18	-
F19	44
F20	51
**F21**	**86**
**F22**	**94**
**F23**	**88**
**F24**	**97**
F25	52
F26	-
F27	-
F28	-
F29	-
F30	-
F31	-

Table S5. Putative annotations of compounds detected in deep-sea *R. mucilaginosa* 50-3-19/20B extracts. Annotations were based on GNPS and manual dereplication of *m/z* ([M+H]+ or [M+Na]+), retention time (t_R), fragmentation pattern and predicted molecular formula against Dictionary of Natural Products, DEREP_NP and other literature data. Confidence levels of putative identification according to Sumner et al. [1] and Blaženovic et al. [2].

I.D	*m/z*	t_R (min)	MS/MS (*m/z*)*	Ion type	Molecular formula (M)	Identified compound	Compound class	Confidence	Reference
	261.1304	2.15	233.17; 216.14; 188.14; 120.08	$[M+H]^+$	$C_{15}H_{20}N_2O_2$	Cyclo-(Leu-Phe)	Diketopiperazine	2	[3]
	188.0713	3.83	170.06; 146.06; 118.07	$[M-H_2O+H]^+$	$C_{11}H_{11}NO_3$	DL-Indole-3-lactic acid	Indole alkaloid	2	[4]
	160.0764	3.84	132.08; 118.06	$[M+H]^+$	$C_{10}H_{11}NO_4S$	1-Hydroxy-2-(1H-indol-3-yl)ethanesulfonic acid	Indole alkaloid	3	
	206.08	3.94	188.07; 170.06; 160.08; 146.06; 130.07118.07	$[M+H]^+$	$C_{11}H_{11}NO_3$	DL-Indole-3-lactic acid	Indole alkaloid	2	[4]
	603.5312	9.54	273.10; 235.21; 231.09; 217.20; 189.08; 171.07; 161.13; 153.06; 135.12; 121.10; 111.04; 95.09; 81.07	$[M+H]^+$	$C_{40}H_{58}O_4$	Tetrahydroxydihydrolycopene	Carotenoid	3	[5]
	571.6362	10.37	263.24; 245.23; 231.09; 189.08; 175.15; 161.13; 153.06; 147.12; 133.10; 121.10; 111.05; 93.03; 81.07	$[M+H]^+$	$C_{40}H_{60}O_2$	Dihydroxylycopene	Carotenoid	3	[5]
	553.5593	15.17	451.34; 391.32; 293.28; 275.27; 233.23; 219.21; 201.08; 177.16; 163.15; 149.13; 141.06; 135.12; 121.10; 109.10; 97.10; 81.03	$[M+H]^+$	$C_{40}H_{56}O$	Cryptoxanthin	Carotenoid	3	[5]
**5**	220.0983	5.10	202.09; 170.06; 160.08; 132.08; 118.07	$[M+H]^+$	$C_{12}H_{14}NO_3$	Methyl 2-hydroxy-3-(1H-indol-2-yl)propanoate	Indole	1	[6]
	411.3263	14.01	216535.14; 191.11; 173.10; 145.10	$[M-H_2O+H]^+$	$C_{28}H_{44}O_3$	5α,8α-Epidioxyergosta-6,22-dien-3β-ol	Sterol	2	[7]
	288.2906	7.76	270.28; 106.09; 88.08	$[M+H]^+$	$C_{17}H_{37}NO_2$	C17-Sphinganine	Aminolipid	2	[8]
PEFA (Polyol ester of fatty acid) glycolipid annotations *(in molecular clusters as per Figure 4 in the main text)*									
	767.4197	15.59	**707.40**; **679. 37**; **647.38**; **619.34**; **587.36**; 517.31; 425.14; 365.12; 337.09; 265.25; 181.09; 153.05; 111.04	$[M+Na]^+$	$C_{38}H_{64}O_{14}$	Mannitol-pentaacetate-3-acetyloxy-C20	PEFA	2	

	739.4256	15.48	**679.44**; **619.42**; **559.37**; 535.33; **397.18**; **369.11**; 365.27; 337.16; 309.09; 265.25; 253.06; 237.15; 199.14; 111.04	$[M+Na]^+$	$C_{36}H_{60}O_{14}$	Mannitol-pentaacetate-3-acetyloxy-C18	PEFA	3	[9,10]
	695.3984	15.40	**635.38**; **575.36**; 553.37; 533.34; **515.34**; 493.35; 451.35; 393.30; **325.09**; 293.28; 275.27; 265.07; 201.08; 141.04	$[M+Na]^+$	$C_{35}H_{60}O_{12}$	Arabitol-tetraacetate-3-acetyloxy-C20	PEFA	3	
	613.2845	11.44	**553.26**; **493.24**; **433.22**; **411.24**; 373.20; **355.10**; **295.08**; 281.17; **235.06**. 181.16; 153.06; 111.05	$[M+Na]^+$	$C_{28}H_{46}O_{13}$	Mannitol-tetraacetate-3-acetyloxy-C12	PEFA	2	
	641.3152	12.62	**581.29**; **521.27**; **461.25**; 439.27; **401.23**; **355.19**; 309.20; **295.08**; 273.10; **235.06**; 209.19; 153.06; 111.04	$[M+Na]^+$	$C_{30}H_{50}O_{13}$	Mannitol-tetraacetate-3-acetyloxy-C14	PEFA	2	[10,11]
	669.4626	13.61	**609.33**; **549.30**; **489.28**; 467.30; **429.26**; 407.28; **355.10**; 337.24; **295.08**; 273.10; 237.22; **235.06**; 231.09; 219.21; 171.07; 153.06; 135.12; 121.10; 115.04; 111.05	$[M+Na]^+$	$C_{32}H_{54}O_{13}$	Mannitol-tetraacetate-3-acetyloxy-C16	PEFA	3	[9-11]
**3**	697.3560	14.51	**637.36**; **577.34**; 535.33; **517.32**; 495.33; **457.29**; 435.31; 365.27; **355.11**; 337.27; **295.08**; 273.10; 265.25; **235.06**; 231.09; 171.07; 153.06; 135.12; 121.10; 111.05	$[M+Na]^+$	$C_{34}H_{58}O_{13}$	Mannitol-tetraacetate-3-acetyloxy-C18	PEFA	3	[9-11]
	725.4203	15.22	**665.39**; **605.37**; **545.35**; 523.36; **485.32**; 393.30; **355.10**; **295.08**; 273.10; **235.06**; 231.09; 171.07; 153.06; 135.12; 111.05	$[M+Na]^+$	$C_{36}H_{62}O_{13}$	Mannitol-tetraacetate-3-acetyloxy-C20	PEFA	3	[10]
	555.3145	12.70	**495.29**; **435.27**; 379.25; **375.25**; 353.27; 337.24; 289.27; **241.07**; 237.22; 219.21; 213.09; 195.09; 181.05; 159.07; 135.12; 121.10; 99.04	$[M+Na]^+$	$C_{27}H_{48}O_{10}$	Arabitol-diacetate-3-acetyloxy-C16	PEFA	2	[10]

	583.5362	13.83	**523.33**; **463.31**; 407.28; **403.28**; 381.30; 365.27; 265.25; **241.07**; 181.05; 159.07; 135.12; 99.05	$[M+Na]^+$	$C_{29}H_{52}O_{10}$	Arabitol-diacetate-3-acetyloxy-C18	PEFA	3	
	529.3364	9.33	**469.24**; 451.23; **409.22**; 392.21; 383.16; **349.20**; 323.18; 281.17; **271.08**; 153.06; 111.05	$[M+Na]^+$	$C_{24}H_{42}O_{11}$	Mannitol-diacetate-3-acetyloxy-C12	PEFA	3	
	557.2955	10.97	**497.27**; 479.26; **437.25**; 419.24; 395.24; **377.23**; 351.21; 313.09; 309.20; **271.08**; 253.07; 211.06	$[M+Na]^+$	$C_{26}H_{46}O_{11}$	Mannitol-diacetate-3-acetyloxy-C14	PEFA	2	[11]
	585.4353	12.32	**525.30**; 508.30; **465.28**; 447.27; **405.26**; 381.25; 337.24; 313.09; **271.08**; 253.07; 211.06; 129.06; 111.08	$[M+Na]^+$	$C_{28}H_{50}O_{11}$	Mannitol-diacetate-3-acetyloxy-C16	PEFA	3	[10]
	613.3557	13.51	**553.34**; 535.33; **493.31**; 475.30; **433.29**; 407.27; 365.27; **271.08**, 211.06	$[M+Na]^+$	$C_{30}H_{54}O_{11}$	Mannitol-diacetate-3-acetyloxy-C18	PEFA	2	[10,11]
	417.2099	6.63	**357.19**; 339.18; 253.14; **187.06**	$[M+Na]^+$	$C_{18}H_{34}O_9$	Mannitol- 3-acetyloxy C10	PEFA	2	
	445.2415	8.02	**385.23**; 281.17; **187.06**	$[M+Na]^+$	$C_{20}H_{38}O_9$	Mannitol- 3-acetyloxy C12	PEFA	2	
	473.4443	9.44	**413.25**; 309.20; **187.06**	$[M+Na]^+$	$C_{22}H_{42}O_9$	Mannitol- 3-acetyloxy C14	PEFA	3	
	501.2922	10.73	**441.28**; 415.19; 397.17; 379.16; 337.23; **187.06**	$[M+Na]^+$	$C_{24}H_{46}O_9$	Mannitol- 3-acetyloxy C16	PEFA	2	[11]
	515.2836	10.11	**455.26**; 437.25; **495.24**; 309.20; **229.07**	$[M+Na]^+$	$C_{24}H_{44}O_{10}$	Mannitol-monocetate-3-acetyloxy-C18	PEFA	2	[11]
**2**	543.3145	11.54	**483.29**; **423.27**; 337.24; **229.07**	$[M+Na]^+$	$C_{26}H_{48}O_{10}$	Mannitol-monocetate-3-acetyloxy-C16	PEFA	2	
	571.3468	12.57	**511.32**; 493.31; **451.30**; 365.27; **229.07**	$[M+Na]^+$	$C_{28}H_{52}O_{10}$	Mannitol-monocetate-3-acetyloxy-C18	PEFA	2	
	655.2946	12.32	**595.27**; **535.25**; 513.27; 494.25; **475.23**; 453.25; 433.22; 411.24; **397.11**; **337.09**; 281.17; 273.10; 231.09; 213.08; 181.16; 153.06; 111.05	$[M+Na]^+$	$C_{30}H_{48}O_{14}$	Mannitol-pentacetate-3-acetyloxy-C12	PEFA	2	
	739.3934	14.86	**679.37**; **619.35**; **597.36**; 577.34; **559.33**; **537.34**; 517.31; **499.30**; 495.33; 457.29; 435.31; **397.11**; 365.27; **337.09**; 315.11;	$[M+Na]^+$	$C_{36}H_{60}O_{14}$	Mannitol-pentacetate-3-acetyloxy-C18	PEFA	2	[9,10]

			295.08; 273.10; 265.25; 247.24; 231.09; 213.08; 171.07; 153.06; 111.05						
	683.3955	14.87	**623.38**; **563.36**; **503.33**; **443.30**; 435.31 393.30; **313.09**; 293.28; 253.07; 231.09; 211.06; 193.05; 189.08; 171.07; 153.06; 111.05	[M+Na]+	$C_{34}H_{60}O_{12}$	Mannitol-triacetate-3-acetyloxy-C20	PEFA	2	[10]
	655.3449	13.92	**595.35**; **535.33; 475.30**; 453.32; **415.28**; 365.27; **313.09**; 265.25; 253.07; 231.09; 189.08; 153.06; 111.05	[M+Na]+	$C_{32}H_{56}O_{12}$	Mannitol-triacetate-3-acetyloxy-C18	PEFA	3	[9-11]
	653.3890	15.13	**593.37**; **533.35**; **473.33**; 451.34; 413.30; 393.30; 293.28; **283.08**; 223.06; 201.08	[M+Na]+	$C_{31}H_{54}O_{11}$	Arabitol-triacetate-3-acetyloxy-C20	PEFA	3	
	627.6426	12.82	**567.32**; **507.29**; **447.27**; **387.25**; 379.25; 337.24; **313.09**; 253.07; 237.22; 231.09; 189.08; 111.05	[M+Na]+	$C_{30}H_{52}O_{12}$	Mannitol-triacetate-3-acetyloxy-C16	PEFA	3	[10]
	625.3367	14.40	**565.34**; **505.31**; **445.29**; 423.31; **385.25**; 365.27; **283.08**; 265.25; 223.06; 202.08;	[M+Na]+	$C_{31}H_{54}O_{11}$	Arabitol-triacetate-3-acetyloxy-C18	PEFA	3	[9,10]
	599.4146	11.65	**539.28**; **479.26**; **419.24**; 397.26; **359.22**; 351.21; **313.09**; 253.07; 231.09; 209.19; 189.08; 153.06; 111.05	[M+Na]+	$C_{28}H_{48}O_{12}$	Mannitol-triacetate-3-acetyloxy-C14	PEFA	3	[11]
	597.3263	13.46	**537.30**; **477.28**; **417.26**; 395.28; **357.24**; 337.24; **283.08**; 255.11; 237.22; 223.06201.08	[M+Na]+	$C_{29}H_{50}O_{11}$	Arabitol-triacetate-3-acetyloxy-C16	PEFA	3	[9,10]
	571.4611	10.55	**511.25**; **451.23**; **391.21**; 396.23; **331.19**; **313.08**; 281.17; 253.07; 231.09; 189.08; 181.16; 153.06; 111.05	[M+Na]+	$C_{26}H_{44}O_{12}$	Mannitol-triacetate-3-acetyloxy-C12	PEFA	3	
	569.3000	12.30	**509.27**; **449.25**; **389.23**; 367.25; **329.21**; 309.20; **283.08**; 255.11; 223.06; 209.19	[M+Na]+	$C_{27}H_{46}O_{11}$	Arabitol-triacetate-3-acetyloxy-C14	PEFA	3	
	541.2764	10.93	**481.24**; **421.22**; **361.20**; 339.22; **301.18**; **283.08**; 223.06; 201.08; 181.16; 141.06	[M+Na]+	$C_{25}H_{42}O_{11}$	Arabitol-triacetate-3-acetyloxy-C12	PEFA	3	

	655.3052	14.16	**595.35**; **535.33; 475.30**; 453.32; **415.28**; 365.27; **313.09**; 265.25; 253.07; 231.09; 189.08; 153.06; 111.05	$[M+Na]^+$	$C_{32}H_{56}O_{12}$	Mannitol-triacetate-3-acetyloxy-C18	PEFA	2	
**1**	627.3359	12.84	**567.32**; **507.29**; **447.27**; 425.29; **387.25**; 337.24; **313.09**; 253.07; 231.09; 189.08; 111.05	$[M+Na]^+$	$C_{30}H_{52}O_{12}$	Mannitol-triacetate-3-acetyloxy-C16	PEFA	2	
	599.3064	11.65	**539.28**; **479.26**; **419.24**; 397.26; **359.22**; 355.21; **313.09**; 309.20; 253.07; 231.09; 209.19; 189.08; 153.06; 111.05	$[M+Na]^+$	$C_{28}H_{48}O_{12}$	Mannitol-triacetate-3-acetyloxy-C14	PEFA	2	
	571.2739	10.59	**511.25**; **451.23**; 395.20; **391.21**; 369.23; ***331.13***; **313.09**; 281.17; 253.07; 231.09; 197.04; 189.08; 171.01; 153.06; 111.05	$[M+Na]^+$	$C_{26}H_{44}O_{12}$	Mannitol-triacetate-3-acetyloxy-C12	PEFA	2	
	639.3936	14.00	**579.32**; **519.29**; 497.31; 477.28; **459.27**; 437.29; **399.31**; 395.28; 349.29; 337.24; **325.09**; 297.12; 283.26; 265.07; 237.22; 210.08; 141.06	$[M+Na]^+$	$C_{33}H_{56}O_{12}$	Arabitol-tetraacetate-3-acetyloxy-C16	PEFA	3	[9,10]
**4**	667.4842	14.95	**607.35**; **547.33**; **525.34**; **505.31**; **487.30**; **465.31**; **445.29**; 427.28; 423.31; 365.27; **325.09**; 303.11; 265.25; 247.24; 210.08; 141.06; 81.03	$[M+Na]^+$	$C_{31}H_{52}O_{12}$	Arabitol-tetraacetate-3-acetyloxy-C18	PEFA	3	[9,10]
	459.2210	7.46	**399.20**; **339.18**; 253.14; **229.07**; 189.08; 169.05; 154.13; 129.06	$[M+Na]^+$	$C_{20}H_{36}O_{10}$	Mannitol-monoacetate-3-acetyloxy-C10	PEFA	2	
	487.2520	8.85	**427.23**; **367.21**; 281.17; **229.07**; 189.08; 181.16; 169.05; 129.06; 111.05	$[M+Na]^+$	$C_{22}H_{40}O_{10}$	Mannitol-monoacetate-3-acetyloxy-C12	PEFA	2	
	501.2312	8.39	**441.21**; **381.19**; **321.17**; **271.08**; 253.14; 211.06; 189.08; 153.13; 129.06; 111.05	$[M+Na]^+$	$C_{22}H_{38}O_{11}$	Mannitol-diacetate-3-acetyloxy-C10	PEFA	2	
	529.2628	9.56	**469.24**; **409.22**; 353.19; **349.20**; 323.16; 281.17; **271.08**; 211.06; 189.08; 181.16; 111.05	$[M+Na]^+$	$C_{24}H_{42}O_{11}$	Mannitol-diacetate-3-acetyloxy-C12	PEFA	2	

	585.3253	12.33	**525.30**; **465.28**; 409.26; **405.26**; 379.25; 337.24; **271.08**; 236.22; 211.06; 189.08; 111.05	$[M+Na]^+$	$C_{28}H_{50}O_{11}$	Mannitol-diacetate-3-acetyloxy-C16	PEFA	2	[11]
	583.2775	12.28	**523.25**; **463.23**; 441.25; 421; **403.21**; 381.23; **343.19**; 339.22; **325.09**; 281.17; 256.07; 201.08; 181.16; 141.06	$[M+Na]^+$	$C_{28}H_{48}O_{11}$	Arabitol-tetraacetate-3-methoxy-C14	PEFA	3	
	655.2927	11.14	**595.31**; **535.29**; **513.30**; **475.27**; **453.29**; 433.26; **415.26**; **393.27**; 353.23; 325.09; 293.21; 265.07; 235,21; 201.08; 141.06	$[M+Na]^+$	$C_{32}H_{52}O_{14}$	Mannitol-pentaacetate-3-acetyloxy-C12	PEFA	3	
	683.3435	12.51	**623.34**; **563.32**; **541.34**; 521.31; **503.30**; **443.27**; **481.31**; 461.29; **421.28**; 381.26; 325.09; 321.24; 303.11; 265.07; 201.08; 141.06	$[M+Na]^+$	$C_{30}H_{48}O_{14}$	Mannitol-pentaacetate-3-acetyloxy-C14	PEFA	3	[10]
	611.3049	10.68	**551.28**; **491.26**; **431.24**; 369.12; 351.21; 309.09; 291.19; **283.08**; 223.06; 210.08; 141.05	$[M+Na]^+$	$C_{31}H_{56}O_{10}$	Arabitol-triacetate-3-hydroxy-C20	PEFA	3	
	653.3145	12.27	**593.29**; **533.27**; 511.29; 491.26; **473.25**; 432.24; 351.21; **325.09**; 291.19; 265.07; 223.21; 201.08; 155.14; 141.06	$[M+Na]^+$	$C_{33}H_{58}O_{11}$	Arabitolpentaacetate-3-hydroxy-C20	PEFA	3	

* For PEFA annotations, fragments most indicative of loss of acetyl groups and the residual polyol moiety are highlighted in bold

Table S6. Theoretical masses of acetylated PEFAs

	Mannitol 0 acetyl hydroxyl	Mannitol 1 acetyl hydroxyl	Mannitol 2 acetyl hydroxyl	Mannitol 3 acetyl hydroxyl	Mannitol 4 acetyl hydroxyl	Mannitol 5 acetyl hydroxyl
C14	431	473	515	557	599	641
C16	459	501	543	585	627	669
C18	487	529	571	613	655	697
C20	515	557	599	641	683	725
	Mannitol 0 acetyl methoxy	Mannitol 1 acetyl methoxy	Mannitol 2 acetyl methoxy	Mannitol 3 acetyl methoxy	Mannitol 4 acetyl methoxy	Mannitol 5 acetyl methoxy
C14	445	487	529	571	613	655
C16	473	515	557	599	641	683
C18	501	543	585	627	669	711
C20	529	571	613	655	697	739
	Mannitol 0 acetyl acetoxy	Mannitol 1 acetyl acetoxy	Mannitol 2 acetyl acetoxy	Mannitol 3 acetyl acetoxy	Mannitol 4 acetyl acetoxy	Mannitol 5 acetyl acetoxy
C14	473	515	557	599	641	683
C16	501	543	585	627	669	711
C18	529	571	613	655	697	739
C20	557	599	641	683	725	767
	Arabitol 0 acetyl hydroxyl	Arabitol 1 acetyl hydroxyl	Arabitol 2 acetyl hydroxyl	Arabitol 3 acetyl hydroxyl	Arabitol 4 acetyl hydroxyl	
C14	401	443	485	527	569	
C16	429	471	513	555	597	
C18	457	499	541	583	625	
C20	485	527	569	611	653	
	Arabitol 0 acetyl methoxy	Arabitol 1 acetyl methoxy	Arabitol 2 acetyl methoxy	Arabitol 3 acetyl methoxy	Arabitol 4 acetyl methoxy	
C14	415	457	499	541	583	
C16	443	485	527	569	611	
C18	471	513	555	597	639	
C20	499	541	583	625	667	
	Arabitol 0 acetyl acetoxy	Arabitol 1 acetyl acetoxy	Arabitol 2 acetyl acetoxy	Arabitol 3 acetyl acetoxy	Arabitol 4 acetyl acetoxy	
C14	443	485	527	569	611	
C16	471	513	555	597	639	
C18	499	541	583	625	667	
C20	527	569	611	653	695	

Table S7. MS/MS fragment ions indicative of the type of polyol groupp in PEFAs

	m/z $[M+H]^+$	*m/z* $[M+NH_4]^+$	*m/z* $[M+Na]^+$
Mannitol, 0 Ac	165.1	182.1	187.1
Mannitol, 1 Ac	207.1	224.1	229.1
Mannitol, 2 Ac	249.1	266.1	271.1
Mannitol, 3 Ac	291.1	308.1	313.1
Mannitol, 4 Ac	333.1	350.1	355.1
Mannitol, 5 Ac	375.1	392.1	397.1
Arabitol, 0 Ac	135.1	152.1	157.1
Arabitol, 1 Ac	177.1	194.1	199.1
Arabitol, 2 Ac	219.1	236.1	241.1
Arabitol, 3 Ac	261.1	278.1	283.1
Arabitol, 4 Ac	303.1	320.1	325.1

Table S8. NMR data for compound **1** (MeOD, 600/150 MHz)

Position	δ_H Multiplicity (J in Hz)	δ_C	COSY	HMBC H → C	TOCSY	NOE
1	3.63 (m), 3.80 (m)	64.8	H-2	C-3	H-6	
2	3.79 (m)	70.3	H-1, H-3	C-1, C-3, C-4, C-11	H-1, H-3, H-4, H-5, H-6	H-5
3	3.69 (m)	72.7	H-2, H-4	C-9	H-2	
4	3.48 (m)	70.4	H-3, H-5	C-5	H-2, H-5, H-6	H-5
5	3.87 (m)	70.0	H-4, H-6	C-4	H-2, H-4, H-6	H-2, H-4, H-6
6	4.16 (m), 4.37 (m)	67.7	H-5	C-4, C-7	H-2, H-4, H-5, H-6	H-5
7	-	172.1				
8	2.05 (s)	20.3 - 20.8		C-7		
9	-	173.1				
10	2.03 (s)	20.3 - 20.8		C-9		
11	-	172.9				
12	2.08 (s)	20.6		C-11		
1'	-	172.3				
2'	2.61 (m), 2.65 (m)	39.8	H-3'	C-1', C-3', C-4'	H-3', H-4', H-5'	H-4'
3'	5.22 (m)	71.8	H-2', H-4'	C-1', C-2', C-4', C-5', C-17'	H-2', H-4', H-5'	H-2', H-4', H-5'
4'	1.61 (m), 1.61 (m)	34.7	H-3', H-5'			H-2', H-3', H-5'
5'	1.33 (m)	25.9	H-4'			
6' - 13'	1.29 – 1.33 (m)	30.2 – 31.0				
14'	1.29 (m)	32.9	H-13', H-15'			
15'	1.31 (m)	23.5	H-14', H-16'	C-14', C-16'		
16'	0.90 (t, 6.9)	14.1	H-15'	C-14', C-15'		
17'	-	172.3				
18'	2.02 (s)	20.8		C-17'		

Table S9. NMR data for compound **2** (MeOD, 600/150 MHz)

Position	δ_H Multiplicity (J in Hz)	δ_C	COSY	HMBC H → C	NOE
1	3.63 (m), 3.80 (m)	64.7	H-2	C-2	H-2
2	3.79 (m)	70.3	H-1, H-3	C-3	H-1, H-3, H-5
3	3.69 (m)	72.5	H-2, H-4		H-2, H-4
4	3.47 (m)	70.7	H-3, H-5	C-7	H-3, H-5
5	3.87 (m)	70.0	H-4, H-6	C-4	H-2, H-4, H-6
6	4.18 (m), 4.39 (m)	67.8	H-5		H-5
7		172.9			
8	2.08 (s)	20.5		C-7	
1'		172.3			
2'	2.65 (m), 2.65 (m)	39.8	H-3'	C-1', C-3', C-4'	H-4'
3'	5.22 (m)	71.7	H-2', H-4'	C-1', C-17'	H-2', H-4', H-5'
4'	1.62 (m), 1.62 (m)	34.8	H-3', H-5'	C-5'	H-2', H-3', H-5'
5'	1.32 (m)	25.9	H-4'		
6' - 13'	1.29 – 1.33 (m)	30.2 – 31.0			
14'	1.29 (m)	32.8	H-13', H-15'		
15'	1.31 (m)	23.5	H-14', H-16'	C-14', C-16'	
16'	0.90 (t, 6.9)	14.2	H-15'	C-15'	
17'		172.3			
18'	2.02 (s)	20.8		C-17'	

Table S10. NMR data of compound **5** (MeOD, 600/150 MHz)

**Position**	***δ*H m (*J* in ppm)**	***δ*C**	**HMBC** C→ H	**NOE**
1		175.7	H-1, H_2-3, OMe	
2	4.43 (dd, 6.9, 5.4)	72.6	H_2-3	H_2-3
3	3.11 (ddd, 14.5, 6.5, 0.7)<br>3.21 (ddd, 14.5, 5.4, 0.7)	31.3	H-2<br>H-2	H-2, H-2', H-4'
1'		137.7	H-4', H-6'	
2'	7.09 (s)	124.5	H_2-3	H-2, H_2-3, OMe
3'		110.8	H_2-3, H-2, H-4', H-2'	
3a'		127.3	H2-3, H-2', H-7'	
4'	7.53 (d, 7.8)	119.1	H-6'	H-2, H_2-3, H-5', OMe
5'	6.99 (t, 7.8)	119.4	H-7'	H-4', H-6'
6'	7.07 (t, 7.8)	122.0	H-4'	H-5', H-7'
7'	7.31 (d)	111.9	H-5'	H-6'
7a'		110.1	H-4, H-2, H-6'	
OMe	3.64 (s)	52.0		H-2', H-4'

Figure S1. Differential metabolomes of *R. mucilaginosa* 50-3-19/20B (a) on PDA medium; (b) on WSP30 medium

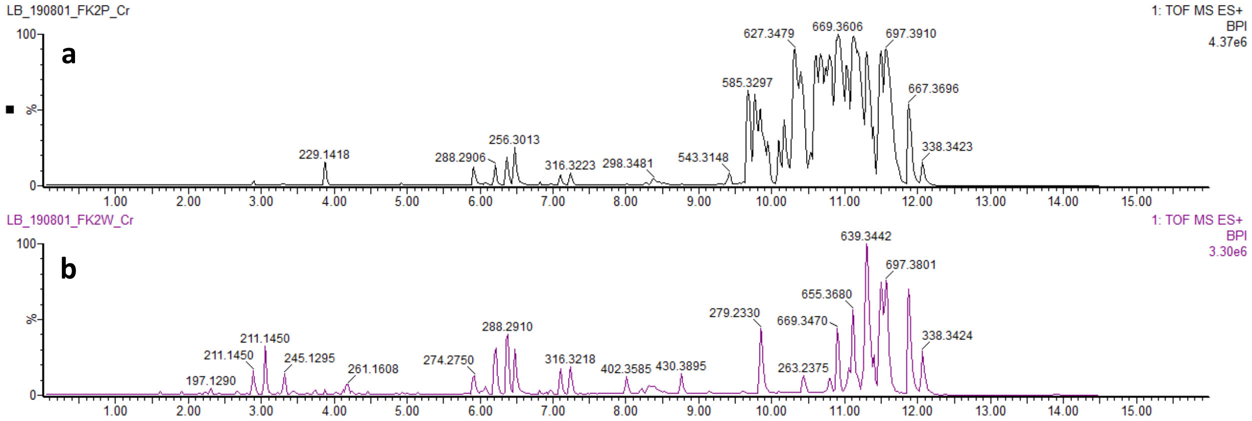

Figure S2. Overview of genomic statistics based on *de novo* genome assembly. (A) Genome Assembly statistics (B) Total repetitive elements

A

	denovo Assembly	Definition
**Assembled Size (Mb)**	20.02	Total denovo assembled size
**Total scaffolds**	265	Total number of scaffolds in assembled genome
**N25 (Kb)**	498.8	Length of the scaffold until which sum of lengths of scaffolds are reached to 25% of 20.02 Mb
**L25**	8	Number of scaffolds in the assembled genome that constitute N25
**N50 (Kb)**	295.8	Length of the scaffold until which sum of lengths of scaffolds are reached to 50% of 20.02 Mb
**L50**	21	Number of scaffolds in the assembled genome that constitute N50
**N75 (Kb)**	163.5	Length of the scaffold until which sum of lengths of scaffolds are reached to 75% of 20.02 Mb
**L75**	44	Number of scaffolds in the assembled genome that constitute N75
**%GC**	60.47	Percentage of total G+C content of assembled genome (20.02 Mb)

B

Types of repeats	No. of Repeats*	Total length (bp)	Percentage of genomic sequence
**Retroelements**	**81**	**55667**	**0.28**
LINEs	18	3607	0.02
LTR elements	**63**	**52060**	**0.26**
Ty1/Copia	28	30103	0.15
Gypsy/DIRS1	33	21758	0.11
**DNA transposons**	**9**	**581**	**0.00**
Tc1-IS630-Pogo	1	15	0.00
Unclassified	2	887	0.00
**Small RNA**	**10**	**7904**	**0.04**
**Simple repeats**	**8318**	**326295**	**1.63**
**Low complexity**	**759**	**37594**	**0.19**
Total interspersed repeats		57135	0.29
**Total repeat contents**		**428912**	**2.14**

Figure S3. GNPS MS/MS mirror plot of experimental and library data of 9-oxo-10*E*,12*Z*-octadecadienoic acid

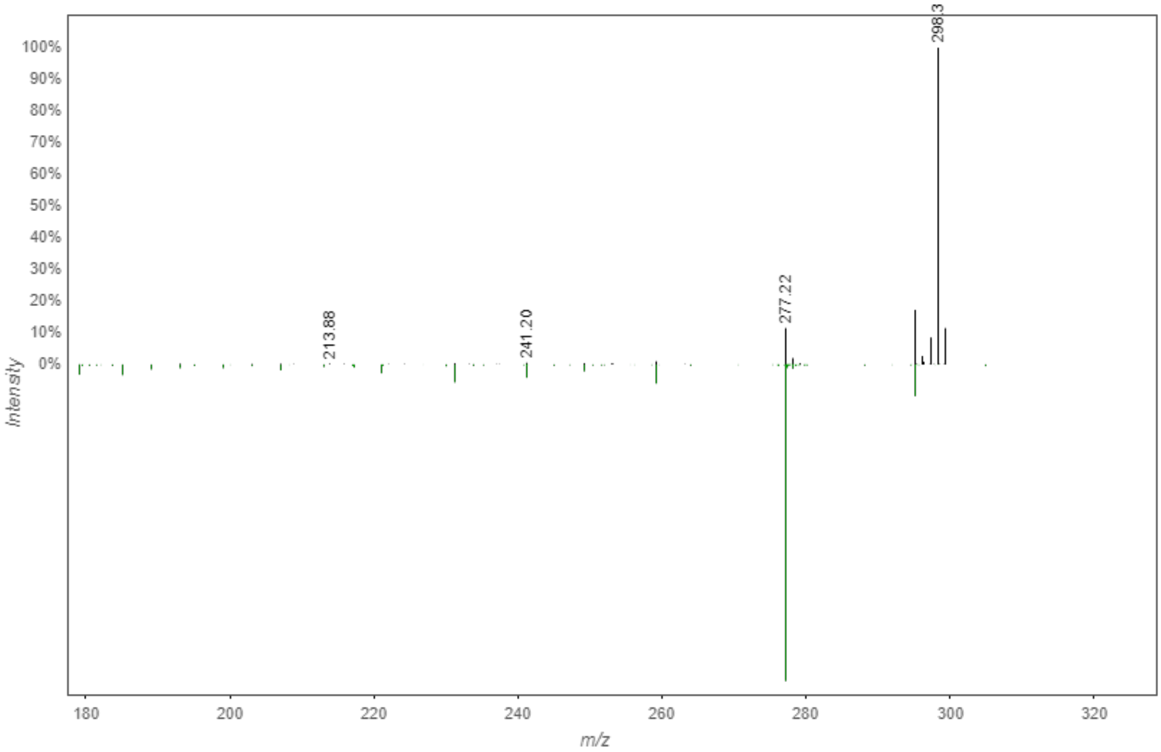

Figure S4. GNPS MS/MS mirror plot of experimental and library data of cyclo-(Leu-Phe)

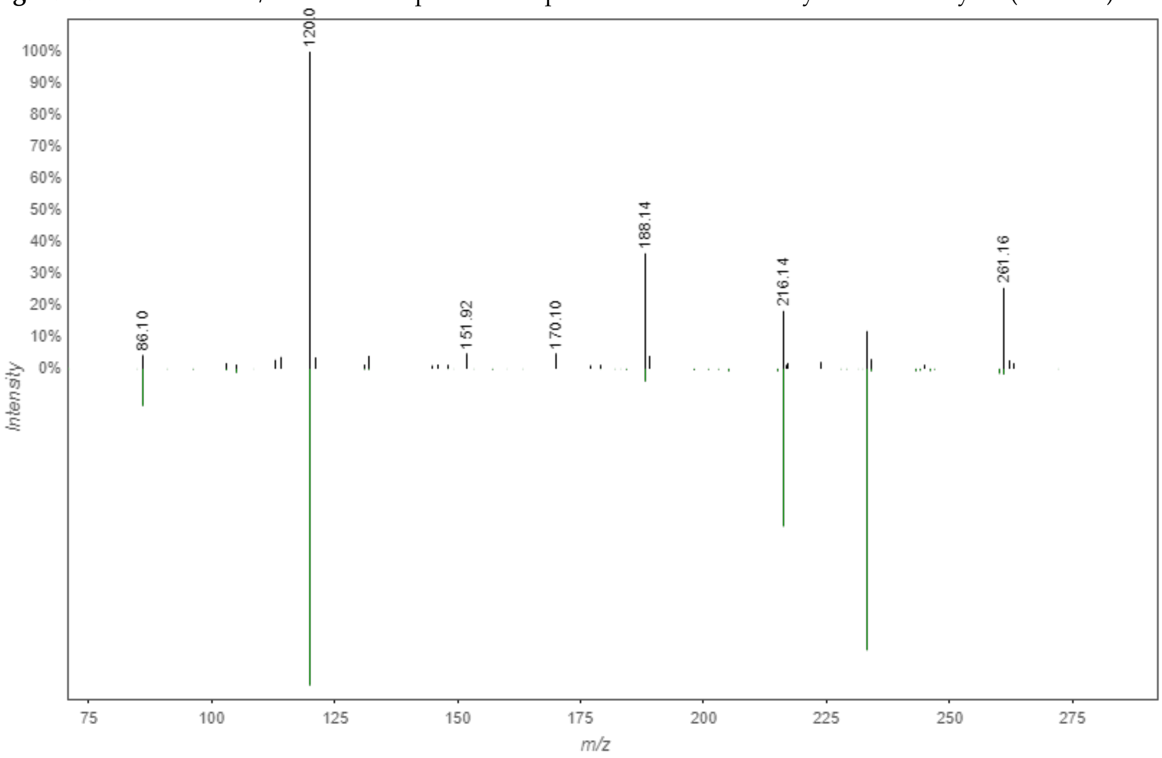

Figure S5. GNPS MS/MS mirror plot of experimental and library data of DL-Indole-3-lactic acid

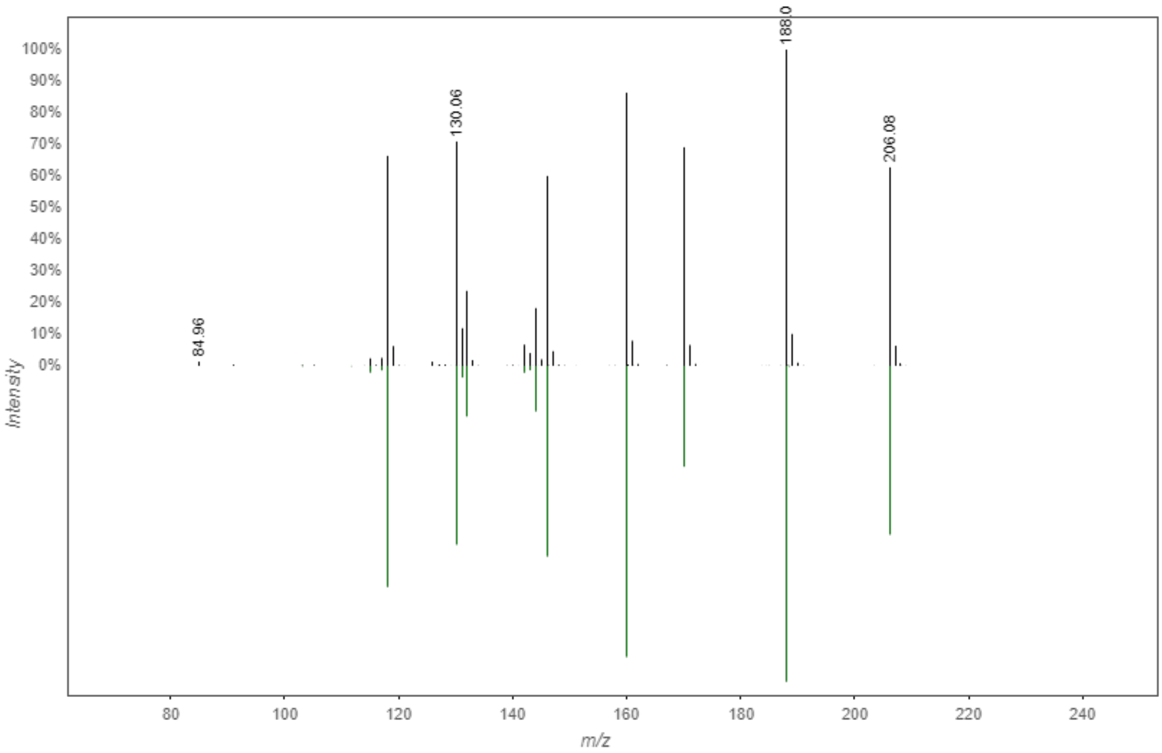

Figure S6. GNPS MS/MS mirror plot of experimental and library data of 5α,8α-epidioxyergosta-6,22-dien-3β-ol

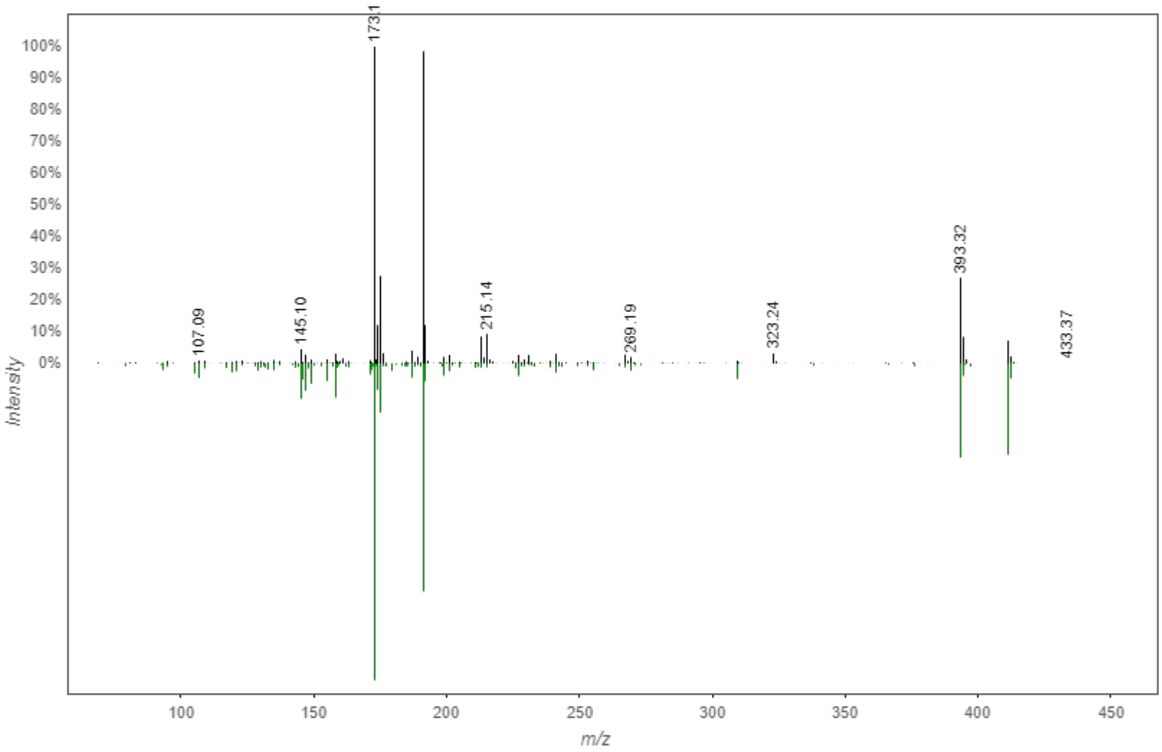

Figure S7. GNPS MS/MS mirror plot of experimental and library data of C17-sphinganine

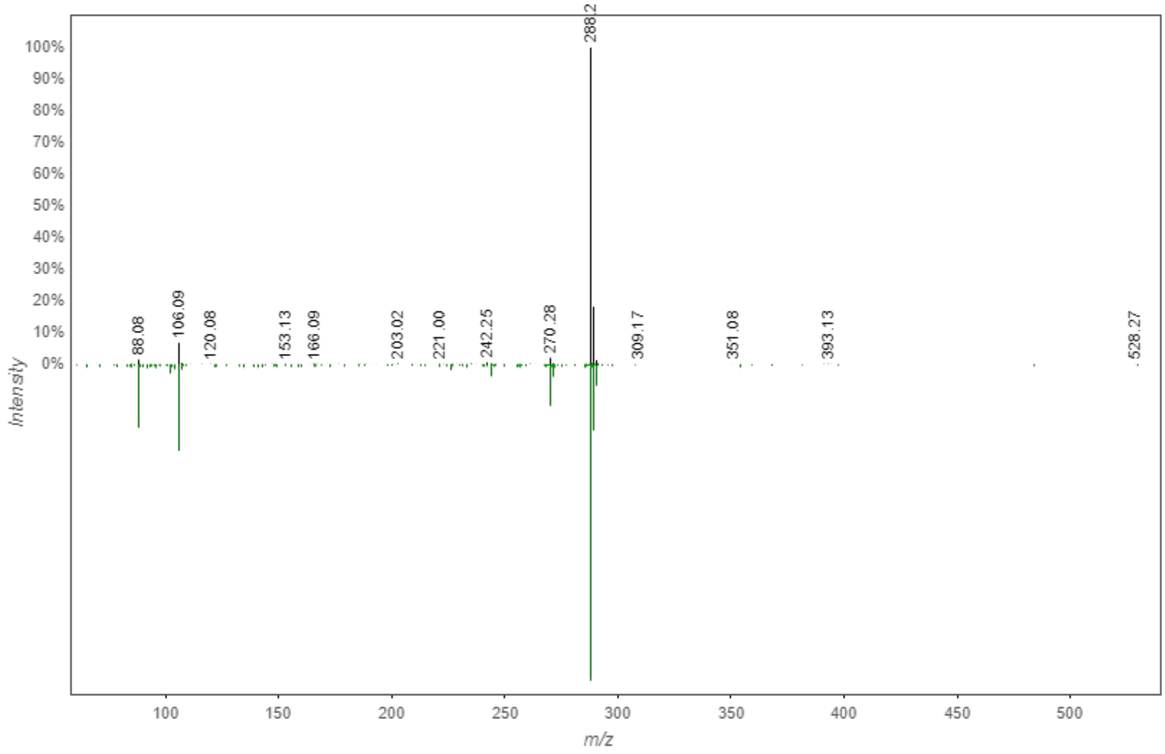

Figure S8. GNPS MS/MS mirror plot of experimental and library data of 9,10-Epoxy-12-octadecenoic acid

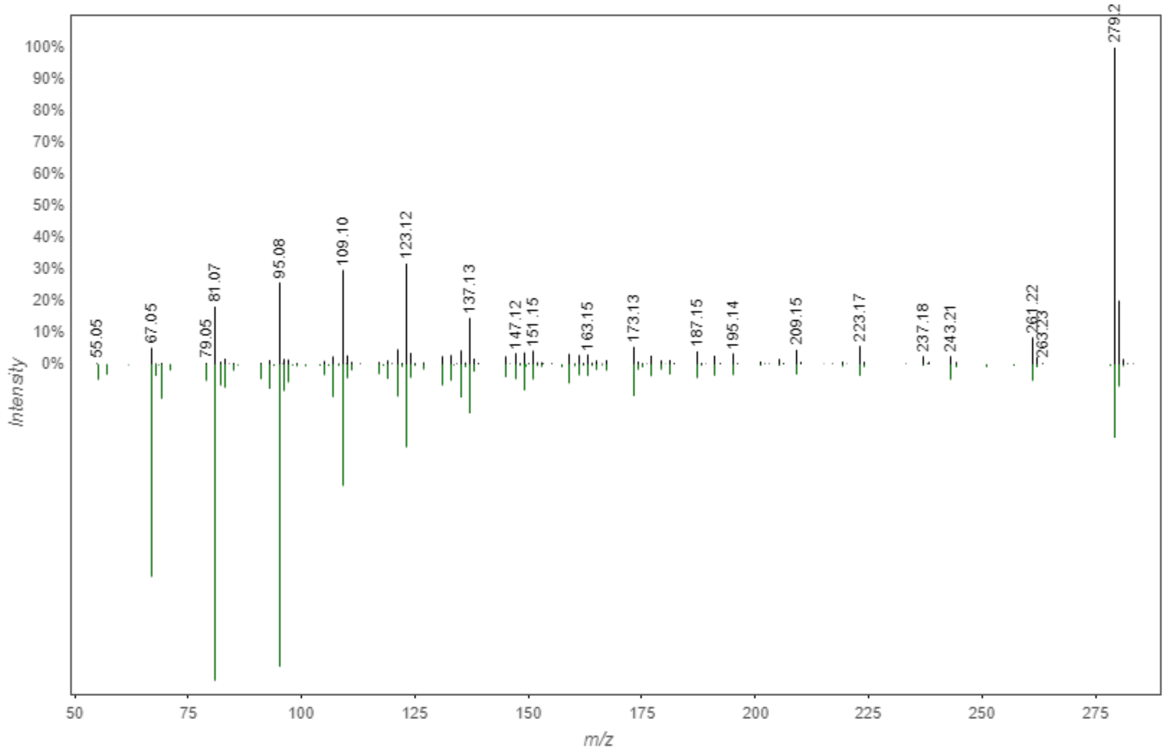

Figure S9. (a) HR-MS and (b) MS/MS spectra of **1**

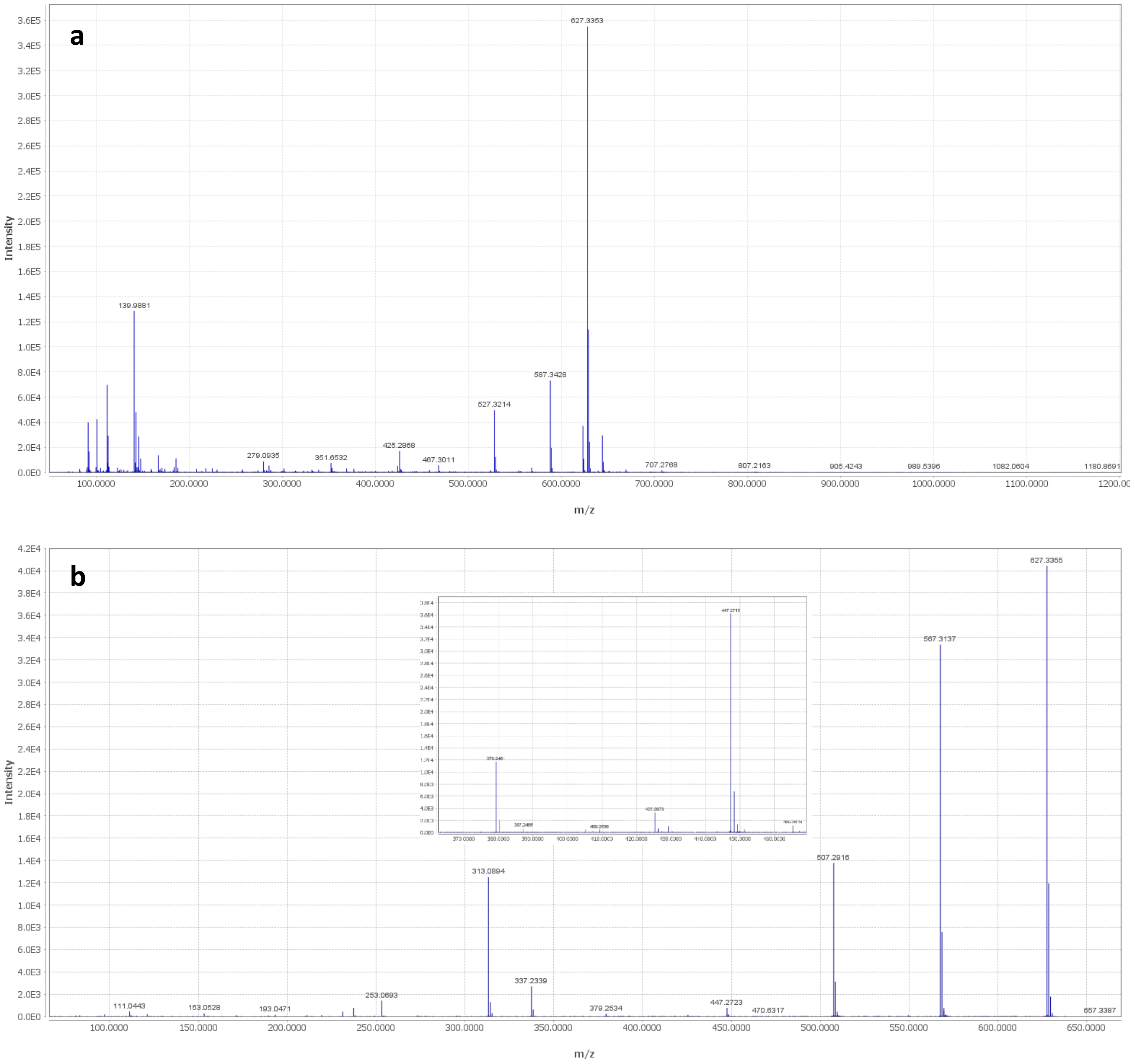

Figure S10. ^{1}H NMR spectrum of compound **1** (MeOD, 600 MHz)

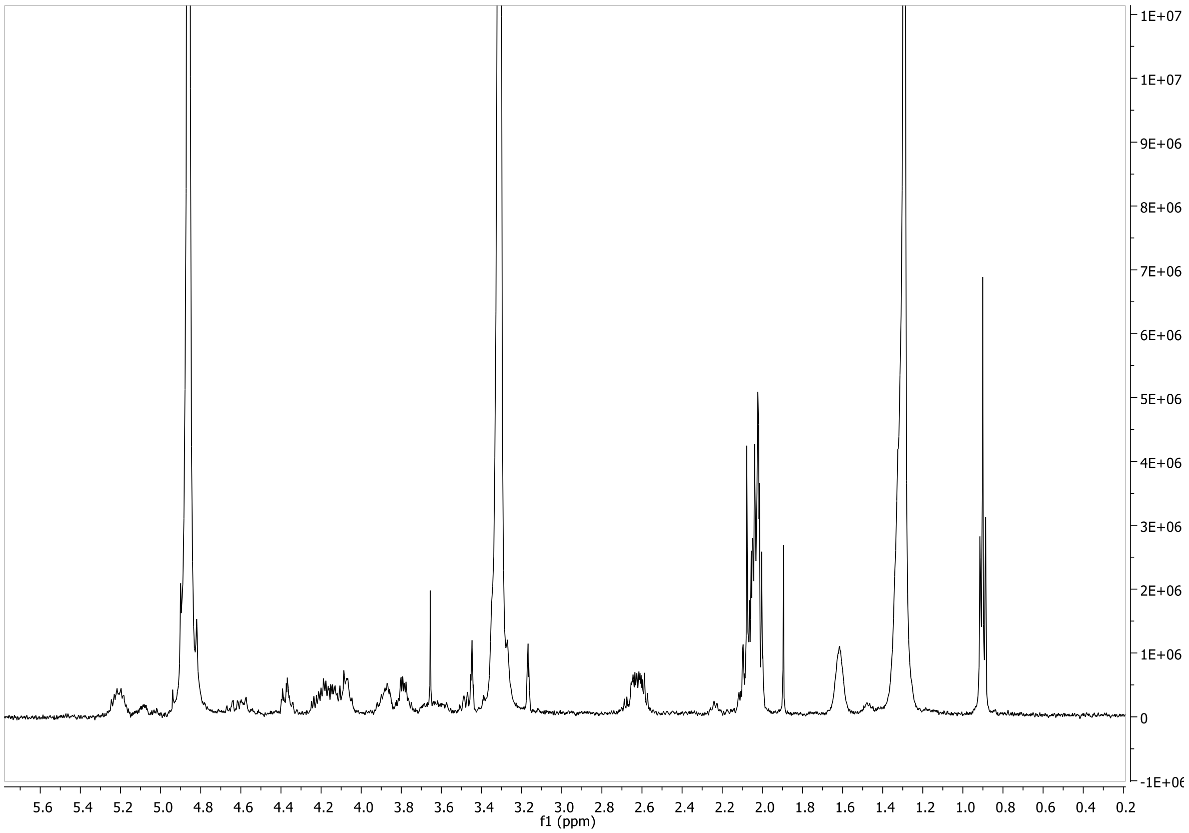

Figure S11. HSQC spectrum of compound **1** (MeOD, 600/150 MHz)

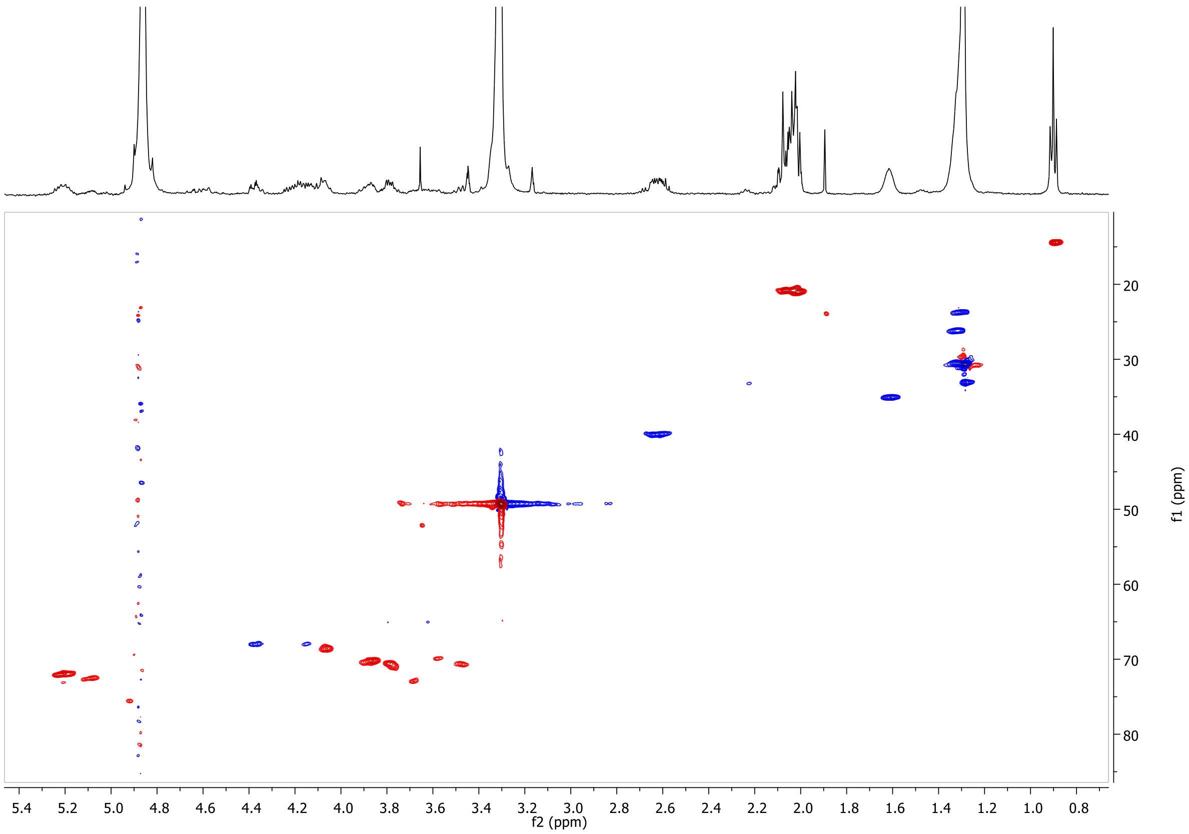

Figure S12. COSY spectrum of compound **1** (MeOD, 600 MHz)

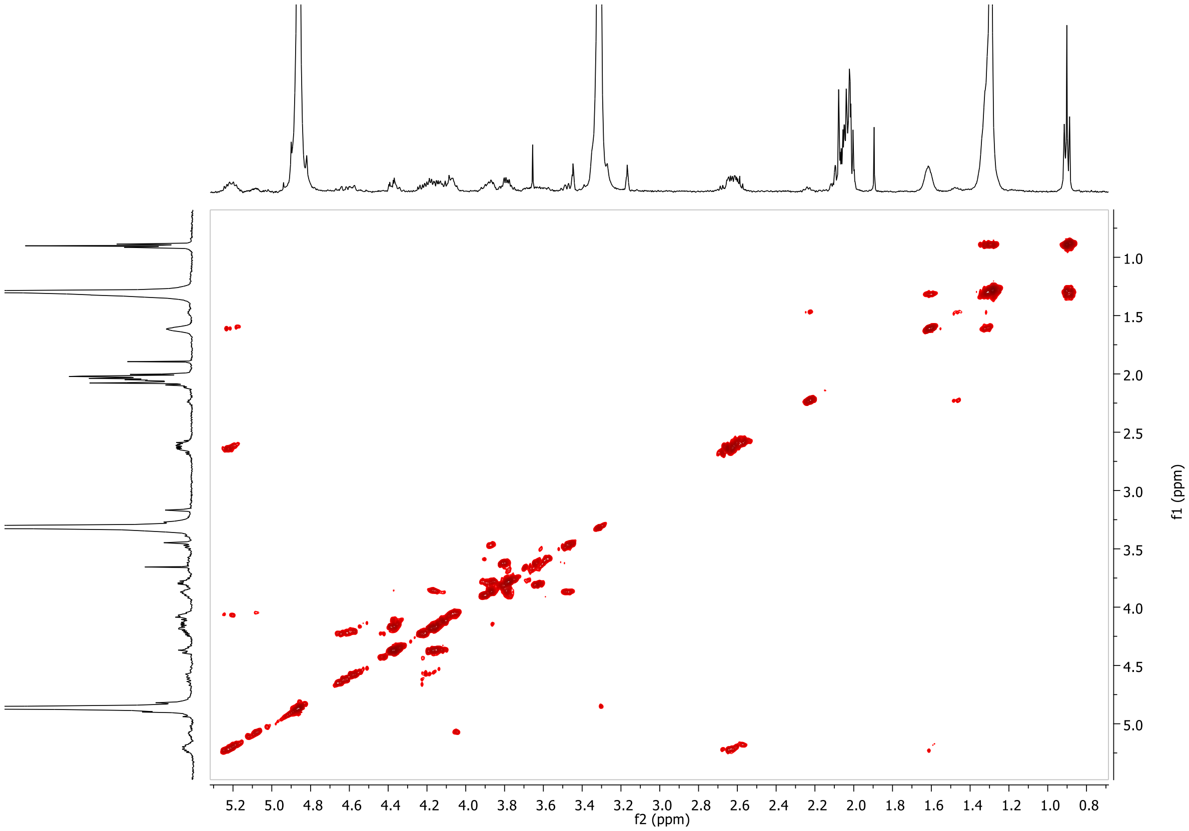

Figure S13. HMBC spectrum of compound **1** (MeOD, 600/150 MHz)

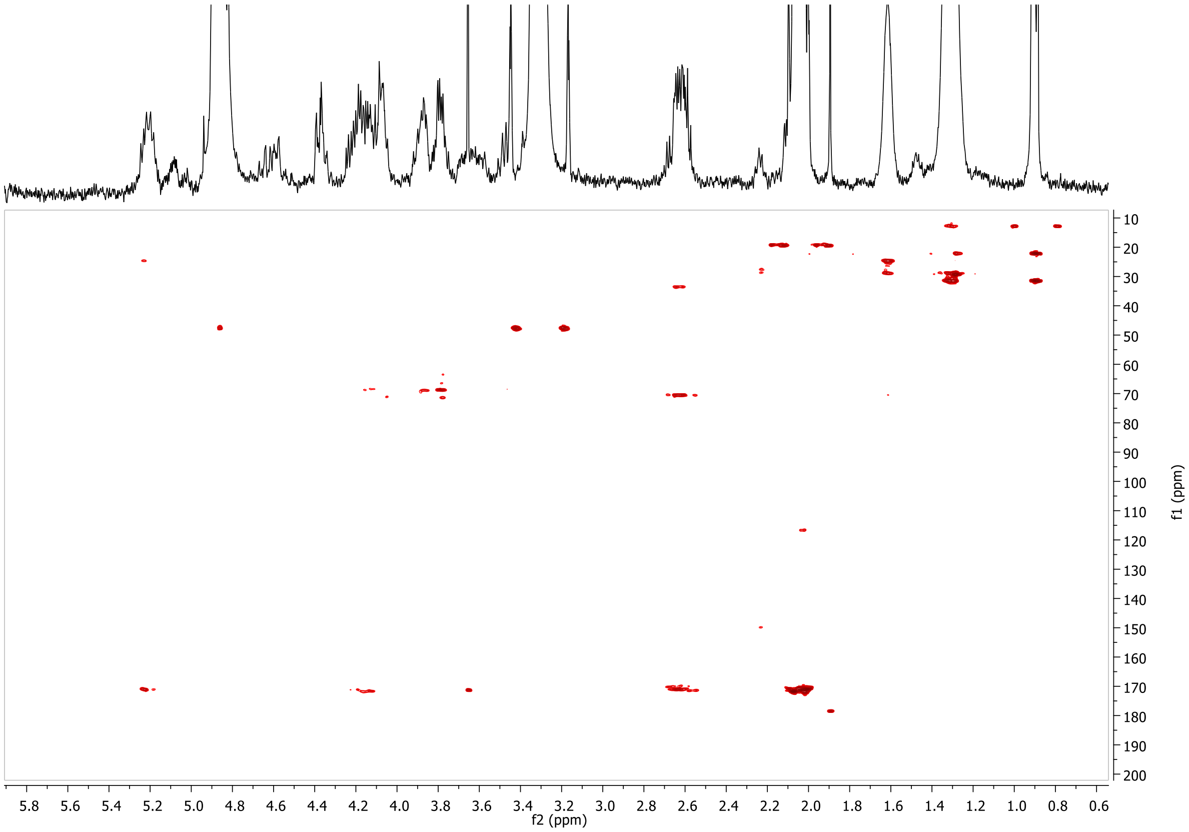

Figure S14. NOESY spectrum of compound **1** (MeOD, 600 MHz)

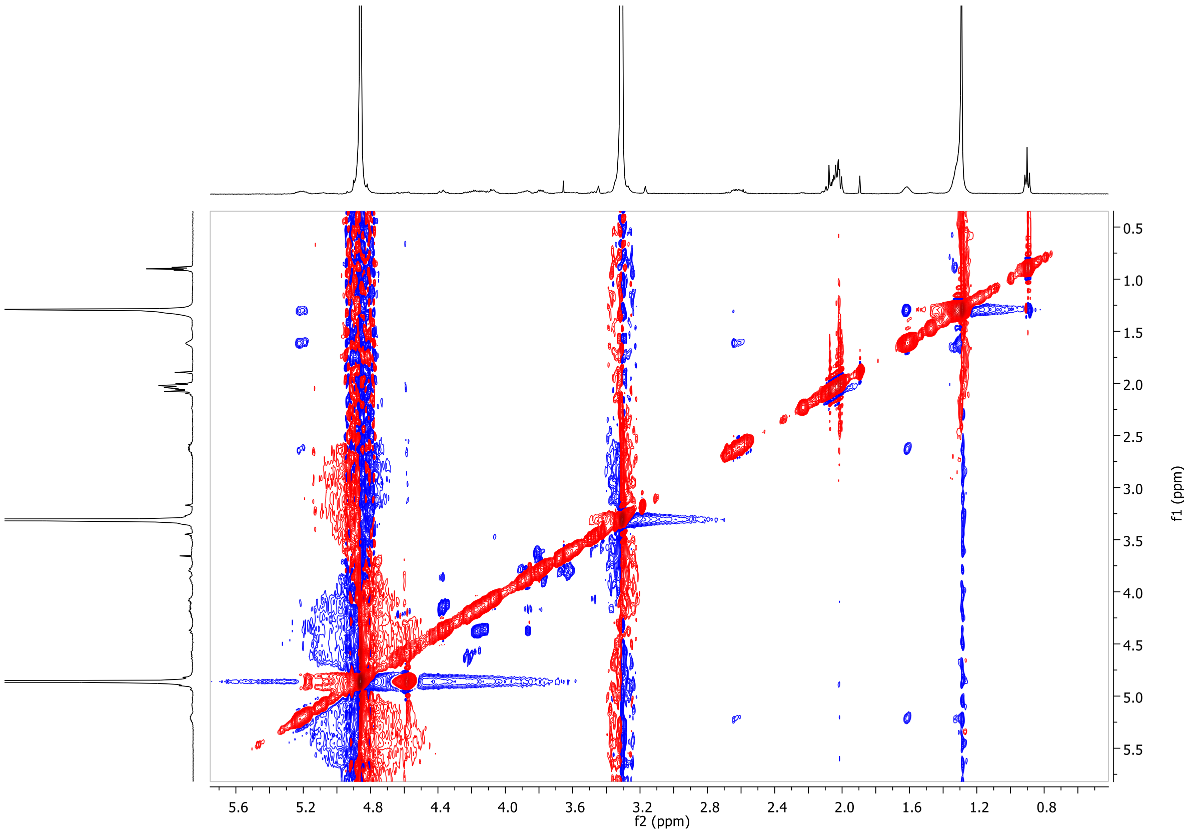

Figure S15. TOCSY spectrum of compound **1** (MeOD, 600 MHz)

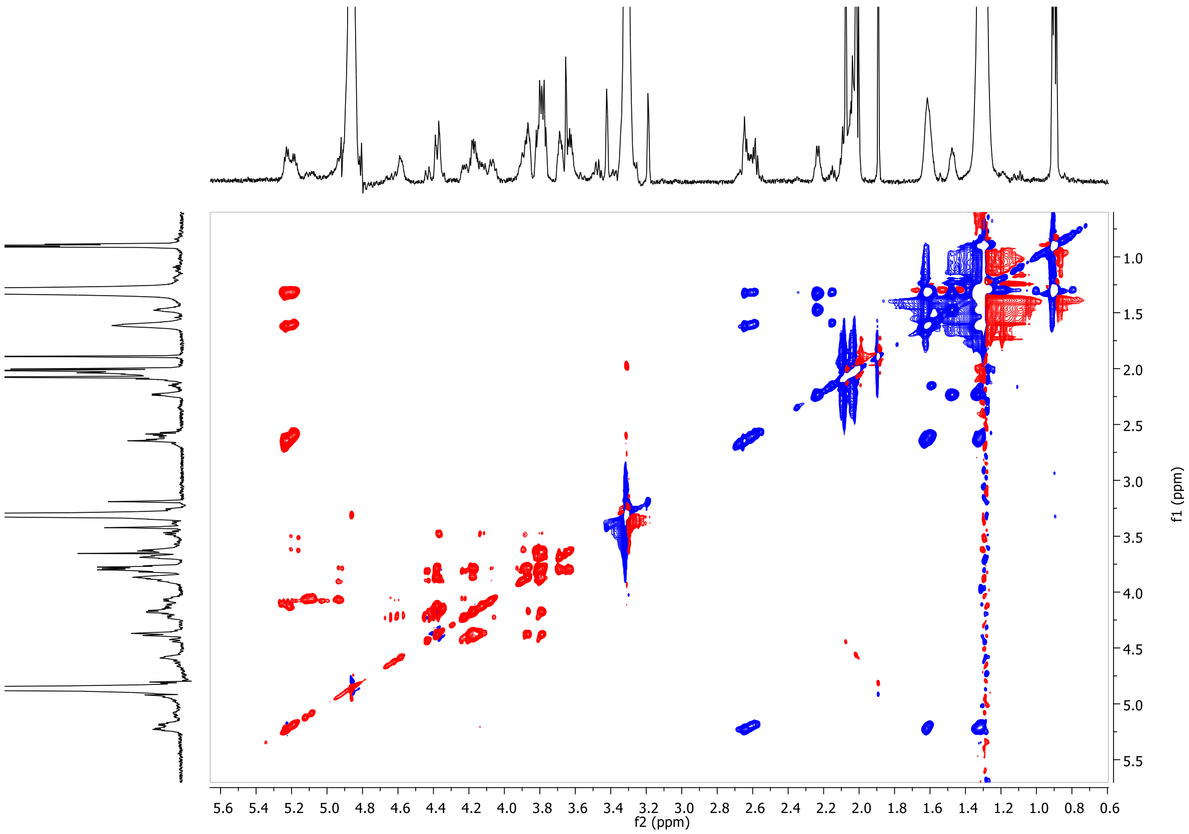

Figure S16. (a) HR-ESIMS and (b) MS/MS spectra of **2**

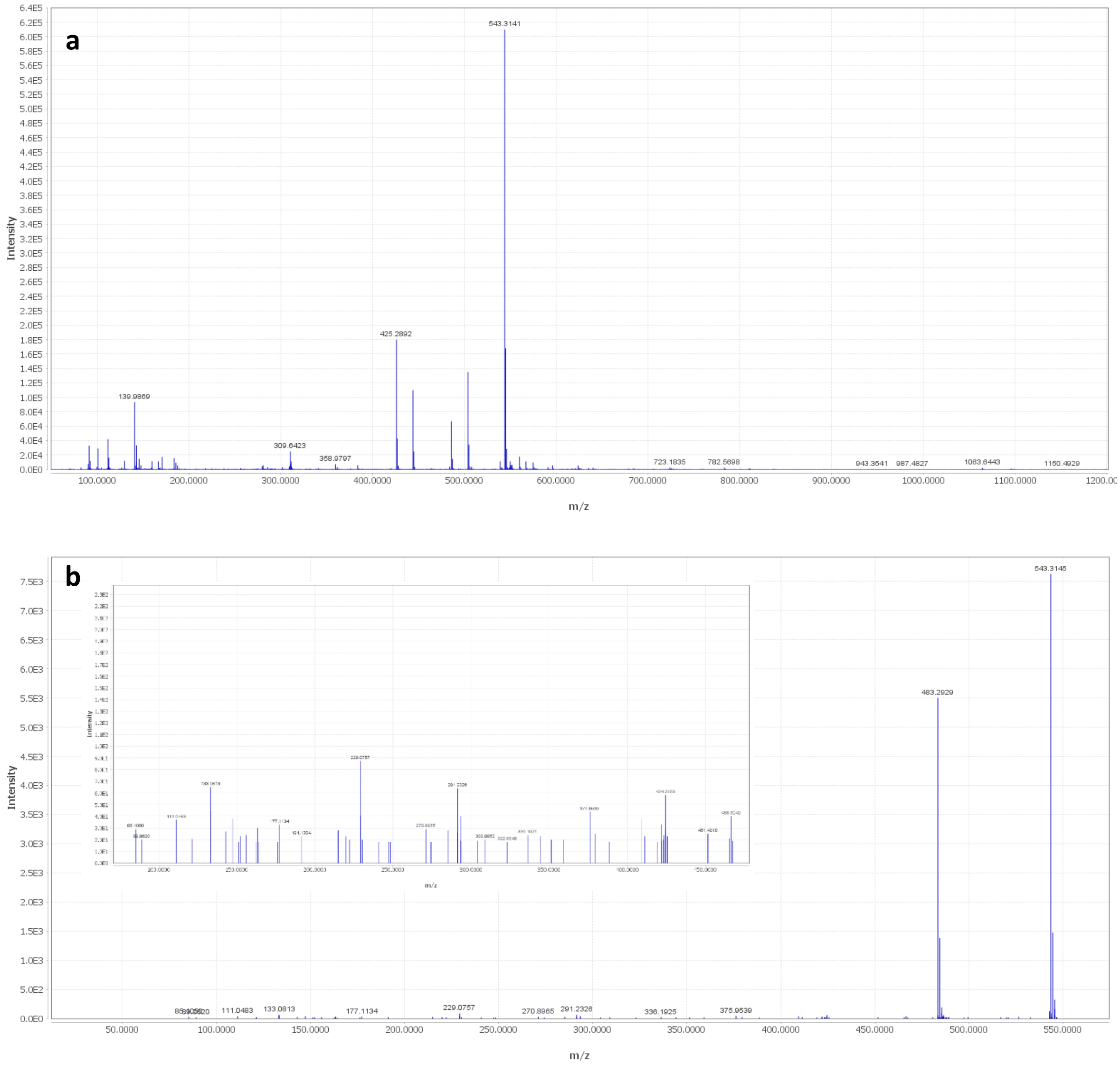

Figure S17. ^{1}H NMR spectrum of compound **2** (MeOD, 600 MHz)

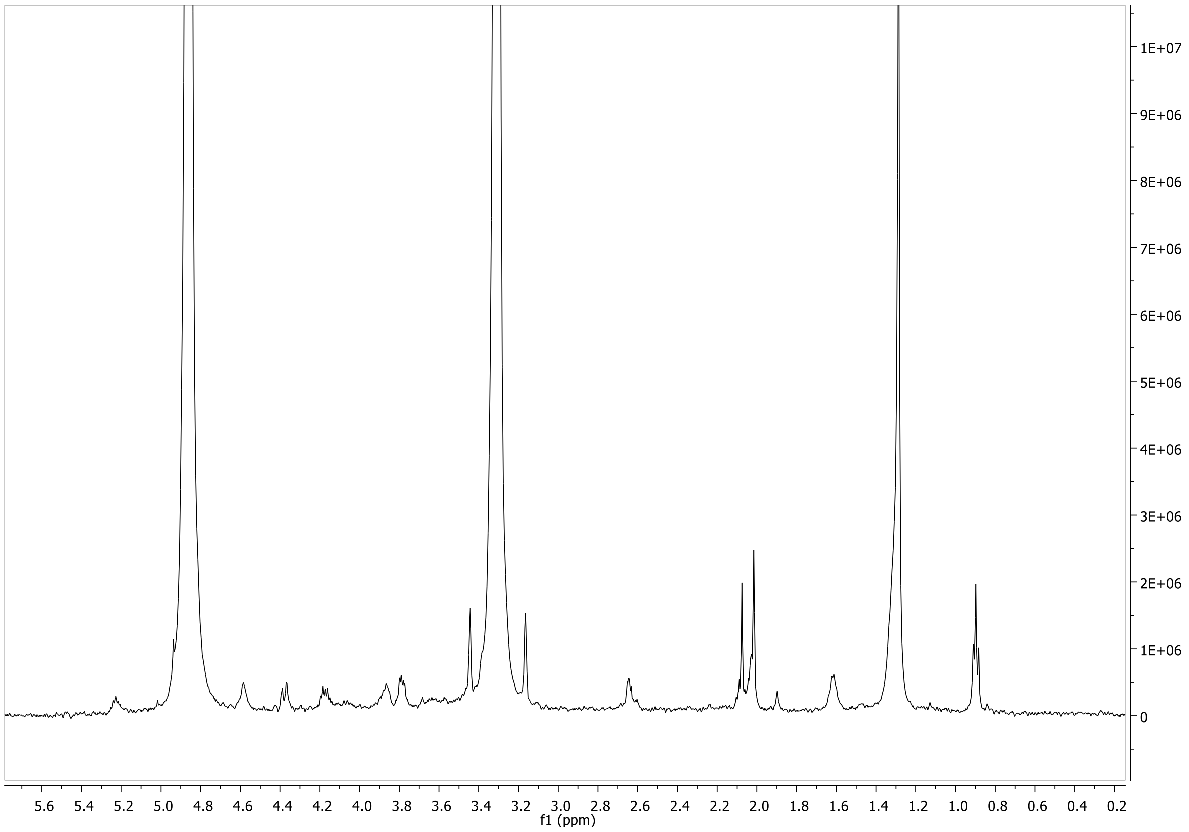

Figure S18. HSQC spectrum of compound **2** (MeOD, 600/150 MHz)

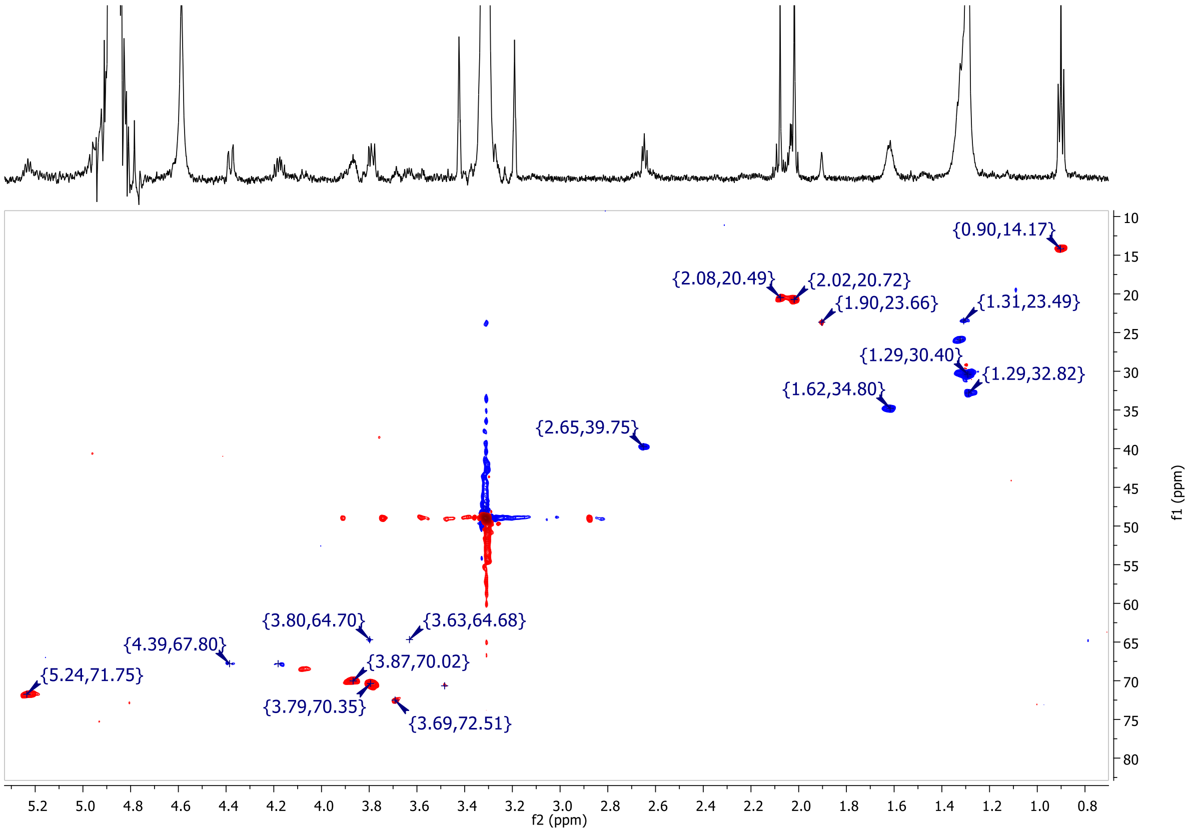

Figure S19. COSY spectrum of compound **2** (MeOD, 600 MHz)

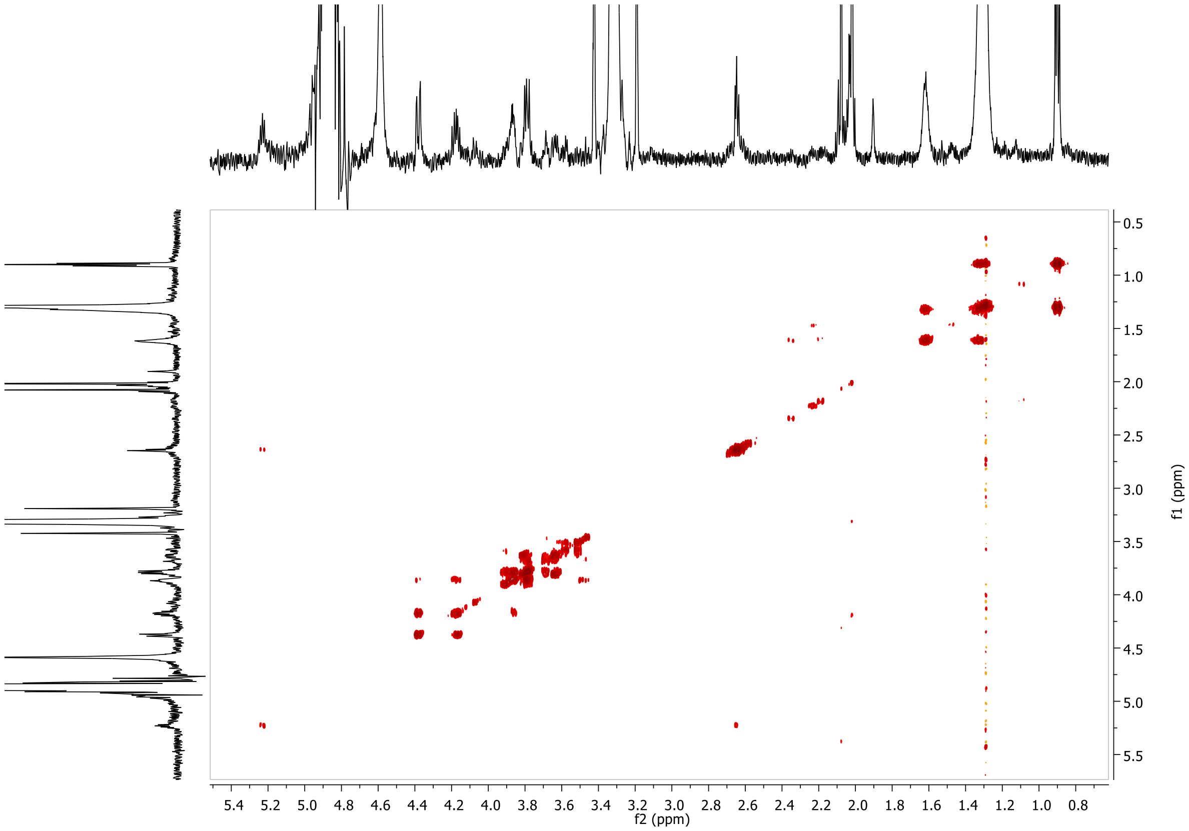

Figure S20. NOESY spectrum of compound **2** (MeOD, 600 MHz)

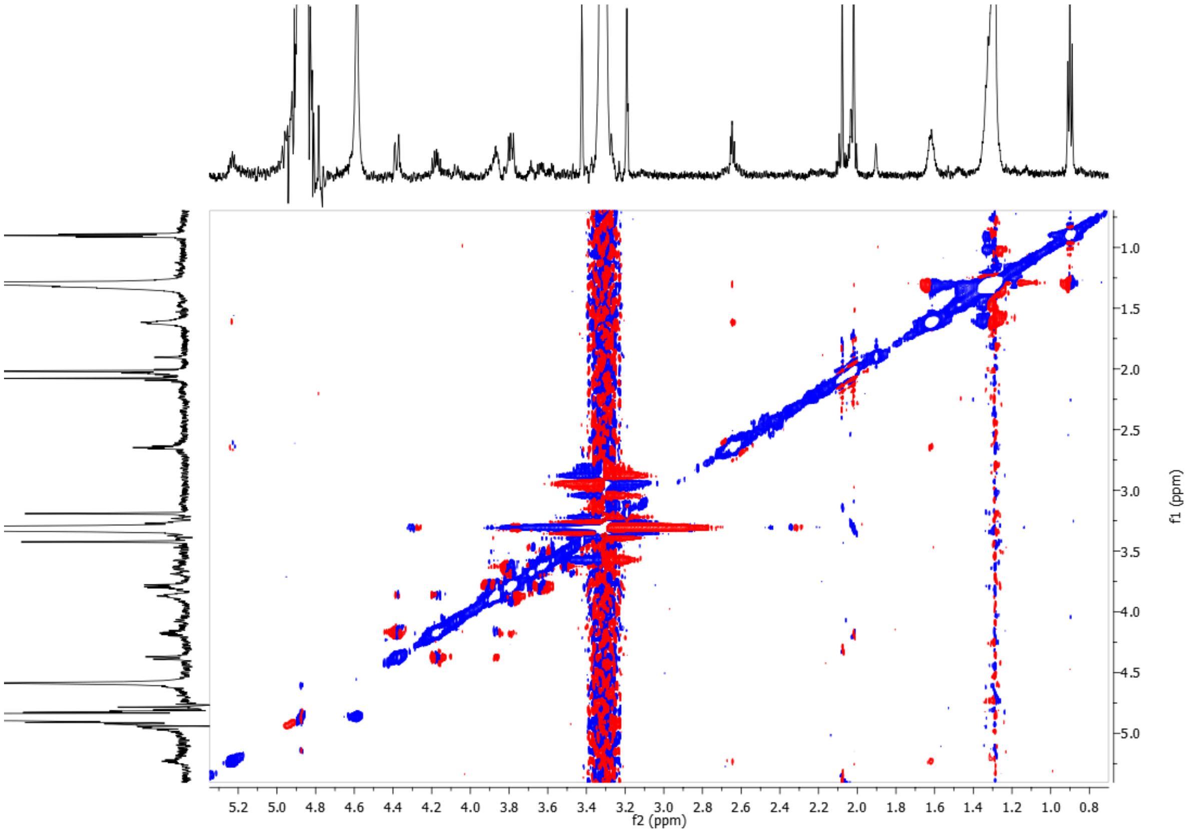

Figure S21. HMBC spectrum of compound **2** (MeOD, 600/150 MHz)

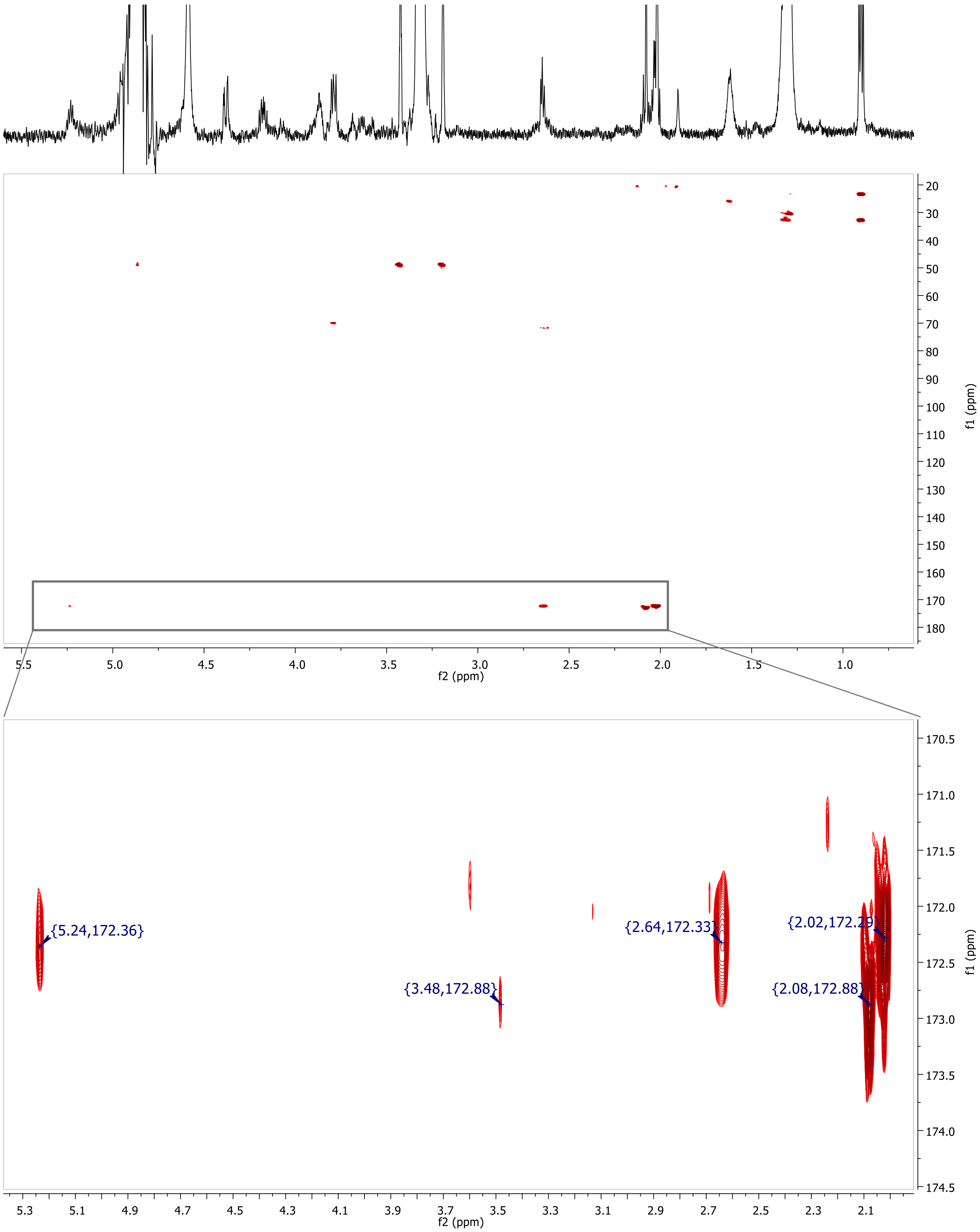

Figure S22. (a) HR-ESIMS and (b) MS/MS spectra of **3**

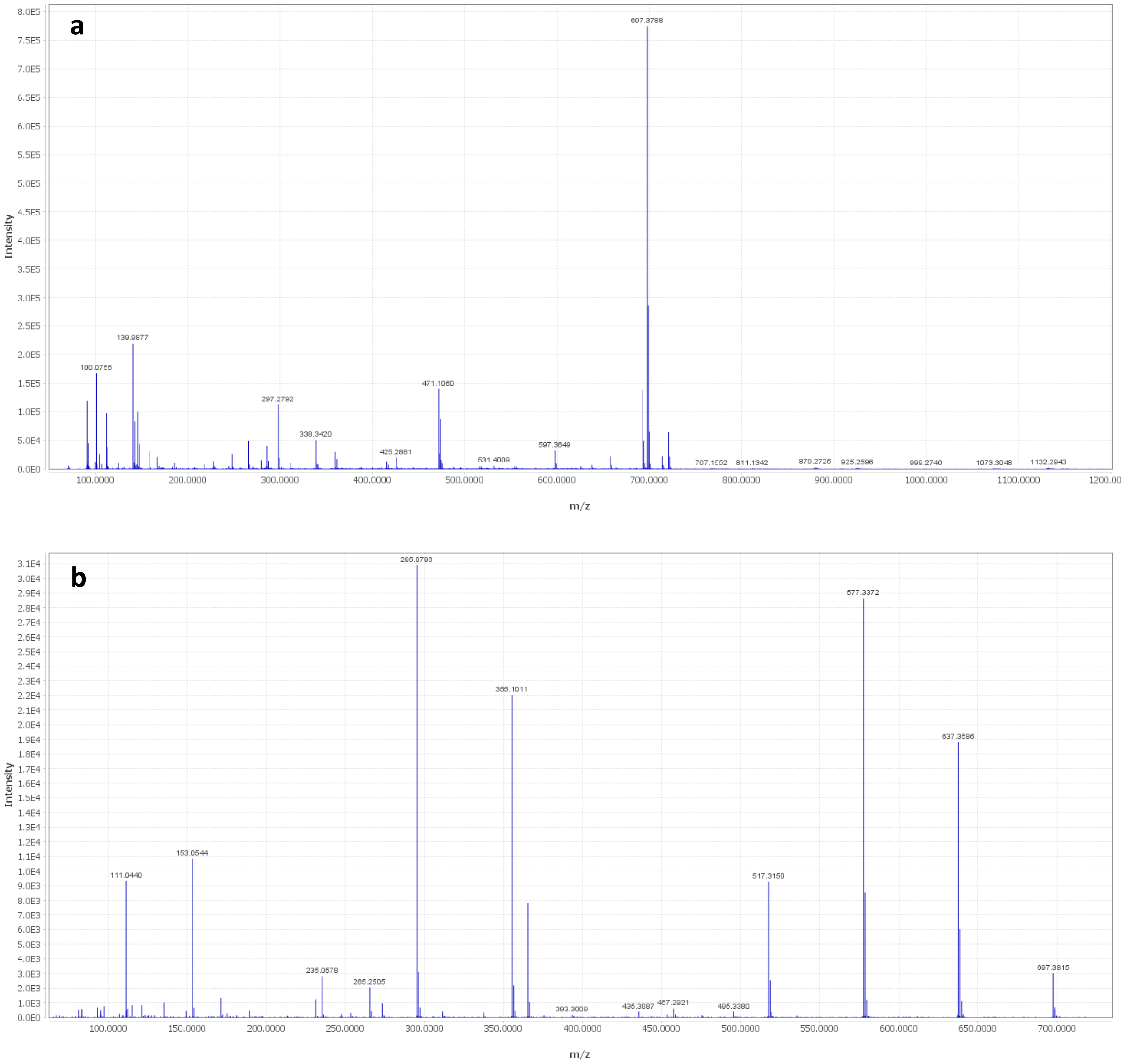

Figure S23. (a) HR-ESIMS and (b) MS/MS spectra of **4**

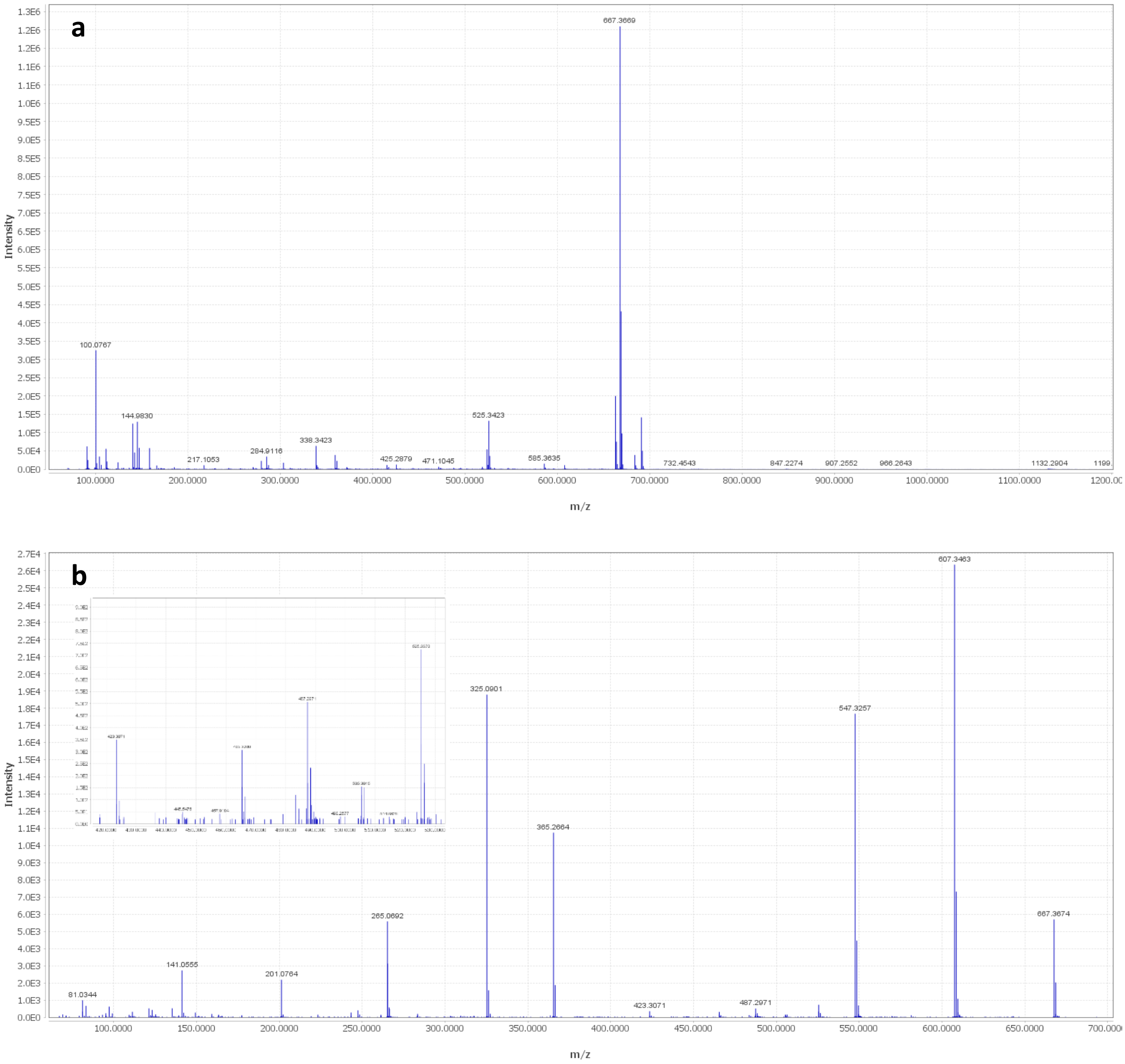

Figure S24. (a) HR-ESIMS and (b) MS/MS spectra of **5**

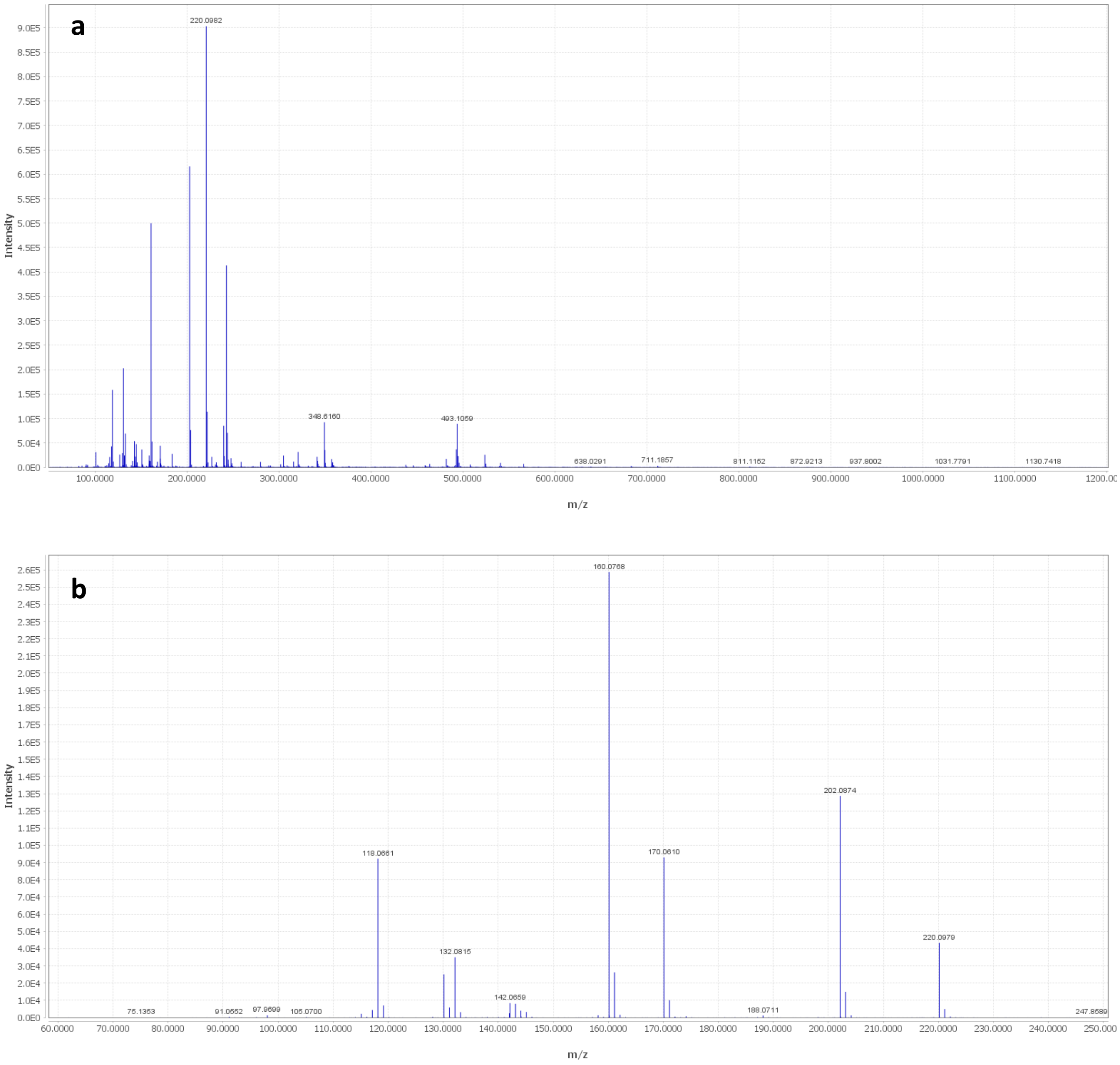

Figure S25. ^{1}H NMR spectrum of compound **5** (MeOD, 600 MHz)

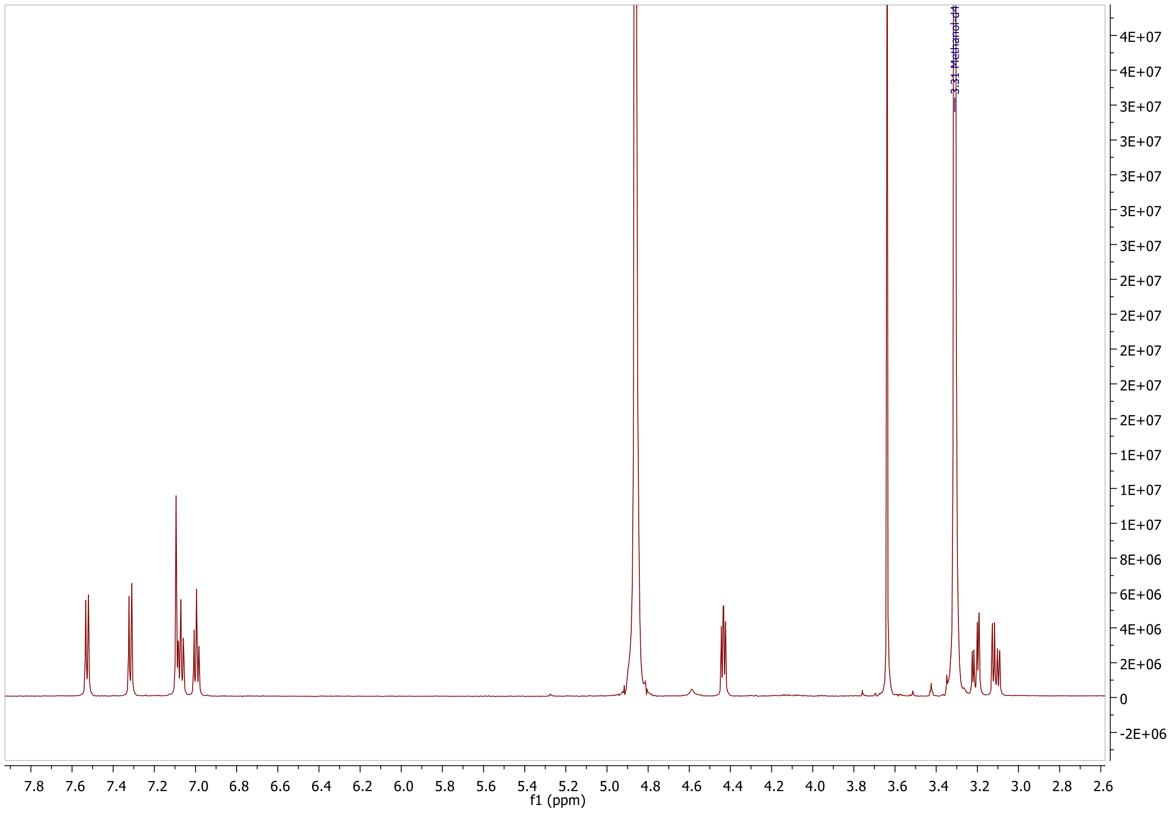

Figure S26. ^{13}C NMR spectrum of compound **5** (MeOD, 150 MHz)

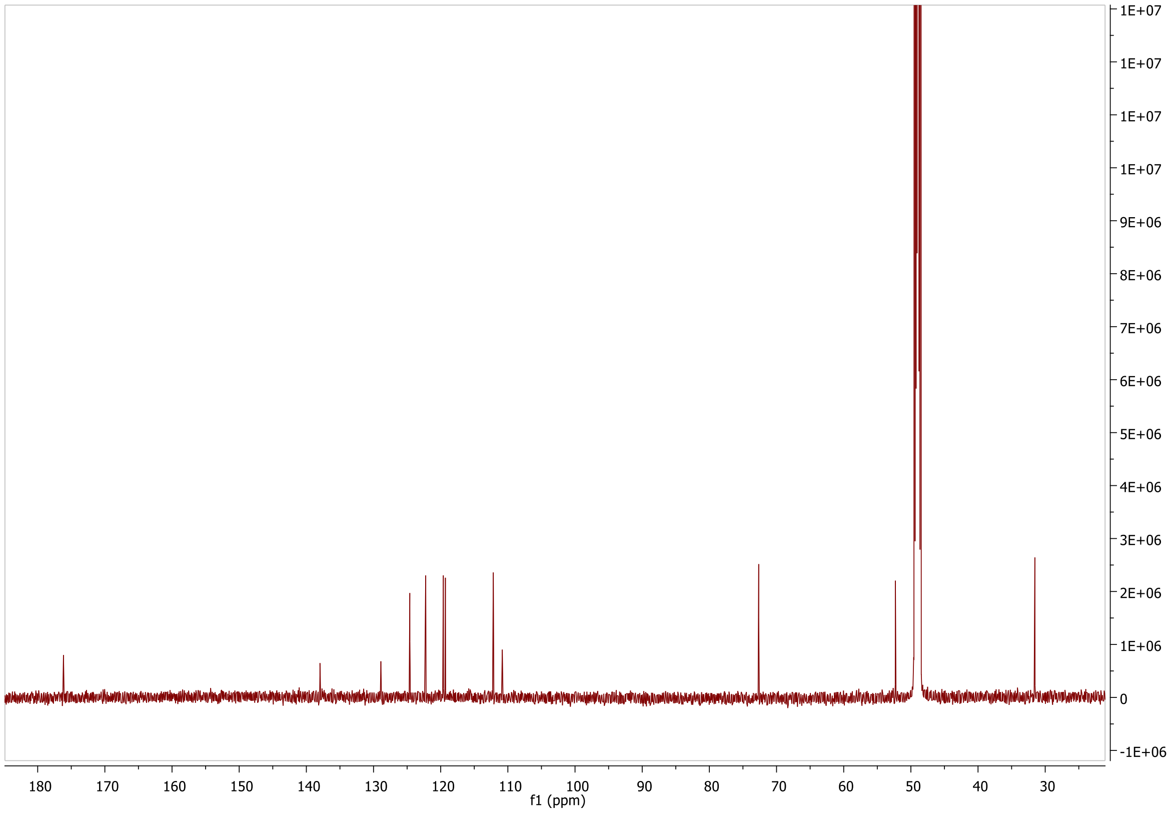

Figure S27. HSQC spectrum of compound **5** (MeOD, 600/150 MHz)

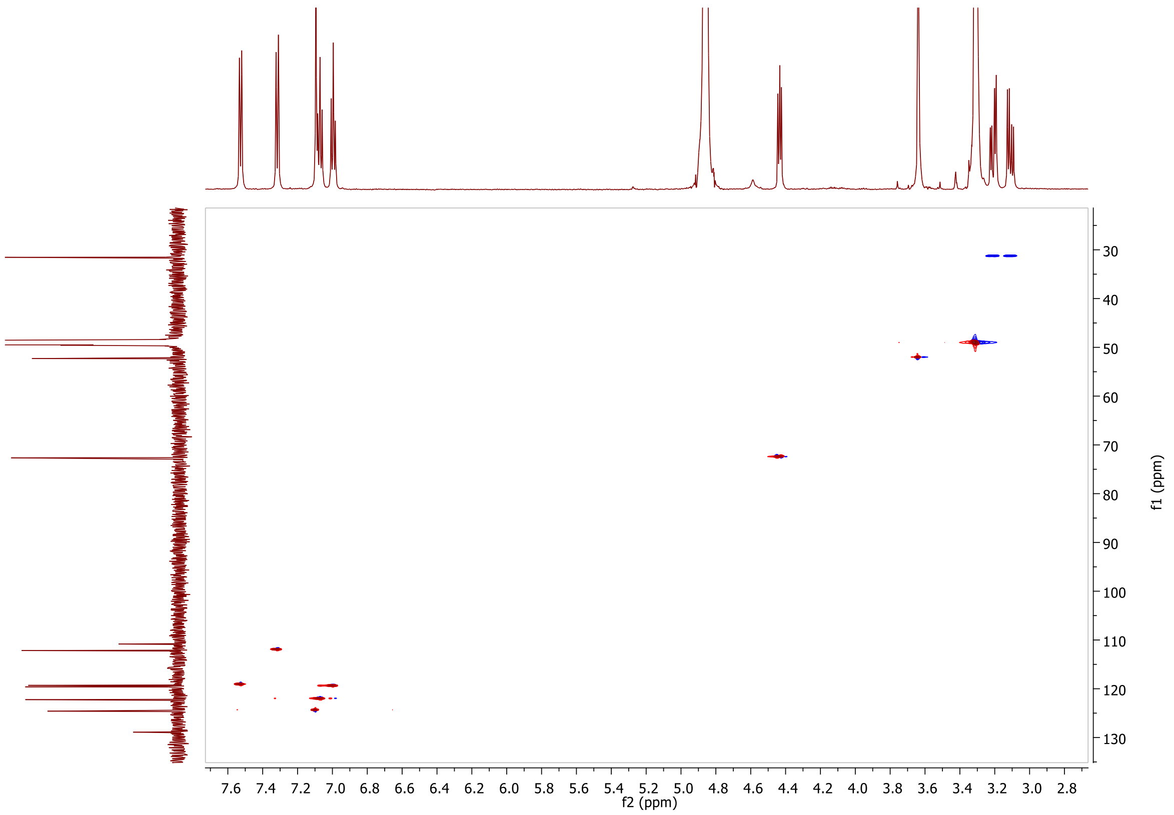

Figure S28. COSY spectrum of compound **5** (MeOD, 600 MHz)

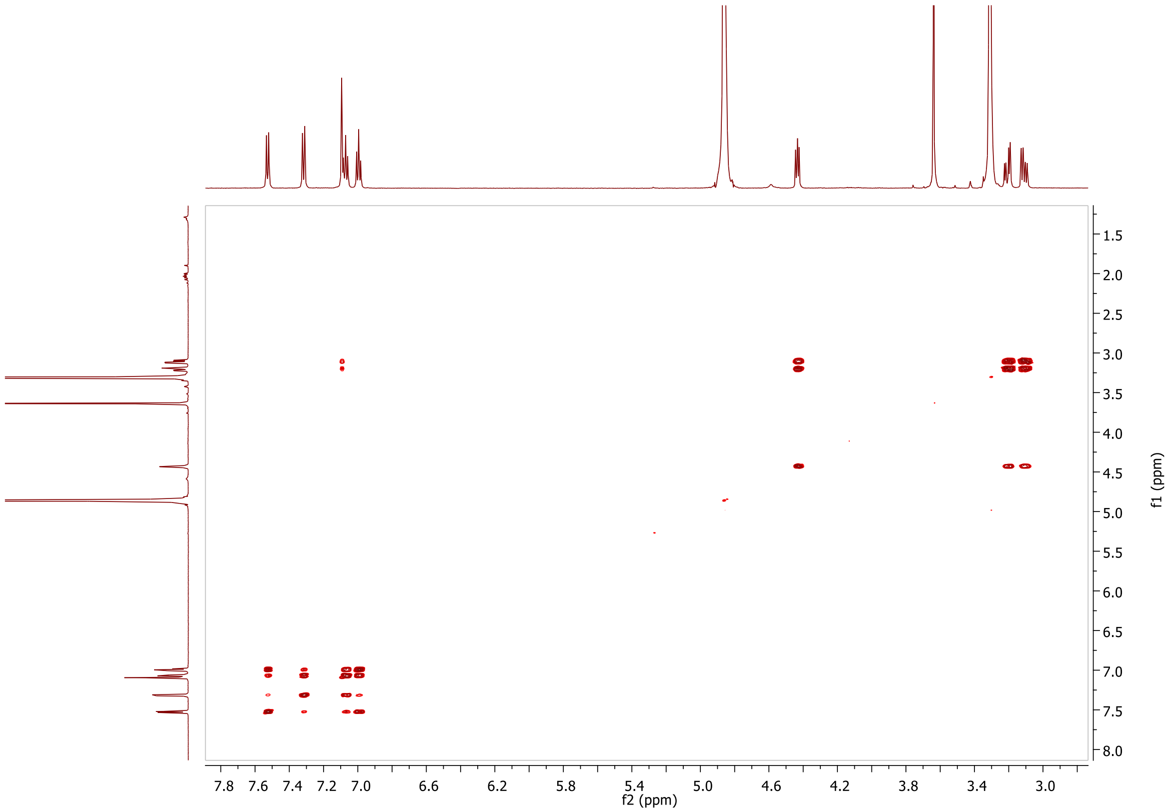

Figure S29. HMBC spectrum of compound **5** (MeOD, 600/150 MHz)

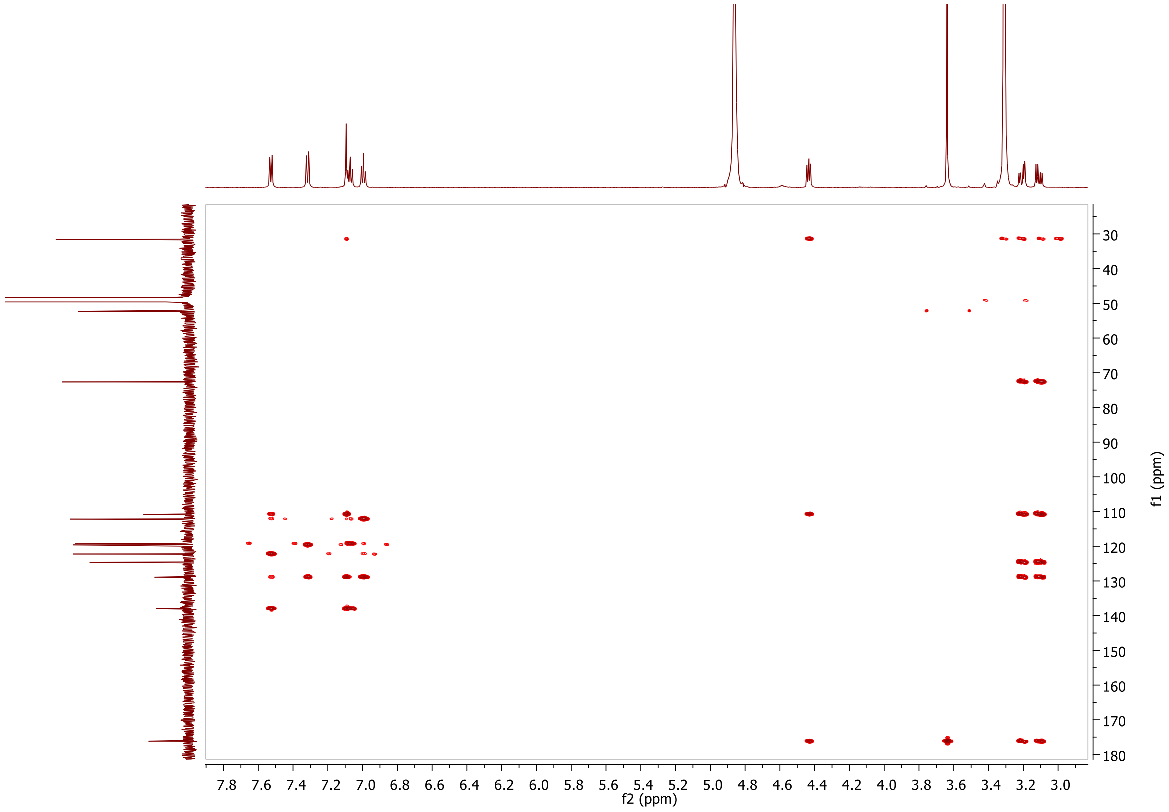

Figure S30. NOESY spectrum of compound **5** (MeOD, 600 MHz)

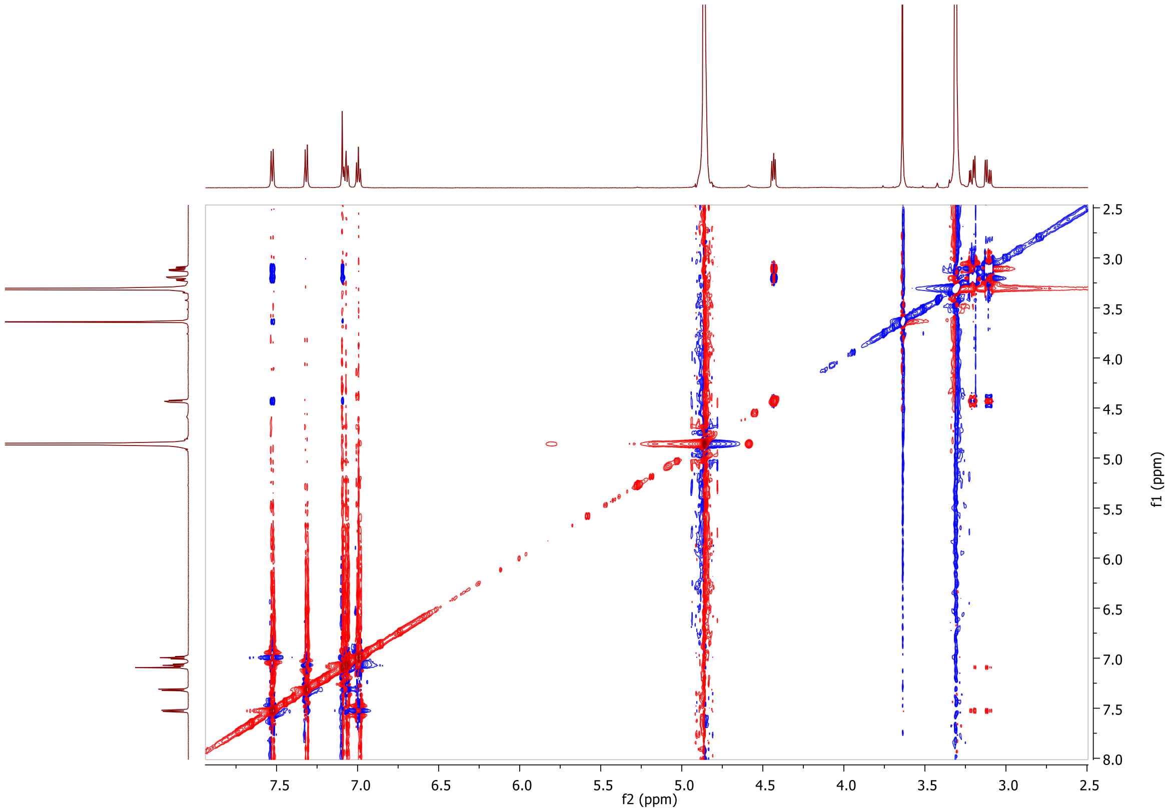

Figure S31. Metabolites reported in the Dictionary of Natural Products for the genus *Rhodotorula*

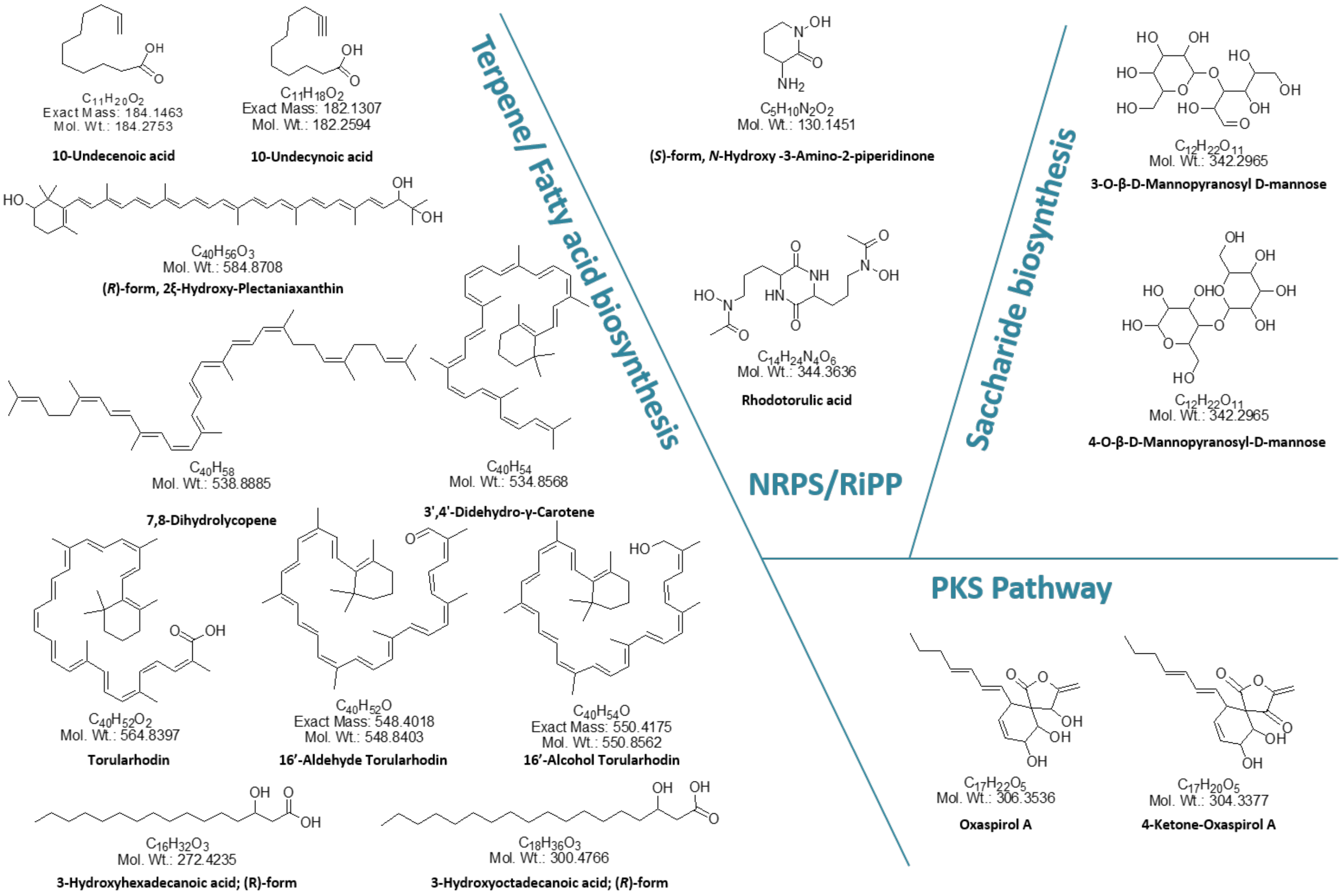

References

1. Sumner, L.W.; Amberg, A.; Barrett, D.; Beale, M.H.; Beger, R.; Daykin, C.A.; Fan, T.W.-M.; Fiehn, O.; Goodacre, R.; Griffin, J.L. Proposed minimum reporting standards for chemical analysis. *Metabolomics* **2007**, *3*, 211-221.
2. Blaženović, I.; Kind, T.; Ji, J.; Fiehn, O. Software tools and approaches for compound identification of LC-MS/MS data in metabolomics. *Metabolites* **2018**, *8*, 31. https://doi.org/10.3390/metabo8020031
3. Szafranek, J.; Palacz, Z.; Grzonka, Z. A comparison of electron impact and field ionization spectra of some 2, 5-diketopiperazines. *Org. Mass Spectrom.* **1976**, *11*, 920-930.
4. Manna, S.K.; Patterson, A.D.; Yang, Q.; Krausz, K.W.; Idle, J.R.; Fornace Jr, A.J.; Gonzalez, F.J. UPLC–MS-based urine metabolomics reveals indole-3-lactic acid and phenyllactic acid as conserved biomarkers for alcohol-induced liver disease in the Ppara-null mouse model. *J. Proteome Res.* **2011**, *10*, 4120-4133.
5. Kaiser, P.; Geyer, R.; Surmann, P.; Fuhrmann, H. LC–MS method for screening unknown microbial carotenoids and isoprenoid quinones. *J. Microbiol. Methods* **2012**, *88*, 28-34.
6. Cimmino, A.; Nocera, P.; Linaldeddu, B.T.; Masi, M.; Gorecki, M.; Pescitelli, G.; Montecchio, L.; Maddau, L.; Evidente, A. Phytotoxic metabolites produced by *Diaporthella cryptica*, the causal agent of hazelnut branch canker. *J. Agric. Food. Chem.* **2018**, *66*, 3435-3442.
7. Wang, J.-F.; Huang, Y.-J.; Fang, M.-J.; Xie, W.-L.; Su, W.-J.; Zhao, Y.-F. 5α, 8α-Epidioxyergosta-6, 22-dien-3β-ol (ergosterol peroxide) methanol solvate. *Acta Crystallogr. Sect. Sect. E: Struct. Rep. Online* **2004**, *60*, o764-o765. https://doi.org/10.1107/S160053680400813X
8. Rana, N.A.; Singh, A.; Del Poeta, M.; Hannun, Y.A. Qualitative and quantitative measurements of sphingolipids by mass spectrometry. In *Bioactive Sphingolipids in Cancer Biology and Therapy*, Springer: 2015; pp. 313-338.
9. Wang, M.; Mao, W.; Wang, X.; Li, F.; Wang, J.; Chi, Z.; Chi, Z.; Liu, G. Efficient simultaneous production of extracellular polyol esters of fatty acids and intracellular lipids from inulin by a deep-sea yeast *Rhodotorula paludigena* P4R5. *Microb. Cell Factories* **2019**, *18*, 149. https://doi.org/10.1186/s12934-019-1200-3
10. Garay, L.A.; Sitepu, I.R.; Cajka, T.; Fiehn, O.; Cathcart, E.; Fry, R.W.; Kanti, A.; Nugroho, A.J.; Faulina, S.A.; Stephanandra, S. Discovery of synthesis and secretion of polyol esters of fatty acids by four basidiomycetous yeast species in the order Sporidiobolales. *J. Ind. Microbiol. Biotechnol.* **2017**, *44*, 923-936.
11. Cajka, T.; Garay, L.A.; Sitepu, I.R.; Boundy-Mills, K.L.; Fiehn, O. Multiplatform mass spectrometry-based approach identifies extracellular glycolipids of the yeast *Rhodotorula babjevae* UCDFST 04-877. *J. Nat. Prod.* **2016**, *79*, 2580-2589.
